# Intestinal Clock Promotes Cognitive Memory Through Adenosine Signaling

**DOI:** 10.1002/advs.202504526

**Published:** 2025-06-20

**Authors:** Min Chen, Fugui Zhang, Yifei Xiao, Xuejun Jiang, Zhiqian Yan, Jinyi Wang, Xiting Lv, Jialu Cui, Linna Ha, Hui Chen, Yongkang Dang, Zifei Qin, Jing Yang, Baojian Wu

**Affiliations:** ^1^ Institute of Molecular Rhythm and Metabolism Guangzhou University of Chinese Medicine Guangzhou 510006 China; ^2^ Department of Pharmacy the First Affiliated Hospital of Zhengzhou University Zhengzhou 450052 China; ^3^ Department of Public Health and Preventive Medicine School of Medicine Jinan University Guangzhou 510632 China; ^4^ State Key Laboratory of Traditional Chinese Medicine Syndrome Guangzhou University of Chinese Medicine Guangzhou 510006 China

**Keywords:** adenosine, circadian rhythm, cognitive memory, intestinal clock

## Abstract

Although the intestinal clock (the circadian timing system in the gastrointestinal tract) is known to direct a wide variety of diurnal nutrients and metabolites, its role in the functioning of extra‐intestinal tissues such as the brain remains elusive. Here the role of the intestinal clock in shaping cognitive function is investigated. It is found that *Bmal1*‐iKO mice (mice with *Bmal1* [*Brain and muscle Arnt‐like protein 1*] specifically knocked out in the intestine, a mouse line deficient in intestinal clock function) show a defect in cognitive memory irrespective of the time‐of‐day. *Bmal1*‐iKO‐associated cognitive decline is attributed to impaired adenosine signaling and compromised long‐term potentiation (LTP) in the hippocampus. Adenosine signaling promotes LTP via enhancing BDNF expression and inhibiting synapse loss. Furthermore, the impairment in adenosine signaling is accounted for by the reductions in intestinal absorption of and hippocampal level of adenosine but not by a change in adenosine receptors. Consistently, adenosine supplementation rescues cognitive deficits associated with the malfunction of the intestinal clock. Moreover, BMAL1 regulates the expression of ADK (adenosine kinase, a primary enzyme for adenosine clearance) in the small intestine and thus promotes intestinal adenosine absorption through REV‐ERBα which binds directly to *Adk* P2 promoter to inhibit its transcription. Together, an unsuspected role of the intestinal clock in controlling cognitive memory is identified, highlighting the intestinal clock as a promising target for the management of cognitive disorders.

## Graphical abstract

The intestinal clock controls the expression of an adenosine enzyme that modulates systemic adenosine level and A_1_R signaling in the hippocampus, and in turn, cognitive function involving long‐term potentiation and BDNF‐dependent synaptic changes. This study represents a significant advancement in the understanding of the gut‐brain axis and its implications for cognitive health.

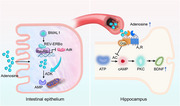

## 1 Introduction

Many aspects of physiology and biological behaviors show distinct diurnal (24 h) oscillations. These rhythms are primarily directed by circadian clocks which allow organisms to anticipate the environmental changes on a daily basis (e.g., the light‐dark cycle).^[^ ^1^ ^]^ The circadian timing system in mammals consists of a light‐entrainable master clock located in the suprachiasmatic nucleus (SCN) of the hypothalamus and numerous subordinate clocks present in other brain regions and in peripheral tissues such as the liver, pancreas, kidney, and lung.^[^ ^2^ ^]^ Orchestration of the subordinate clocks by the SCN clock involves hormonal and neuronal mechanisms.^[^ ^2^ ^]^ At the molecular level, these clocks are composed of multiple core clock genes (such as *Bmal1*, *Clock*, *Per*, *Cry*, *Rev‐erb*, and *Ror*) that form auto‐regulatory interlocking feedback loops to drive the circadian oscillations in their own expression and the expression of clock‐gated output genes.^[^ ^3^ ^]^ Through these output genes, the circadian system regulates various behaviors and physiology, such as the sleep‐wake cycle, feeding, metabolism, immun responses, hormone synthesis, and secretion.^[^ ^4^, ^5^ ^]^

Cognitive processes (such as learning and memory) are known to be gated by the circadian system.^[^ ^6^ ^]^ Two lines of straightforward evidence for this are: cognitive capacity varies as a function of the time‐of‐day; and disruptions of circadian timing have a negative effect on cognitive performance.^[^ ^7^, ^8^, ^9^ ^]^ In fact, core clock genes (e.g., *Bmal1*, *Per2*, and *Cry*) are expressed in cortical and limbic regions that underlie cognitive functions, including the hippocampus and amygdala.^[^ ^10^, ^11^, ^12^ ^]^ Conditional knockout of *Bmal1* in excitatory neurons of the forebrain leads to marked deficits in cognitive behaviors.^[^ ^13^ ^]^ Thus, extra‐SCN oscillators within forebrain circuits are critical for the circadian gating of cognition in addition to the SCN clock. Circadian gating of cognition involves modulation of cellular excitability and synaptic plasticity, although the exact molecular mechanisms remain elusive.^[^ ^14^, ^15^, ^16^ ^]^ Nevertheless, several signaling pathways and proteins (e.g., cAMP, PKA, ERK, and GSK3) have been identified to be implicated in the clock shaping of cognitive capacity.^[^ ^17^, ^18^, ^19^ ^]^ Cognitive impairment is a core feature of serious mental disorders (e.g., delirium and Alzheimer's disease), and also displays time‐of‐day variations. For instance, cognitive impairment in delirious conditions is more severe in the rest phase than in the activity phase, due to diurnal variations in microglial activation and ensuing neuroinflammation under the control of the clock component E4BP4.^[^ ^20^ ^]^ In line with the essential role of the circadian system in controlling cognition, small‐molecule modifiers of clock components (e.g., SR8278 for REV‐ERB and nobiletin for ROR) confer a neuroprotective effect and ameliorate cognitive impairments.^[^ ^20^, ^21^ ^]^

The intestine is thought to be one of the organs affected the most by circadian rhythms as clock disruption (e.g., by shift work or jet lag) has been associated with an increased risk for intestinal symptoms and diseases such as abdominal pain and irritable bowel syndrome.^[^ ^22^ ^]^ As such, it is not surprising that a wide variety of physiological processes in the gut (such as intestinal mobility, nutrient digestion and absorption, drug disposition and absorption, epithelial barrier function, and gut microbiome) are subjected to prominent circadian variations.^[^ ^23^ ^]^ In a recent report, we demonstrate that the intestinal clock reprograms the rhythmic transcriptome of the liver (and other peripheral tissues such as the kidney and white adipose tissue), and one of the underlying mechanisms involves the modulation of intestinal metabolism and the levels of polyunsaturated fatty acids.^[^ ^24^ ^]^ This highlights the potential role of the intestinal clock in shaping the metabolome and transcriptome as well as the functions of extra‐intestinal organs.^[^ ^24^ ^]^ Given that a wide range of nutrients and metabolites (with broad biological functions) are gated by an intestinal clock,^[^ ^25^, ^26^ ^]^ several critical questions remain to be answered: does the intestinal clock have a role in maintaining brain functions? Whether and how metabolite‐based diurnal signals from the gut shape the rhythmicity in brain functions? Does shaping of brain functions by diurnal metabolites depend on the liver?

Although circadian mechanisms are known to modulate cognition, the contribution of the gut‐specific circadian clock to cognitive processes has yet to be elucidated. Here we investigate the role of the intestinal clock in shaping cognitive capacity. We find that *Bmal1*‐iKO mice (mice with *Bmal1* specifically knocked out in the intestine, a mouse line deficient in intestinal clock function) show a defect in cognitive memory. The cognitive decline is attributed to compromised LTP (long‐term potentiation) in the hippocampus caused by a reduced adenosine tone and impaired adenosine‐A_1_R signaling. Adenosine‐A_1_R signaling promotes LTP via enhancing BDNF expression and inhibiting synapse loss. Moreover, intestinal BMAL1 negatively regulates the expression of adenosine kinase (ADK, a major adenosine‐removing enzyme) and adenosine absorption through REV‐ERBα which binds directly to the *Adk* P2 promoter to inhibit its transcription. Overall, we identify an unsuspected role of the intestinal clock in controlling cognitive memory. Targeting the intestinal clock opens a new avenue for the management of cognitive disorders.

## 2 Results

### 2.1 Deficiency of Intestinal Clock Results in Cognitive Deficits Independent of Gut Microbiome

**Figure 1:**
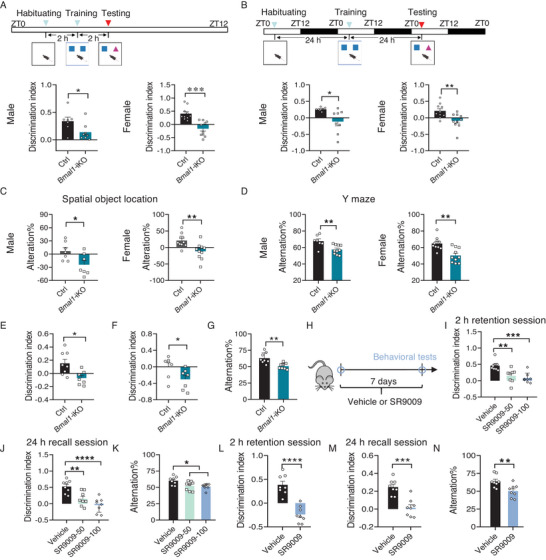
Malfunction of the intestinal clock causes cognitive deficits in mice. A) Diagram of the experimental design for assessing short‐term memory (top). Discrimination index in NOR test during 2 h retention session in male and female *Bmal1*‐iKO and control mice (*n* = 7–10, bottom). B) Diagram of the experimental design for assessing long‐term memory (top). Discrimination index in NOR during 24 h recall session in male and female *Bmal1*‐iKO and control mice (*n* = 8–10, bottom). C) Performance on SOL test in male and female *Bmal1*‐iKO and control mice (*n* = 7–10). D) Spontaneous alternations in Y maze test in male and female *Bmal1*‐iKO and control mice (*n* = 7–10). E) Discrimination index in NOR test during 2 h retention session in *Bmal1*‐iKO and control mice (*n* = 7–8) at an age of 23–27 days. F) Discrimination index in NOR test during 24 h recall session in *Bmal1*‐iKO and control mice (*n* = 7) at an age of 23–27 days. G) Spontaneous alternations in Y maze test in *Bmal1*‐iKO and control mice (*n* = 8) at an age of 23–27 days. H) Timeline schematic for SR9009 treatment and behavioral tests. I) Discrimination index in NOR test during 2 h retention session in male SR9009‐ and vehicle‐treated mice (*n* = 7–8). J) Discrimination index in NOR test during 24 h recall session in male SR9009‐ and vehicle‐treated mice (*n* = 8). K) Spontaneous alternations in Y maze test in male SR9009‐ and vehicle‐treated mice (*n* = 8). L) Discrimination index in NOR test during 2 h retention session in female SR9009 (100 mg kg^−1^)‐ and vehicle‐treated mice (*n* = 7–8). M) Discrimination index in NOR test during 24 h recall session in female SR9009 (100 mg kg^−1^)‐ and vehicle‐treated mice (*n* = 7–8). N) Spontaneous alternations in Y maze test in female SR9009 (100 mg kg^−1^)‐ and vehicle‐treated mice (*n* = 8). Discrimination indices were calculated as: (Time for novel object exploring – time for familiar object exploring)/(Time for novel object exploring + time for familiar object exploring). All behavioral tests were conducted at ZT6. Data are mean ± SEM, and analyzed by two‐tailed Student's t‐test (A–G and L–N), and one‐way ANOVA followed by Bonferroni posttest (I–K). ^*^ *p*  <  0.05, ^**^ *p*  <  0.01, ^***^ *p* < 0.001 and ^****^ *p* < 0.0001.

To clarify the potential effects of the intestinal clock on cognitive function, *Bmal1*‐iKO mice (mice with *Bmal1* [a central clock gene] specifically knocked out in the intestine, showing a defect in intestinal clock function) were generated as described previously.^[^ ^24^, ^27^ ^]^ Novel object recognition (NOR) tests were performed to assess short‐term memory (a 2 h interval between NOR testing and training) and long‐term memory (a 24 h interval between NOR testing and training) (**Figure** **1**A,B). Intriguingly, male *Bmal1*‐iKO mice (aged at 6–8 weeks) showed impaired short‐term memory as evidenced by a loss of novel object preference (Figure 1A). Likewise, long‐term memory was compromised in these knockouts (Figure 1B). The spatial object location (SOL) and Y maze tests were performed to assess working memory. In the SOL test, control (*Bmal1*‐flox) but not male *Bmal1*‐iKO mice showed preferential exploration of the novel location, indicative of compromised object place recognition memory upon *Bmal1*‐iKO (Figure 1C). Meanwhile, male *Bmal1*‐iKO mice had a lower rate of spontaneous alternations in the Y maze test (Figure 1D). Furthermore, female *Bmal1*‐iKO mice demonstrated cognitive deficits, indicating that the cognitive phenotypes were gender‐independent (Figure 1A–D). The locomotion was not altered in *Bmal1*‐iKO mice (as reflected by total exploration time and total distance in the NOR test and total number of arm entries in the Y maze; Figure S1A,B, Supporting Information). We also examined whether the effects of intestinal *Bmal1* on memory occur in an early stage of life. Cognitive deficiency was observed in *Bmal1*‐iKO mice at an age of 23–27 days (Figure 1E,G). The intestinal microbiota is known to modulate brain functions including cognitive function.^[^ ^28^ ^]^ We thus wondered whether the microbiota has a role in the regulation of cognitive behaviors by intestinal *Bmal1*. We found that loss of microbiota (germ‐free, GF) did not affect an abrogation of cognitive function in *Bmal1*‐iKO mice (Figure S1C–E, Supporting Information). Supporting this, *Bmal1*‐iKO‐induced cognitive deterioration persisted when mice were treated with an antibiotic cocktail (ABX, a chemical method to deplete gut microbiota) (Figure S1F–H, Supporting Information). Microbiota‐ablated wild‐type mice demonstrated deficits in cognitive behaviors as compared to conventionally raised SPF mice (Figure S1C–H, Supporting Information), consistent with a prior report.^[^ ^29^ ^]^ Therefore, we can exclude a major role of the microbiome in intestinal *Bmal1*‐controlled cognitive behaviors. However, whether intestinal *Bmal1* and microbiota interact to regulate cognition remains to be clarified. Because cognitive capacity varies as a function of time‐of‐day, we tested whether intestinal *Bmal1* modulates time‐of‐day dependent cognition. As expected, control mice showed a time‐of‐day variation in cognitive memory with a peak value at the dark: light transition (Figure S1I,J, Supporting Information). Similar results were observed when mice were maintained under constant darkness (Figure S1K,L, Supporting Information). Loss of intestinal *Bmal1* led to cognitive dysfunction in mice throughout the light‐dark cycle (and in mice kept under constant darkness), however, the cognitive rhythmicity was retained (Figure S1I–L and Table S1, Supporting Information). Therefore, intestinal *Bmal1* has a role in maintaining cognitive capacity without affecting much of its oscillation. We also noted that intestinal *Bmal1* deficiency was associated with despair‐like behaviors (based on forced swimming and tail suspension tests) but did not affect euphoria (assessed by novelty‐suppressed feeding test), anxiety (assessed by elevated plus maze and open filed tests) and social activity (assessed by three‐chamber social test) (Figure S2A–F, Supporting Information). It was worth noting that *Bmal1*‐iKO did not affect the sleep‐wake cycle and sleep architecture either (Figure S2G–I, Supporting Information). Thus, alteration of cognitive function in *Bmal1*‐iKO mice was not due to a change in sleep.

Given that genetic disruption of intestinal *Bmal1*, and in effect clock function, resulting in cognitive dysfunction, we next tested whether chemical perturbance of intestinal BMAL1 has a similar effect. We used SR9009 (a synthetic agonist of REV‐ERBɑ which binds to *Bmal1* promoter and inhibits its transcription; 50 and 100 mg kg^−1^, oral gavage) to specifically down‐regulate intestinal BMAL1 expression (Figure S2J–L, Supporting Information),^[^ ^24^, ^27^ ^]^ and assessed its effects on cognitive behaviors (Figure 1H). Unsurprisingly, SR9009‐treated male mice showed cognitive deficits, as evidenced by a loss of novel object preference in NOR tests and a reduced rate of spontaneous alternations in Y maze tests (Figure 1I,K). Likewise, cognitive impairment was observed in female mice treated with SR9009 (100 mg kg^−1^, Figure 1L,N). These data corroborated that intestinal *Bmal1* has a critical role in regulating cognitive function.

Cognitive impairment is a core feature of serious mental disorders such as delirium.^[^ ^20^ ^]^ We asked whether intestinal *Bmal1* has a role in the development of delirium. To test this, *Bmal1*‐iKO and control mice were treated with lipopolysaccharide plus midazolam (named LM treatment) to induce delirium.^[^ ^20^ ^]^ In comparison with control mice, *Bmal1*‐iKO mice showed an increased susceptibility to developing delirium as evidenced by a lower discrimination index (between the novel and familiar object) and fewer spontaneous alternations (Figure S2N,O, Supporting Information). In a different paradigm, wild‐type mice were gavaged with SR8278 for 7 days to specifically increase intestinal BMAL1 expression, and then delirium induction was initiated (Figure S2L,M, Supporting Information). SR8278‐treated mice showed a higher discrimination index and more extensive spontaneous alternations across the 24 h cycle (reflective of delirium alleviation) as compared to vehicle‐treated counterparts (Figure S2P,Q, Supporting Information). In sharp contrast, the effects of SR8278 on delirium development were lost in *Bmal1*‐iKO mice (Figure S2P,Q, Supporting Information). These findings suggested that through modulating cognition intestinal *Bmal1* may regulate the development of mental disorders.

### 2.2 Impairment of Hippocampal Long‐Term Potentiation (LTP) Underlies *Bmal1*‐iKO‐Associated Cognitive Decline

**Figure 2:**
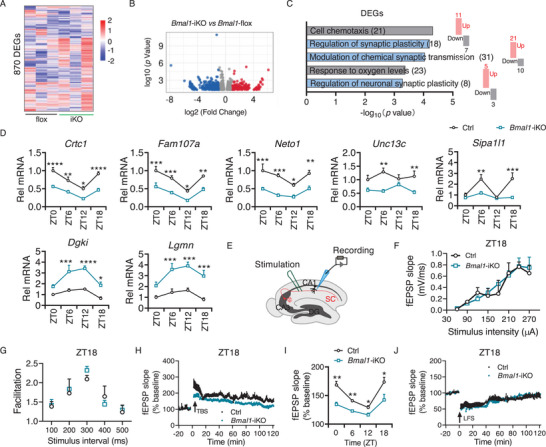
Loss of intestinal *Bmal1* impairs hippocampal LTP in mice. A) Heatmap for differentially expressed genes (DEGs) in the hippocampus caused by *Bmal1‐*iKO (*n* = 3). B) Volcano plot showing differential gene expression. C) GO enrichment analysis of hippocampal DEGs associated with *Bmal1‐*iKO. D, qPCR analyses of synaptic plasticity‐related genes in the hippocampus from *Bmal1*‐iKO and control mice at different time points (*n* = 6). E) Schematic illustration of the experimental configuration for LTP. The Schaffer collateral pathway (SC, red) was stimulated, and field potentials were recorded in the CA1 region of the hippocampus. LTP was induced by theta‐burst stimulation (TBS). DG, dentate gyrus; CA, cornu ammonis. F) Input/output curves in hippocampal slices from *Bmal1*‐iKO and control mice at ZT18 (*n* = 6). G) Paired‐pulse facilitation in hippocampal slices from *Bmal1*‐iKO and control mice at ZT18 (*n* = 6). H) Time course of fEPSP recordings in hippocampal slices from *Bmal1*‐iKO and control mice after TBS at ZT18‐20 (*n* = 4). I) Average normalized fEPSP slope for hippocampal slices from *Bmal1*‐iKO and control mice at different time intervals (*n* = 3–4). J) Time course of fEPSP recordings in hippocampal slices from *Bmal1*‐iKO and control mice after a low‐frequency stimulation (LFS) at ZT18‐20 (*n* = 3). In panel (I) fEPSP slopes were normalized to the 20 min baseline average (pre‐TBS). All data are mean ± SEM, and analyzed by two‐way ANOVA followed by Bonferroni posttest (D,I). ^*^ *p*  <  0.05, ^**^ *p*  <  0.01, ^***^ *p* < 0.001 and ^****^ *p* < 0.0001.

The hippocampus plays a pivotal role in memory formation and flexibility. To understand the mechanisms underlying the defective cognition and memory in intestinal clock‐deficient mice, we collected hippocampal samples from *Bmal1*‐iKO and control mice at 6 h into the dark period (zeitgeber time [ZT] 18, corresponding to midnight, when cognitive dysfunction is pronounced in the knockouts, Figure S1I–L, Supporting Information), and subsequently performed transcriptomic analyses. A large number of differentially expressed genes (870 DEGs) were identified between *Bmal1*‐iKO and the control hippocampus (**Figure** **2**A). Among these 371 genes were up‐regulated in *Bmal1*‐iKO mice, whereas 499 genes were down‐regulated (Figure 2B). A high number of DEGs converged on synaptic plasticity‐related pathways, such as regulation of synaptic plasticity, modulation of chemical synaptic transmission, and regulation of neuronal synaptic plasticity (Figure 2C). Transcriptomic analyses of hippocampal samples from the two genotypes collected at 6 h into the light period (ZT6, corresponding to midday) confirmed the enrichment of DEGs on synaptic plasticity‐related pathways (Figure S3A–C, Supporting Information). These findings suggested the involvement of synaptic plasticity in the regulation of cognition by intestinal *Bmal1*. In‐depth analyses of transcriptomic data revealed that *Bmal1*‐iKO led to down‐regulation of the genes (such as *Crtc1* ^[^ ^30^ ^]^ *Fam107a*,^[^ ^31^ ^]^ *Neto1*,^[^ ^32^ ^]^ *Unc13c* ^[^ ^33^ ^]^ and *Sipa1l1* ^[^ ^34^ ^]^) that positively regulate synaptic plasticity in the hippocampus, and to up‐regulation of the genes (*Dgki* and *Lgmn* ^[^ ^35^ ^]^) that negatively regulate synaptic plasticity (Figure S3D, Supporting Information). The expression changes in these genes due to *Bmal1*‐iKO at different diurnal time points were also confirmed by performing qPCR and immunoblotting assays (Figure 2D; Figure S3e, Supporting Information). These results indicated that synaptic plasticity was compromised in the hippocampus of *Bmal1*‐iKO mice.

Next, we investigated whether synaptic plasticity is indeed impaired in the knockouts. To this end, hippocampal LTP, a form of synaptic plasticity and closely linked to cognitive memory,^[^ ^36^ ^]^ was assessed in both *Bmal1*‐iKO and control mice. We performed field potential recordings in the dendritic area of the CA1 region after stimulation of the Schaffer collaterals of CA3 neurons (SC‐CA1 synapse) in hippocampal slices (Figure 2E). Throughout the 24 h cycle, *Bmal1*‐iKO did not affect basal synaptic transmission and presynaptic function related to calcium‐dependent neurotransmitter release as evidenced by no changes in input‐output relationships (or I/O curves) at different stimulus intensities and in paired‐pulse facilitation (PPF) (Figure 2F,G; Figure S3F,G, Supporting Information). However, *Bmal1*‐iKO caused an impairment in hippocampal LTP independent of daily time as field excitatory postsynaptic potential (fEPSP) slope after theta‐burst stimulation (TBS) was significantly reduced (Figure 2H,I; Figure S3H, Supporting Information). In sharp contrast, long‐term depression (LTD), another form of synaptic plasticity, was unaffected by intestinal *Bmal1* deficiency (Figure 2J). Altogether, our results indicated that LTP may contribute to the memory impairment associated with intestinal clock deficiency.

### 2.3 Involvement of Adenosine Signaling in Regulation of Cognition by Intestinal *Bmal1*

**Figure 3:**
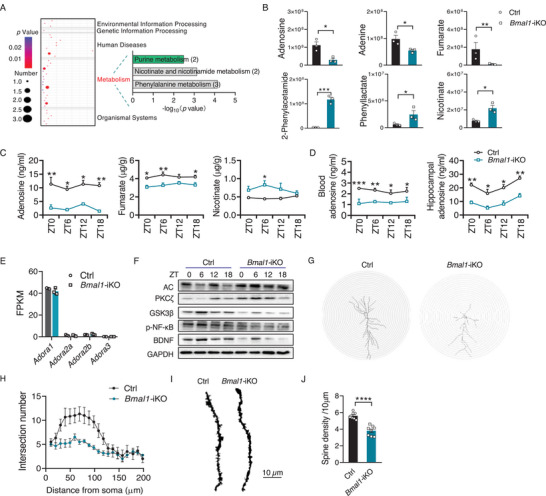
Involvement of adenosine‐A_1_R signaling in intestinal clock regulation of cognition. A) KEGG pathway analysis of differential metabolites in the small intestine from *Bmal1*‐iKO and control mice at ZT6 according to metabolomic experiments (*n* = 3). B) Peak areas of adenosine, fumarate, and nicotinate in the small intestine from *Bmal1*‐iKO and control mice at ZT6 (*n* = 3). C, Adenosine, fumarate, and nicotinate concentrations in the small intestine from *Bmal1*‐iKO and control mice based on LC‐MS/MS with multiple reaction monitoring (*n* = 6). D) Adenosine concentrations in the blood and hippocampus from *Bmal1*‐iKO and control mice based on LC‐MS/MS with multiple reaction monitoring (*n* = 6). E) Expression of adenosine receptors (*Adora1, Adora2a, Adora2b*, and *Adora3*) in the hippocampus from *Bmal1*‐iKO and control mice according to RNA‐seq (*n* = 3). F) Immunoblotting of hippocampal enzymes involved in adenosine‐A_1_R signaling in *Bmal1*‐iKO and control mice. For western blotting analysis of diurnal protein expression, three of nine samples from nine mice at each time point were pooled to generate three biological replicates. One representative blot is shown from three biological replicates. G) Representative Golgi‐stained CA1 pyramidal neurons, showing decreased total dendrite length in *Bmal1*‐iKO mice. H) Neurite arborization of CA1 pyramidal neurons in *Bmal1*‐iKO and control mice (*n* = 6). I) Representative images of dendritic branches from Golgi‐stained CA1 pyramidal neurons (scale bar, 10 µm). J) Spine density of the dendrites of CA1 pyramidal neurons in *Bmal1*‐iKO and control mice (*n* = 8). Mice were sacrificed and the samples were collected at ZT6. Data are mean ± SEM, and analyzed by two‐tailed Student's *t*‐test (B,J), and two‐way ANOVA followed by Bonferroni posttest (C,D). ^*^ *p*  <  0.05, ^**^ *p*  <  0.01, ^***^ *p* < 0.001 and ^****^ *p* < 0.0001.

Given that the intestinal clock can modulate hippocampal LTP and cognitive function, we next investigated the underlying mechanisms thereof. Gut‐derived nutrients and metabolites are known to play an important role in crosstalk between the intestine and the brain.^[^ ^28^ ^]^ We thus wondered whether these nutrients and metabolites are likely involved in the regulation of cognitive function by the intestinal clock. We performed metabolomic profiling of the intestinal samples from *Bmal1*‐iKO and control mice. Differential metabolites between *Bmal1*‐iKO and control mice were enriched in three types of metabolic pathways, namely, purine metabolism (affected metabolites: adenosine and adenine), nicotinate and nicotinamide metabolism (affected metabolites: nicotinate and fumarate) and phenylalanine metabolism (affected metabolites: fumarate, 2‐phenylacetamide and phenyllactate) according to Gene ontology (GO) enrichment analyses (**Figure** **3**A,B). Of these affected metabolites, adenosine, fumarate, and nicotinate are implicated in regulating brain cognitive functions.^[^ ^37^, ^38^, ^39^ ^]^ In line with the metabolomic data, LC‐MS/MS with multiple reaction monitoring confirmed that adenosine and fumarate were reduced, whereas the nicotinate level was increased in the small intestine (Figure 3C). Intriguingly, adenosine was consistently reduced in the systemic blood circulation and hippocampus in *Bmal1*‐iKO mice (Figure 3D). By contrast, fumarate and nicotinate were unaltered in the systemic blood and hippocampus (Figure S4A,B, Supporting Information). These data suggested that adenosine may participate in the regulation of cognition by an intestinal clock.

Adenosine, a potent neuromodulator, is known to maintain brain homeostasis through G protein‐coupled adenosine receptor (with four major subtypes such as A_1_R, A_2A_R, A_2B_R, and A_3A_R) neurotransmission. Consistent with a prior report,^[^ ^37^ ^]^ A_1_R was predominantly expressed in the hippocampus of control mice and A_2_R showed moderate expression, whereas A_3A_R had no expression (Figure 3E). Thus, the role of A_3A_R in adenosine signaling in the hippocampus can be excluded. A_1_R is coupled to inhibitory G_i_ protein and triggers intracellular cyclic AMP (cAMP)‐mediated signaling, which plays a critical role in regulating synapses and synaptic plasticity.^[^ ^37^, ^40^ ^]^ We found that the levels of AC (adenylate cyclase, the enzyme that catalyzes the formation of cAMP from ATP) and PKC (protein kinase C, a cAMP‐linked downstream mediator) were increased in the hippocampus of *Bmal1*‐iKO mice (Figure 3F; Figure S4C, Supporting Information). Meanwhile, intestinal *Bmal1* knockout led to decreases in hippocampal expression of GSK3β (glycogen synthase kinase 3β, which is inhibited by PKC), p‐NF‐κB (a downstream molecule of GSK3β), and BDNF (brain‐derived neurotrophic factor, a transcriptional target of NF‐κB) (Figure 3F; Figure S4C, Supporting Information).^[^ ^41^ ^]^ GSK3β is known to phosphorylate NF‐κB to facilitate its nuclear import and to promote its transcriptional actions on the target genes such as *Bdnf*.^[^ ^42^, ^43^ ^]^ We confirmed that the inactivation of PKC signaling caused elevations in the expression of GSK3β, p‐NF‐κB, and BDNF in HT22 cells (Figure S4H, Supporting Information). BDNF is an essential neurotrophic factor that positively modulates synaptic plasticity and axonal outgrowth.^[^ ^44^ ^]^ In accordance with a reduction of BDNF, the CA1 neurons of *Bmal1*‐iKO mice showed significantly decreased dendrite length and reduced spine density at both ZT6 and ZT18 according to Golgi staining (Figure 3G–J; Figure S4D–G, Supporting Information). Thereafter, we stereotaxically injected an AAV_1_ vector encoding *Bdnf* (AAV‐BDNF) into the hippocampus of *Bmal1*‐iKO mice to up‐regulate BDNF expression (Figure S4I, Supporting Information). We found that *Bmal1*‐iKO mice treated with AAV‐BDNF showed prolonged dendrite length and increased spine density as well as improved cognitive memory as compared to blank virus‐treated counterparts (Figure S4J–O, Supporting Information). A_2_R is coupled to stimulatory G_s_ protein and triggers cAMP/PKA (protein kinase A)‐mediated signaling. We found that PKA signaling‐related molecules (such as PKA, p‐ERK, and p‐CREB) were unaltered in the hippocampus of *Bmal1*‐iKO mice (Figure S4P, Supporting Information). Therefore, impaired LTP and cognitive memory were associated with reduced adenosine‐A_1_R signaling. We further asked whether adenosine receptors have a role in the down‐regulation of adenosine‐A_1_R signaling. We found no changes in hippocampal expression of A_1_R (and A_2_R) in *Bmal1*‐iKO mice (Figure 3E). Therefore, the alteration in adenosine signaling was due to reduced adenosine levels but not reductions in the expression of adenosine receptors. It was noteworthy that neither intestinal nor blood adenosine varied according to the time‐of‐day (Figure 3C,D). However, hippocampal adenosine displayed 24 h oscillations (with a peak at the dark: light transition) in control mice, which was in sync with the daily rhythm in cognition (Figure 3D; Table S1, Supporting Information). While hippocampal adenosine was reduced in *Bmal1*‐iKO mice, its diurnal rhythmicity was retained (Figure 3D; Table S1, Supporting Information). This was in agreement with the observation that diurnal cognition rhythm persisted in *Bmal1*‐iKO mice (Figure S1I–L and Table S1, Supporting Information). Overall, the temporal data supported the involvement of adenosine in intestinal *Bmal1* gating of cognition.

Microglial activation and ensuing neuroinflammation have been shown to participate in delirium‐associated cognitive impairment.^[^ ^20^ ^]^ However, microglial activation had no or negligible contribution to the regulation of cognition by the intestinal clock because the number and morphology of Iba1^+^ cells as well as the levels of inflammatory factors (such as *Il‐1β, Tnfα*, and *Ccl8*) in the hippocampus and in primary microglial cells were not different between *Bmal1*‐iKO and control mice (Figure S5A–E, Supporting Information). Prior studies also suggest that the down‐regulation of polyunsaturated fatty acids (PUFAs, e.g., docosahexaenoic acid) in the hippocampus may contribute to a cognitive deficit.^[^ ^24^, ^45^ ^]^ However, we did not observe any changes in hippocampal PUFAs between *Bmal1*‐iKO and control mice (Figure S5F, Supporting Information), precluding a possibility that the intestinal clock regulates cognition through PUFAs. McCauley et al identified corticosterone (a glucocorticoid) as a key contributor to diurnal changes in synaptic plasticity and in hippocampus‐dependent behaviors.^[^ ^16^ ^]^ However, our metabolomics profiling of *Bmal1*‐iKO versus control mice did not reveal a change in corticosterone in the small intestine (Figure S5G,H, Supporting Information).^[^ ^24^ ^]^ *Bmal1*‐iKO did not cause changes in circulating and hippocampal levels of corticosterone either based on LC‐MS/MS with multiple reaction monitoring (Figure S5H, Supporting Information). Therefore, we may exclude the role of corticosterone in driving *Bmal1*‐iKO‐induced perturbance in cognitive behaviors.

### 2.4 Enhancement of Adenosine‐A_1_R Signaling rescues *Bmal1*‐iKO‐Induced Cognitive Impairments

**Figure 4:**
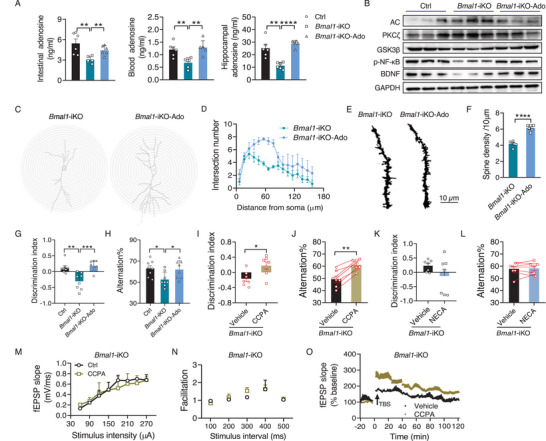
Adenosine‐A_1_R signaling enhancement rescues *Bmal1*‐iKO‐induced cognitive impairments. A) Adenosine levels in the small intestine, blood, and hippocampus from *Bmal1*‐iKO mice gavaged with adenosine or vehicle (*n* = 6). B) Immunoblotting of hippocampal enzymes involved in adenosine‐A_1_R signaling in *Bmal1*‐iKO mice gavaged with adenosine or vehicle. Three of nine samples from nine mice were pooled to generate three biological replicates. C) Representative Golgi‐stained CA1 pyramidal neurons, showing increased total dendrite length in *Bmal1*‐iKO mice gavaged with adenosine. D) Neurite arborization of CA1 pyramidal neurons in *Bmal1*‐iKO mice gavaged with adenosine or vehicle (*n* = 6). E) Representative images of dendritic branches from Golgi‐stained CA1 pyramidal neurons (scale bar, 10 µm). F) Spine density of the dendrites of CA1 pyramidal neurons in *Bmal1*‐iKO mice gavaged with adenosine or vehicle (*n* = 6). G) Discrimination index in NOR test during 2 h retention session in *Bmal1*‐iKO mice gavaged with adenosine or vehicle (*n* = 7). H) Spontaneous alternations in Y maze test in *Bmal1*‐iKO mice gavaged with adenosine or vehicle (*n* = 8). I) Discrimination index in NOR test during 2 h retention session in *Bmal1*‐iKO mice treated with CCPA or vehicle (*n* = 7). J) Spontaneous alternations in Y maze test in *Bmal1*‐iKO mice treated with CCPA or vehicle (*n* = 7). K) Discrimination index in NOR test during 2 h retention session in *Bmal1*‐iKO mice treated with NECA or vehicle (*n* = 7). L) Spontaneous alternations in Y maze test in *Bmal1*‐iKO mice treated with NECA or vehicle (*n* = 7). M) Input/output curves in hippocampal slices from *Bmal1*‐iKO mice treated with CCPA (*n* = 4) or vehicle (*n* = 3). N) Paired‐pulse facilitation in hippocampal slices from *Bmal1*‐iKO mice treated with CCPA (*n* = 4) or vehicle (*n* = 3). O) Time course of fEPSP recordings in hippocampal slices from *Bmal1*‐iKO mice treated with CCPA (*n* = 4) or vehicle (*n* = 3). For panels (J,L), the pairing of datapoints represent the same mice tested first vehicle and then CCPA or NECA. Discrimination indices were calculated as: (Time for novel object exploring – time for familiar object exploring)/(Time for novel object exploring + time for familiar object exploring). All behavioral tests were conducted at ZT6. Mice were sacrificed and the samples were collected at ZT6. Data are mean ± SEM, and analyzed by two‐way ANOVA followed by Bonferroni posttest (A,G, and H) and two‐tailed Student's t‐test (F,I, and J). *p  <  0.05, ^**^ *p*  <  0.01, ^***^ *p* < 0.001 and ^****^ *p* < 0.0001.

To assess whether dietary adenosine can reach the brain tissues, we gavaged wild‐type mice with [^3^H]‐adenosine, and measured the [^3^H]‐adenosine radioactivity in the blood and hippocampus. After administration, [^3^H]‐adenosine rapidly appeared in the blood circulation and the highest concentration was observed at 15 min post‐dosing (Figure S6A, Supporting Information). We also found a significant amount of [^3^H]‐adenosine in mouse hippocampus 90 min after [^3^H]‐adenosine gavage, accounting for 0.06% of dosed [^3^H]‐adenosine (Figure S6A, Supporting Information). Given that cognitive impairment in *Bmal1*‐iKO mice is attributed to a reduced adenosine tone and compromised hippocampal adenosine signaling, we wondered whether enhancement of adenosine signaling by adenosine supplementation can rescue the cognitive deficits in the knockouts. To test this, *Bmal1*‐iKO mice were gavaged with adenosine (5 mg kg^−1^) for 21 days that allowed adenosine to recover to normal levels in the small intestine, blood, and hippocampus (**Figure** **4**A; Figure S6B, Supporting Information). This was accompanied by enhanced adenosine signaling as AC and PKC expression were decreased and GSK3β, p‐NF‐κB and BDNF levels were increased in the hippocampus of the knockouts (Figure 4B; Figure S6C,D, Supporting Information). Enhancement of adenosine signaling was also evidenced by prolonged dendrite length and increased dendritic spine density in *Bmal1*‐iKO mice (Figure 4C,F; Figure S6E–H, Supporting Information). Concurrently, *Bmal1*‐iKO mice treated with adenosine showed a normal novel object preference and had an increased rate of spontaneous alterations (Figure 4G,H; Figure S6I,J, Supporting Information), indicative of cognitive improvement. Likewise, a chow diet supplemented with 0.1% adenosine rescued the cognitive deficits in *Bmal1*‐iKO mice (Figure S6K,L, Supporting Information). We further tested whether enhancement of adenosine signaling by an A_1_R agonist can attenuate *Bmal1*‐iKO‐induced cognitive impairments. CCPA (2‐chloro‐N6‐cyclopentyladenosine, a specific agonist of A_1_R) treatment (0.05 mg kg^−1^, i.p.) ameliorated cognitive deficits with respect to memory in *Bmal1*‐iKO mice (Figure 4I,J; Figure S7A–D, Supporting Information). Conversely, cognitive deficits were aggravated in *Bmal1*‐iKO mice upon treatment with DPCPX, a specific antagonist of A_1_R (Figure S7E,F, Supporting Information). Consistent with the administration route of intraperitoneal injection, direct application of CCPA in the hippocampus (by a mini‐osmotic pump) ameliorated, whereas DPCPX treatment aggravated, the cognitive impairments in *Bmal1*‐iKO mice (Figure S7G–J, Supporting Information). In sharp contrast, NECA (5′‐N‐ethylcarboxamidoadenosine, an A_2_R agonist, 0.08 mg kg^−1^, i.p.), CGS21680 (a specific A_2A_R agonist, 0.1 mg kg^−1^, i.p.) and SCH58261 (a specific A_2A_R antagonist, 0.1 mg kg^−1^, i.p.) had no effects on *Bmal1*‐iKO‐induced cognitive decline (Figure 4K,L; Figure S7K–P, Supporting Information). In line with its protective effects on cognitive deterioration, CCPA enhanced the hippocampal LTP in *Bmal1*‐iKO mice but did not affect basal synaptic transmission and presynaptic function (Figure 4M,O; Figure S7Q–T, Supporting Information). In addition, AAV_2/9_ vector encoding *Adora1* (AAV‐A_1_R) was stereotaxically injected into the hippocampus of *Bmal1*‐iKO mice to up‐regulate A_1_R expression (Figure S7U, Supporting Information). We observed abrogation of cognitive decline by AAV‐A_1_R in *Bmal1*‐iKO mice (Figure S7V,W, Supporting Information). On the other hand, silencing hippocampal A_1_R (by injecting recombinant AAV_2/9_ expressing *Adora1* siRNA) in wild‐type mice replicated the cognitive phenotype of *Bmal1*‐iKO mice (Figure S7U,X,Y, Supporting Information). Consistently, DPCPX attenuated the hippocampal LTP in wild‐type mice (Figure S7Z, Supporting Information). Altogether, these findings supported that hippocampal adenosine‐A_1_R signaling is involved in the regulation of cognitive function by the intestinal clock.

### 2.5 Intestinal *Bmal1* Regulates Adenosine Absorption Through Adenosine Kinase (ADK)

**Figure 5:**
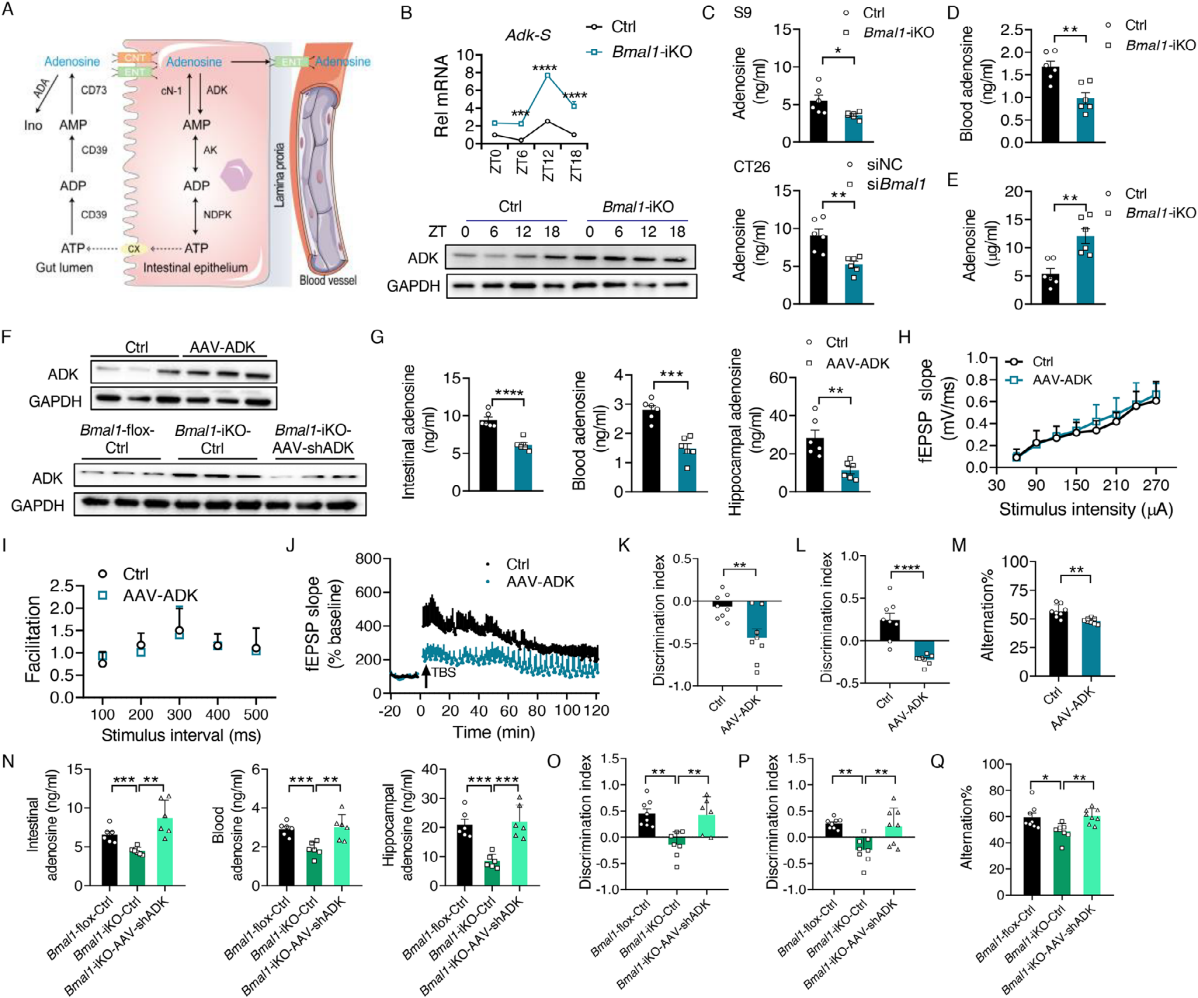
Intestinal *Bmal1* regulates adenosine absorption via adenosine kinase (ADK). A) A diagram for intestinal disposition and absorption of adenosine. B) Relative expression of intestinal *Adk‐S* mRNA and ADK protein in *Bmal1*‐iKO and control mice (*n* = 6). For western blotting analysis of diurnal protein expression, three of nine samples from nine mice at each time point were pooled to generate three biological replicates. One representative blot is shown from three biological replicates. C) ADK activities were measured using intestinal S9 fraction (prepared from *Bmal1*‐iKO and *Bmal1‐*flox mice) and siBmal1‐ or siNC‐treated CT26 cells (*n* = 6). D) Adenosine levels in the portal vein blood from *Bmal1*‐iKO and control mice (*n* = 6). E) Adenosine levels in the intestinal content plus feces from *Bmal1*‐iKO and control mice (*n* = 6). F) Immunoblotting of ADK in the small intestine from mice after injection of AAV‐ADK or AAV‐shADK or control. Three of nine samples from nine mice were pooled to generate three biological replicates. G) Adenosine levels in the small intestine, blood, and hippocampus from wild‐type mice after injection of AAV‐ADK and control virus (*n* = 6). H) Input/output curves in hippocampal slices from wild‐type mice after injection of AAV‐ADK and control virus (*n* = 4). I) Paired‐pulse facilitation in hippocampal slices from wild‐type mice after injection of AAV‐ADK and control virus (*n* = 4). J) Time course of fEPSP recordings in hippocampal slices from wild‐type mice after injection of AAV‐ADK and control virus (*n* = 4). K) Discrimination index in NOR test during 2 h retention session in wild‐type mice after injection of AAV‐ADK and control virus (*n* = 8). L) Discrimination index in NOR test during 24 h recall session in wild‐type mice after injection of AAV‐ADK and control virus (*n* = 8). M) Spontaneous alternations in Y maze test in wild‐type mice after injection of AAV‐ADK and control virus (*n* = 8). N) Adenosine levels in the small intestine, blood, and hippocampus from *Bmal1*‐iKO mice after injection of AAV‐shADK and control virus (*n* = 6). O) Discrimination index in NOR test during 2 h retention session in *Bmal1*‐iKO mice after injection of AAV‐shADK and control virus (*n* = 6–8). P) Discrimination index in NOR test during 24 h recall session in *Bmal1*‐iKO mice after injection of AAV‐shADK and control virus (*n* = 8). Q) Spontaneous alternations in Y maze test in *Bmal1*‐iKO mice after injection of AAV‐shADK and control virus (*n* = 8). Discrimination indices were calculated as: (Time for novel object exploring – time for familiar object exploring)/(Time for novel object exploring + time for familiar object exploring). For data in panels (K–M and O–Q), all behavioral tests were conducted at ZT6. For data in panels (G–J and N), the samples were collected at ZT6. Data are mean ± SEM, and analyzed by two‐way ANOVA followed by Bonferroni posttest (B and N–Q) and two‐tailed Student's *t*‐test (C–E, G, and K–M). ^*^ *p*  <  0.05, ^**^ *p*  <  0.01, ^***^ *p* < 0.001 and ^****^ *p* < 0.0001. ADA, adenosine deaminase; AK1, adenylate kinase‐1; CNT, concentrative nucleoside transporter; ENT, equilibrative nucleoside transporter; NDPK, nucleotide diphosphokinase; NC, negative control.

As is noted, adenosine levels in the small intestine, blood, and hippocampus were consistently lowered in *Bmal1‐*iKO mice. We thus asked whether this was due to a change in intestinal absorption of adenosine. Intestinal disposition and absorption of adenosine are rather complex processes (**Figure** **5**A).^[^ ^46^ ^]^ In the intestinal lumen, adenosine can be metabolized to inosine by adenosine deaminase (ADA) or transported into enterocytes by equilibrative nucleoside transporter (ENT) and concentrative nucleoside transporter (CNT). Within the enterocytes, adenosine can be converted to ATP using multiple metabolic enzymes (i.e., ADK, adenylate kinase 1 [AK1], and nucleotide diphosphokinase [NDPK]) and this conversion is reversible (Figure 5A). Alternatively, adenosine in the enterocytes can be transported into the blood by ENT for absorption (Figure 5A). To explore whether adenosine absorption is reduced in *Bmal1‐*iKO mice, we first examined the expression of intestinal adenosine‐processing genes (such as *Adk, Ak1, Ada, Nme2, Cnt1/2/3, Ent1/2/3/4, Cd39*, and *Cd73*) in the knockouts. We found that all adenosine‐processing genes except *Adk* (which had significant expression in the small intestine, colon, liver, and brain) were unaffected in the small intestine of *Bmal1‐*iKO mice (Figure 5B; Figure S8A–C, Supporting Information). Intestinal *Adk‐S* (the short isoform) but not *Adk‐L* (the long isoform) was up‐regulated across a 24 h cycle in the knockouts with striking up‐regulation at ZT12‐18 (Figure 5B; Figure S8B, Supporting Information). Consistently, *Bmal1‐*iKO led to the up‐regulation of intestinal ADK protein (Figure 5B; Figure S8A, Supporting Information). Cell‐ and Figure S9 (Supporting Information) fraction‐based in vitro metabolism assays confirmed that ADK activity was increased in the small intestine of *Bmal1‐*iKO mice (Figure 5C). In accordance with ADK up‐regulation, the adenosine level in the portal vein blood was lower in the knockouts as compared to the controls (Figure 5D). Notably, the adenosine levels in the intestinal content plus feces were higher in *Bmal1‐*iKO mice than in controls (Figure 5E). Therefore, intestinal absorption of adenosine was reduced in *Bmal1‐*iKO mice probably due to up‐regulation of ADK. Tight associations of the adenosine level with ADK expression have been well‐established in the literature.^[^ ^47^ ^]^ In a prior report, up‐regulation of ADK by over‐expressing an *Adk* transgene in mice leads to reduced levels of adenosine.^[^ ^47^ ^]^ Indeed, the impact of ADK expression on cognition has been revealed in animal models, with transgenic mice overexpressing ADK showing defects in learning and memory.^[^ ^48^ ^]^ Additionally, we examined the expression of adenosine‐processing genes and clock genes in the liver and hippocampus of *Bmal1‐*iKO mice, and found that none of these genes were altered (Figure S8D–H, Supporting Information).^[^ ^24^ ^]^ Thus, the lower levels of adenosine in the blood and hippocampus were due to reduced adenosine absorption in the small intestine.

To verify the role of intestinal ADK in BMAL1 regulation of adenosine levels and cognitive function, we performed ectopic expression of ADK in wild‐type mice specifically in the intestine using AAV_9_ expressing *Adk‐S* under the control of *Villin* promoter (AAV_9_‐Villin‐ADK‐eGFP, hereafter referred to as AAV_9_‐ADK) (Figure 5F; Figure S9A,B,D, Supporting Information). Exogenous ADK significantly decreased the intestinal, blood, and hippocampal levels of adenosine (Figure 5G). Concurrently, hippocampal LTP, but not basal synaptic transmission and presynaptic function, was impaired in AAV_9_‐ADK‐treated mice (Figure 5H,J). As a result, these mice showed compromised cognitive memory (Figure 5K,M). On the other hand, we specifically silenced the *Adk* gene in the intestine of *Bmal1*‐iKO mice using AAV_9_ expressing shRNA targeting *Adk‐S* (Figure 5F; Figure S9A,C,D, Supporting Information). Intestinal ADK at ZT18 can be controlled to be the level of endogenous ADK in control mice by adjusting the virus dose (Figure 5F; Figure S9A, Supporting Information). Recovery of intestinal ADK led to improvement of cognition in *Bmal1*‐iKO mice owning to normalization of intestinal and systemic adenosine levels (Figure 5N,Q; Figure S9E–H, Supporting Information). Because adenosine absorption is regulated by BMAL1 whose expression oscillates as a function of time‐of‐day in the intestine,^[^ ^24^ ^]^ it is of interest to examine potential time‐of‐day effects on cognition of adenosine supplementation using 5 × FAD mouse model of Alzheimer's disease (AD). We found that adenosine dosing time‐dependently improved cognitive performance in mice, and the strongest effect was observed when the chemical was dosed at ZT6, corresponding to peak expression of intestinal BMAL1 and most extensive absorption of adenosine (Figure S10A–D, Supporting Information). Collectedly, our findings revealed that intestinal *Bmal1* regulates the absorption of and hippocampal level of adenosine through ADK, a primary enzyme for clearance of adenosine.

### 2.6 Regulation of ADK by Intestinal *Bmal1* Requires REV‐ERBα

**Figure 6:**
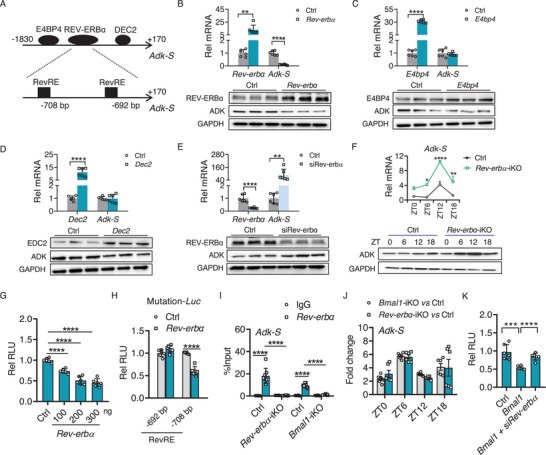
Intestinal *Bmal1* negatively regulates ADK through REV‐ERBɑ. A) Schematic presentation of the transcription factor binding sites in the *Adk‐S* promoter obtained from the JASPAR database. B) Effects of *Rev‐erbɑ* overexpression on ADK expression (*n* = 6). C) Effects of *E4bp4* overexpression on ADK expression (*n* = 6). D) Effects of *Dec2* overexpression on ADK expression (*n* = 6). E) Effects of *Rev‐erbɑ* knockdown on ADK expression (*n* = 6). F) Relative expression of intestinal *Adk‐S* mRNA and ADK protein in *Rev‐erbɑ*‐iKO and control mice (*n* = 6). For western blotting analysis of diurnal protein expression, three of nine samples from nine mice at each time point were pooled to generate three biological replicates. One representative blot is shown from three biological replicates. G) Luciferase reporter assays showing dose‐dependent inhibition of *Adk‐S* transcription by *Rev‐erbɑ* (*n* = 6). H) Effects of RevRE site mutation on REV‐ERBɑ regulation of *Adk‐S* transcription (*n* = 6). I) ChIP assays showing recruitment of intestinal REV‐ERBɑ protein to *Adk‐S* promoter in mice (*n* = 8). J) Comparisons of *Adk‐S* mRNA levels in *Bmal1*‐iKO or *Rev‐erbɑ*‐iKO mice and controls at different diurnal time points (*n* = 6). K) Effects of *Bmal1* on *Adk‐S* transcription in siRev‐erbɑ‐treated (*Rev‐erbɑ‐*deficient) cells (*n* = 6). Data are mean ± SEM, and analyzed by two‐tailed Student's t‐test (B–E, H, and K), one‐way ANOVA followed by Bonferroni post‐test (G), and two‐way ANOVA followed by Bonferroni posttest (F,I). ^*^ *p*  <  0.05, ^**^ *p*  <  0.01, ^***^ *p* < 0.001 and ^****^ *p* < 0.0001.

Next, we investigated the molecular mechanism by which BMAL1 regulates ADK expression in the small intestine. Given that BMAL1 (a positive regulator of gene transcription and expression) showed a negative regulatory effect on intestinal *Adk‐S* (Figure S11A,B, Supporting Information), an intermediate regulator was necessary to mediate the action of BMAL1. Such a mediator should be a target of BMAL1 and a negative regulator of *Adk‐S*. Bioinformatic analysis with the JASPAR database predicted the binding sites in the *Adk‐S* promoter for the transcription factors such as REV‐ERBα (a known BMAL1 target), E4BP4, and DEC2 (a known BMAL1 target) (**Figure** **6**A). We thus tested whether these three transcription factors regulate *Adk‐S* expression by performing cell cotransfection assays. Overexpression of *Rev‐erbɑ* but not *E4bp4* or *Dec2* led to decreases in ADK mRNA and protein expression in CT26 cells (Figure 6B,D; Figure S11C, Supporting Information). In support of negative regulation of ADK by REV‐ERBα, the knockdown of *Rev‐erbɑ* (by a specific siRNA) caused an increase in the cellular expression of ADK (Figure 6E; Figure S11D, Supporting Information). To interrogate whether intestinal REV‐ERBα regulates *Adk‐S* in vivo, we generated mice with *Rev‐erbα* specifically knocked out in the intestine (named *Rev‐erbα*‐iKO mice) as described in our previous work,^[^ ^27^ ^]^ and examined the intestinal expression of ADK in this mouse line. *Rev‐erbα*‐iKO mice showed an increase in *Adk‐S* but not *Adk‐L* mRNA expression across the entire 24 h cycle, but no changes in other adenosine‐processing genes (Figure 6F; Figure S11E, Supporting Information). In keeping with the mRNA change, intestinal ADK protein was increased in *Rev‐erbα*‐iKO mice (Figure 6F; Figure S11E, Supporting Information). These findings supported that REV‐ERBɑ negatively regulates ADK expression, and suggested a role of intestinal REV‐ERBα in mediating BMAL1 regulation of ADK.

Parallel changes in *Adk‐S* mRNA and its protein suggested a transcriptional mechanism for REV‐ERBɑ regulation of ADK. Sequence analysis identified two RevRE (a binding motif for REV‐ERBɑ) elements in the promoter of *Adk‐S* (Figure 6A). *Adk‐L* and *Adk‐S* are driven by their own promoters, named P1 and P2, respectively. Co‐transfection of a *Rev‐erbɑ*‐encoding plasmid with a luciferase reporter driven by *Adk* P2 promoter led to a dose‐dependent decrease in the reporter activity, indicating transcriptional regulation of *Adk‐S* by REV‐ERBɑ (Figure 6G). However, *Rev‐erbɑ* overexpression had no effects on the activity of a luciferase reporter driven by *Adk* P1 promoter (Figure S11F, Supporting Information). Mutation assays revealed that ‐722/‐708 bp (a RevRE element) region in the *Adk* P2 promoter was indispensable for the REV‐ERBɑ action (Figure 6H). Furthermore, ChIP assays confirmed direct binding of intestinal REV‐ERBɑ to *Adk* P2 promoter, which was reduced in the small intestine of both *Rev‐erbα*‐iKO and *Bmal1*‐iKO mice (Figure 6I). Therefore, REV‐ERBɑ binds to the *Adk* P2 promoter and inhibits its transcription and expression in the small intestine. We next examined whether REV‐ERBɑ is indeed required for BMAL1 regulation of ADK expression. We quantified *Adk‐S* transcripts in the small intestine from *Rev‐erbɑ‐*iKO mice and controls. Intestinal *Adk‐S* expression was increased to the *Bmal1*‐iKO levels in *Rev‐erbɑ‐*iKO mice (Figure 5B,F,J). Additionally, the knockdown of *Rev‐erbɑ* markedly attenuated the inhibitory effects of BMAL1 on *Adk‐S* expression in CT26 cells (Figure 6K). These findings indicate that intestinal BMAL1 regulation of *Adk‐S* requires REV‐ERBɑ.

**Figure 7:**
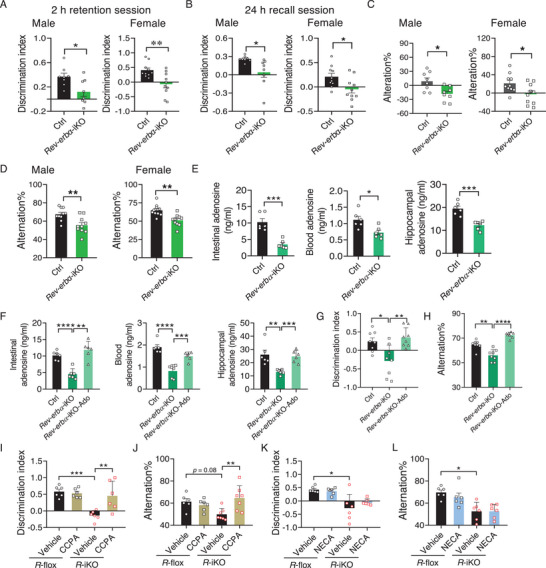
Intestinal *Rev‐erb*α promotes adenosine absorption and cognitive performance. A) Discrimination index in NOR test during 2 h retention session in *Rev‐erbα*‐iKO and control mice (*n* = 8–10). B) Discrimination index in NOR during 24 h recall session in *Rev‐erbα*‐iKO and control mice (*n* = 8–10). C) Performance on SOL test in *Rev‐erbα*‐iKO and control mice (*n* = 8–10). D) Spontaneous alternations in Y maze test in *Rev‐erbα*‐iKO and control mice (*n* = 8–10). E) Adenosine levels in the small intestine, blood, and hippocampus from *Rev‐erbα*‐iKO and control mice (*n* = 6). F) Adenosine levels in the small intestine, blood, and hippocampus from *Rev‐erbα*‐iKO mice gavaged with adenosine or vehicle (*n* = 6). G) Discrimination index in NOR test during 2 h retention session in *Rev‐erbα*‐iKO mice gavaged with adenosine or vehicle (*n* = 8). H) Spontaneous alternations in Y maze test in *Rev‐erbα*‐iKO mice gavaged with adenosine or vehicle (*n* = 8). I) Discrimination index in NOR test during 2 h retention session in *Rev‐erbα*‐iKO and control mice treated with CCPA or vehicle (*n* = 6). J) Spontaneous alternations in Y maze test in *Rev‐erbα*‐iKO and control mice treated with CCPA or vehicle (*n* = 6). K) Discrimination index in NOR test in *Rev‐erbα*‐iKO and control mice treated with NECA or vehicle (*n* = 6). L) Spontaneous alternations in Y maze test in *Rev‐erbα*‐iKO and control mice treated with NECA or vehicle (*n* = 6). Discrimination indices were calculated as: (Time for novel object exploring – time for familiar object exploring)/(Time for novel object exploring + time for familiar object exploring). All behavioral tests were conducted at ZT6, and the analyzed samples were collected at ZT6. Data are mean ± SEM, and analyzed by two‐tailed Student's *t*‐test (A–E) and two‐way ANOVA followed by Bonferroni posttest (F–L). ^*^ *p*  <  0.05, ^**^ *p*  <  0.01, ^***^ *p* < 0.001 and ^****^ *p* < 0.0001. R‐flox, *Rev‐erbα*‐flox; R‐iKO, *Rev‐erbα*‐iKO.

Given that REV‐ERBɑ acts as a mediator for the repression of ADK by intestinal *Bmal1*, it was reasonable to postulate that intestinal *Rev‐erbα* would promote adenosine absorption and cognitive performance. To test this, we examined the cognitive behaviors and adenosine levels in *Rev‐erbα*‐iKO mice. We observed cognitive deficits and reduced adenosine levels (in the small intestine, blood, and hippocampus) in *Rev‐erbα*‐iKO mice (**Figure** **7**A,E). The cognitive deficits in these knockouts were rescued by adenosine supplementation through either direct gavage or diet feeding (Figure 7F,H; Figures S6K,L and S11G–I, Supporting Information). Moreover, CCPA treatment ameliorated the cognitive deficits in *Rev‐erbα*‐iKO mice, whereas NECA treatment had no effects (Figure 7I,L). Altogether, these findings validated the promoting effects of intestinal *Rev‐erbα* on adenosine absorption and cognitive behaviors, and evidenced a mediating role of REV‐ERBɑ in regulation of cognition by intestinal BMAL1.

### 2.7 Intestinal Clock Gating of Cognition is Independent of the Liver

**Figure 8:**
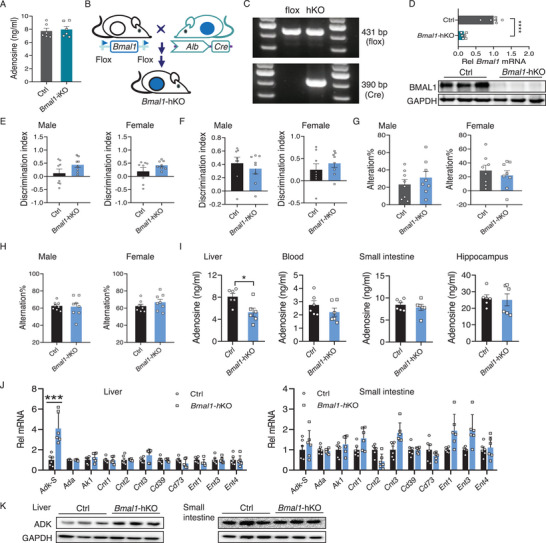
Liver clock does not affect cognitive function. A) Adenosine levels in the liver from *Bmal1*‐iKO and control mice (*n* = 6). B) Schematic illustration of the generation of hepatocyte‐specific *Bmal1* knockout (*Bmal1*‐hKO) mice. C) PCR genotyping using mouse tails from *Bmal1*‐hKO and control (*Bmal1*‐flox) mice. D) Relative expression of hepatic *Bmal1* mRNA and protein in *Bmal1*‐hKO and control mice. E) Discrimination index in NOR test during 2 h retention session in *Bmal1*‐hKO and control mice (*n* = 8). F) Discrimination index in NOR during 24 h recall session in *Bmal1*‐hKO and control mice (*n* = 8). G) Performance on SOL test in male *Bmal1*‐hKO and control mice (*n* = 8). H) Spontaneous alternations in Y maze test in male *Bmal1*‐hKO and control mice (*n* = 8). I) Adenosine levels in the liver, blood, small intestine, and hippocampus from male *Bmal1*‐hKO and control mice (*n* = 6). J) Relative expression of adenosine‐processing genes in the liver and small intestine of male *Bmal1*‐hKO and control mice (*n* = 6). K) Relative expression of ADK protein in the liver and small intestine of male *Bmal1*‐hKO and control mice. Three of nine samples from nine mice were pooled to generate three biological replicates. Discrimination indices were calculated as: (Time for novel object exploring – time for familiar object exploring)/(Time for novel object exploring + time for familiar object exploring). All behavioral tests were conducted at ZT6, and the analyzed samples were collected at ZT6. All Data are mean ± SEM, and analyzed by two‐tailed Student's *t*‐test (I,J). ^*^ *p*  <  0.05, ^**^ *p*  <  0.01, and ^***^ *p* < 0.001.

Because the intestinal clock is previously shown to modulate liver metabolism and peripheral rhythmicity,^[^ ^24^ ^]^we wondered whether liver metabolism has a role in regulating cognition by the intestinal clock. As described (Figure S8D, Supporting Information), we found that adenosine‐processing genes (such as *Adk, Ada, Cd39, Cd73, Cnt2*, and *Ent1/2/3*) in the liver were unaffected in *Bmal1*‐iKO mice. Moreover, Figure S9 (Supporting Information) fraction‐based in vitro metabolism assays confirmed that adenosine metabolism remained unaltered in the liver of *Bmal1‐*iKO mice (**Figure** **8**A). Therefore, we can exclude the role of the liver in the regulation of adenosine levels and cognition by the intestinal clock.

Given that the intestinal clock regulates cognition through enhancing adenosine tone and promoting adenosine‐A_1_R signaling, we asked whether the clock in the liver (as a major metabolic organ) has a similar regulatory function. To this end, we generated hepatocyte‐specific *Bmal1* knockout mice (named *Bmal1*‐hKO mice) by breeding *Bmal1*‐flox mice with mice expressing *Alb*‐Cre and tested their cognitive behaviors and memory (Figure 8B,D; Figure S12A, Supporting Information). Both male and female *Bmal1*‐hKO mice had normal short‐term and long‐term memory according to NOR tests (Figure 8E,F). Also, these mice had a normal spatial memory as evidenced by preferential exploration of the novel location in the SOL test and normal spontaneous alternations in the Y maze test (Figure 8G,H). In addition, *Bmal1*‐hKO did not alter the diurnal variations in cognitive memory (Figure S12B,C and Table S1, Supporting Information). Thus, hepatocyte‐specific ablation of *Bmal1*, in effect clock function, did not affect cognitive memory. We further examined the levels of adenosine in the liver, blood, small intestine, and hippocampus. We found that hepatic adenosine was reduced in *Bmal1*‐hKO mice across 24 h cycle, however, the adenosine in blood, small intestine, and hippocampus remained unchanged (Figure 8I; Figure S12D,E, Supporting Information). A screen of hepatic adenosine‐processing genes (including *Ada, Adk‐S, Ak1, Cnt1/2/3, Ent1/2/3/4, Cd39*, and *Cd73*) revealed that only *Adk‐S* was altered (up‐regulated) in *Bmal1*‐hKO mice (Figure 8J). In keeping with the mRNA up‐regulation, ADK protein was increased in the liver upon *Bmal1*‐hKO (Figure 8K; Figure S12F, Supporting Information). Thus, the repressive action of BMAL1 on *Adk‐S* transcription and expression can be extended from the small intestine to the liver (Figure 5B). Unsurprisingly, intestinal *Adk‐S* and other adenosine‐processing genes were not affected by *Bmal1*‐hKO (Figure 8J,K). The reduction in hepatic adenosine caused by *Bmal1*‐hKO was most likely a result of the up‐regulation of ADK as these causal links were already established in the small intestine (Figure 5). However, it was noted that the alteration in hepatic metabolism of adenosine in *Bmal1*‐hKO mice did not translate to a change in the circulating level of adenosine. This was probably because intestinal metabolism has a much greater effect on adenosine bioavailability than hepatic metabolism as noted for certain drugs such as raloxifene,^[^ ^49^ ^]^ although the exact reason is unknown. Taken together, the intestinal clock modulates cognitive memory independent of the liver. Our findings highlighted the indispensable role of the intestinal clock in determining nutrient metabolism and modulating the functions of extra‐intestinal tissues.

## 3 Discussion

In the present study, we have demonstrated that the intestinal clock regulates cognitive capacity, underscoring the role of the intestinal clock in maintaining brain health and illustrating that the functionality of the central nervous system can be modulated by a peripheral clock.^[^ ^50^ ^]^ Along this line, the adipocyte clock modulates the susceptibility of mice to obesity by regulating polyunsaturated fatty acids in hypothalamic neurons and the diurnal rhythm of food intake.^[^ ^51^ ^]^ The intestinal clock and gut microbiome are known to interact and bidirectional regulation exists,^[^ ^52^ ^]^ raising a possibility for the involvement of the microbiome in the functioning of the intestinal clock. We found that the regulatory effects of the intestinal clock on cognitive performance are independent of gut microbiome as depletion of microbiota (through germ‐free approach or ABX treatment) does not affect the alterations in intestinal adenosine absorption and in cognitive behaviors in *Bmal1*‐iKO mice (Figure S1C–H, Supporting Information). However, we observe deficits in cognitive behaviors in microbiota‐ablated wild‐type mice as compared to control (conventionally raised SPF) mice, consistent with a prior report.^[^ ^29^ ^]^ The association of gut microbiota with cognitive function is also established in humans.^[^ ^53^ ^]^ Although the mechanisms underlying the regulation of cognition by gut microbes remain elusive, there is growing evidence for the involvement of short‐chain fatty acids.^[^ ^54^, ^55^ ^]^ Short‐chain fatty acids generated by gut microbes might modulate cognitive function via interactions with G protein‐coupled receptors and/or histone deacetylases and exert their effects through immune, endocrine, and humoral pathways.^[^ ^56^ ^]^ Gut‐derived hormones (such as cholecystokinin and glucagon‐like peptide‐1) have been also shown to modulate brain functions such as cognitive function, playing a role in the communications between the gut and brain (also known as the gut‐brain axis)*^.^* ^[^ ^57^, ^58^ ^]^ Here gut‐processed adenosine is identified as a key contributor to maintenance of cognitive capacity. Therefore, this study has conceptually expanded the gut‐brain axis by incorporating the intestinal clock and adenosine signaling.

It is noteworthy that the cognitive performance (assessed by novel object recognition and Y maze tests) in normal mice displays a diurnal rhythm that peaks at the dark: light transition (Figure S1I,J; Table S1, Supporting Information). We may ascribe this diurnal pattern to the temporal oscillations in hippocampal adenosine level and adenosine signaling, which are positively correlated with synaptic plasticity and cognitive capacity under a physiological condition.^[^ ^37^ ^]^ In line with the diurnal pattern of cognition, hippocampal LTP is more robust at ZT0 (the dark: light transition) than at ZT12 (the light: dark transition) (Figure 2J; Table S1, Supporting Information).^[^ ^16^ ^]^ While cognitive performance is impaired upon intestinal clock deficiency, the cognitive rhythmicity retains (Figure S1I–L; Table S1, Supporting Information). Along this line, hippocampal adenosine is lowered around the clock but its rhythmicity persists when the intestinal BMAL1 is functionally deficient (Figure 3C,D). Apparently, the intestinal clock regulates total brain adenosine level and cognitive capacity, but does not contribute much to their daily oscillations. The daily rhythm of brain adenosine is potentially accounted for by diurnal neuronal activity and prolonged wakefulness.^[^ ^59^ ^]^

We have shown that adenosine‐A_1_R signaling plays a pivotal role in the modulation of cognition by an intestinal clock. Supporting the role of adenosine in regulating cognition, the daily rhythm of cognitive capacity is in sync with the rhythm of hippocampal adenosine in normal wild‐type mice (Figure 3D; Figure S1I–L and Table S1, Supporting Information). A prior study identifies corticosterone (a glucocorticoid) as a key contributor to diurnal changes in synaptic plasticity and in hippocampus‐dependent behaviors.^[^ ^16^ ^]^ This was mainly evidenced by the observation that chemical inhibition of the NR3C1/2 glucocorticoid receptors disrupts the daily rhythm in hippocampal LTP.^[^ ^16^ ^]^ Unfortunately, the molecular and cellular mechanisms underlying the corticosterone effects remain unknown. It is believed that corticosterone regulates circadian rhythms by enhancing cellular oscillatory capacity or by maintaining the entrainment of cellular clocks.^[^ ^60^ ^]^ Our metabolomics profiling of *Bmal1*‐iKO versus control mice did not reveal a change in corticosterone in the small intestine (Figure S5G, Supporting Information).^[^ ^24^ ^]^ *Bmal1*‐iKO did not cause changes in circulating and hippocampal levels of corticosterone either based on LC‐MS/MS with multiple reaction monitoring (Figure S5H, Supporting Information). Therefore, we may exclude the role of corticosterone in driving *Bmal1*‐iKO‐induced perturbance in cognitive behaviors. A variety of gut‐derived peptide hormones such as ghrelin, somatostatin, and cholecystokinin are shown to have a role in cognition and under circadian control.^[^ ^61^, ^62^ ^]^ There is a possibility that these peptide hormones besides adenosine contribute to the intestinal clock‐brain communications affecting cognition.

Our study lends strong support to the notion that hippocampal adenosine at a level below the normal range (~15.9–39.0 ng mL^−1^) has a detrimental effect on cognitive performance. It is noteworthy that abnormally high levels of adenosine in the brain are also associated with cognitive decline.^[^ ^63^, ^64^ ^]^ This is probably because adenosine at a high level acts primarily on the A_2_R (rather than A_1_R) receptor to activate the PKC signaling and to suppress BDNF expression and synaptogenesis, thereby impairing hippocampal LTP and memory.^[^ ^65^ ^]^ Therefore, we propose that hippocampal adenosine should be controlled to a fine range of ≈15.9–39.0 ng mL^−1^ for mental health. Jet‐lagged mice showed elevated levels (43.9–60.1 ng mL^−1^) of adenosine in the hippocampus, which was associated with cognitive decline (Figure S10E–G, Supporting Information). Altogether, our data support that both abnormally high and low levels of adenosine are detrimental to cognitive performance.

We have provided compelling evidence that dietary adenosine can reach the blood and the hippocampus. This appears to be in conflict with the notion that adenosine is rapidly metabolized in the intestine. It is noteworthy that the metabolism of adenosine to its nucleotides (such as AMP, ADP, and ATP) is reversible.^[^ ^46^, ^66^ ^]^ Thus, “metabolism” does not fully mean “elimination” in the case of adenosine considering that adenosine can be rapidly recycled from its nucleotide metabolites. In fact, it is generally accepted that luminal adenosine can be absorbed by intestinal epithelial cells through CNT and ENT transporters.^[^ ^67^ ^]^ Supporting this, dietary adenosine supplementation in gestating sows could increase its content in the placenta, and improve the placental angiogenesis.^[^ ^68^, ^69^ ^]^ Prior studies have also clearly demonstrated that adenosine can be transported across the blood‐brain barrier via uptake transporters (such as ENT1 in mice and CNT2 in rats).^[^ ^70^, ^71^, ^72^ ^]^ Although rapid metabolism also occurs in the brain microvasculature,^[^ ^73^ ^]^ a significant amount of adenosine can be bioavailable within the brain due to the recycling effect noted above. The established role of intestinal absorption in determining hippocampal adenosine levels provides a rationale for adenosine‐driven gut‐brain signaling.

Shift work and jet lag are two known physiological conditions that cause disruptions to the circadian clock. Reportedly, both shift work and jet lag are associated with impairments to cognition and memory.^[^ ^74^, ^75^ ^]^ Our finding that perturbance of the intestinal clock impairs hippocampal function and cognition via adenosine signaling provides an insight into why physiological clock disruption negatively impacts cognitive function. Notably, other factors such as disturbed sleep, and metabolic, hormonal, and inflammatory responses are also likely involved in cognitive impairments caused by physiological clock disruption.^[^ ^76^, ^77^, ^78^, ^79^ ^]^ Our findings also help explain the clinical observation that patients with inflammatory bowel diseases often suffer from cognitive deficits in addition to their classical symptoms.^[^ ^80^ ^]^ This is because intestinal inflammation is associated with local clock disruption in which *Bmal1* and *Rev‐erbɑ* are markedly down‐regulated.^[^ ^81^ ^]^ As demonstrated here, deficiency of intestinal *Bmal1* and *Rev‐erbɑ* results in reduced systemic adenosine and A_1_R signaling in the hippocampus, and in turn impaired cognitive function involving LTP and BDNF‐dependent synaptic changes. It is noteworthy that the *Adk* gene has two major transcripts (splice variants), encoding two protein isoforms that differ in the length of the amino terminus. Compared with the long isoform, the short isoform has a short amino terminus (4 vs 20 amino acids). Because the anti‐ADK antibody used here can not distinguish the two protein isoforms, our reported levels of ADK protein were the sum of both isoforms. *Bmal1*‐iKO did not completely abrogate the ADK rhythms in the small intestine (Figure 5B), although altered mesor and amplitude were noted (Table S1, Supporting Information). This suggests that the local clock system is not the only contributor to intestinal ADK oscillations. Other potential contributors include the feeding rhythm and the rest of the clock network (termed network‐dependency, which have been shown to determine the oscillations in a considerable portion of genes in the liver).^[^ ^82^ ^]^

In addition to a genetic disruption of intestinal BMAL1, pharmacological intervention (SR9009) was employed to assess the regulatory effects of the intestinal clock on cognitive behavior. Manipulations of intestinal BMAL1 by both methods result in cognitive impairments (Figure 1), solidifying the contribution of intestinal BMAL1 to cognitive function. The action of oral SR9009 (50–100 mg kg^−1^) is restricted to the intestine, having minimal effects on the liver clock due to poor oral bioavailability and exceedingly low liver exposure.^[^ ^24^ ^]^ SR9009 concentrations were < 5 ng mg^−1^ in hippocampus after oral gavage for 7 days, agreeing with the observation that oral SR9009 did not alter the expression of clock genes in the hippocampus either (Figure S2L, Supporting Information). Therefore, it is conceivable that oral SR9009 confers gut‐specific clock modulation owing to poor intestinal absorption and thus rather limited systemic effects. It is noteworthy that SR9009 has significant off‐target effects unrelated to REV‐ERB, influencing metabolism and mitochondrial activity.^[^ ^83^ ^]^ However, whether these off‐target effects contribute to cognitive phenotype remains unresolved.

Adenosine is a known factor influencing sleep and wakefulness.^[^ ^84^, ^85^ ^]^ No alterations in sleep‐wake behaviors were observed in *Bmal1*‐iKO mice despite changes in adenosine levels in the circulation and brain (Figure 3; Figure S2, Supporting Information). This discrepancy may be explained by at least three potential reasons. First, other sleep‐regulating substances such as glutamine besides adenosine were altered in *Bmal1*‐iKO mice. Glutamine is the precursor of glutamate, a neurotransmitter promoting wakefulness.^[^ ^86^ ^]^ There is a possibility that concurrent reductions in glutamine/glutamate counteract the effects on sleep‐wake behavior of adenosine changes. Second, *Bmal1*‐iKO‐induced change in adenosine (42–56% reductions in blood circulation) was insufficient to cause an alteration in sleep. Supporting this, *Adk^+/‐^* mice that have a 50% reduction of ADK showed normal sleep phenotype.^[^ ^84^ ^]^ Third, no sleep alteration in *Bmal1*‐iKO mice might be due to developmental effects that may rescue vital defects in animals. It is noteworthy that we did not observe significant changes in adenosine metabolites (such as AMP, ADP, ATP, and inosine, data not shown) in intestinal clock‐deficient mice despite the reductions in adenosine levels. Although the exact reason is unknown, this is probably because 1) a moderate reduction in adenosine caused by intestinal clock malfunction was not sufficient to elicit significant changes in all its metabolites, and 2) the metabolic enzymes and/or transporters specifically related to adenosine metabolites (e.g., PNP, APRT, CD38, and CD157) are altered simultaneously.^[^ ^46^ ^]^

While this study demonstrates that the intestinal clock shapes cognitive memory via adenosine‐A_1_R signaling and hippocampal LTP, we acknowledge that the potential role of GABAergic transmission in this process remains unexplored. GABAergic signaling, which critically regulates excitatory/inhibitory balance and synaptic plasticity, could theoretically interact with adenosine‐dependent pathways to modulate LTP and cognition.^[^ ^87^ ^]^ However, our focus here was to delineate the intestinal clock‐adenosine‐hippocampal axis as a primary mechanism underlying cognitive regulation. The robust rescue of LTP and memory deficits by adenosine supplementation or A_1_R agonism, alongside molecular evidence linking adenosine signaling to BDNF expression and synaptic remodeling, provides strong support for the sufficiency of this pathway in mediating the observed phenotypes. Future studies investigating crosstalk between adenosine and GABAergic systems in the context of intestinal clock‐driven cognitive regulation would further enrich our understanding of this complex circuitry.

Our study reveals that adenosine‐A_1_R signaling enhances hippocampal LTP by upregulating BDNF expression, a finding that appears to contrast with prior reports suggesting A_1_R activation suppresses LTP under physiological conditions.^[^ ^88^, ^89^ ^]^ This discrepancy may stem from distinct regulatory roles of A_1_R in pathological versus physiological states. In *Bmal1*‐iKO mice, systemic adenosine deficiency likely creates a “low‐adenosine” pathological milieu, where exogenous adenosine or A_1_R agonism restores synaptic plasticity by normalizing BDNF‐dependent signaling. This aligns with a prior study showing the context‐dependent effects of adenosine, where A_1_R‐mediated neuroprotection predominates in disease models.^[^ ^64^ ^]^ While we provide robust evidence for hippocampal CA1‐LTP as a key mechanism linking intestinal clock‐derived adenosine to cognition, we acknowledge potential contributions from other brain regions (e.g., prefrontal cortex or amygdala) to cognitive memory. Future studies mapping adenosine dynamics across brain circuits will further elucidate the gut‐brain axis in the circadian regulation of cognition.

In summary, the intestinal clock carries a cognition‐promoting effect, irrespective of the liver and hippocampal clocks. Mechanistically, the intestinal clock enhances local adenosine absorption through reducing ADK expression, and thus promotes adenosine‐A_1_R signaling in the hippocampus. Adenosine‐A_1_R signaling facilitates hippocampal LTP via enhancing BDNF expression and inhibiting synapse loss. These findings unravel the pivotal role of the intestinal clock in shaping cognitive capacity, and open a new avenue for the management of cognitive disorders.

### 3.1 Limitations of Study

The intestinal clock but not the liver clock regulates the circulating and brain levels of adenosine through modulation of adenosine metabolism. This may be due to a greater role of intestinal metabolism in determining the systemic bioavailability of adenosine as compared to hepatic metabolism. However, whether this is true or not requires further investigation. We have established the causality of intestinal clock deficiency upon hippocampal adenosine reduction. Unfortunately, it is unresolved whether adenosine reduction is cell type‐dependent in the hippocampus. Our findings suggest that adenosine signaling‐controlled BDNF expression, hippocampal synapses, and synaptic plasticity (LTP) may contribute to the intestinal clock gating of cognitive memory. However, whether adenosine signaling in astrocytes and microglia has a role in memory regulation by the intestinal clock remains unexplored. Glial adenosine signaling is previously shown to regulate astrocytic‐neuronal interactions and neuroinflammation, thereby modulating memory‐linked processes such as synaptic plasticity and neural network activity.^[^ ^90^, ^91^ ^]^ Three biological replicates per condition were used in RNA‐sequencing and metabolomic experiments, which is acknowledged as another study limitation because a low sample size may compromise the sensitivity and accuracy of differential expression measurements.

## 4 Experimental Section

All materials and methods are detailed in Supporting Information.

### Statistical Analysis

Data are presented as mean ± standard error of the mean (SEM). Statistical differences between two groups were analyzed using a two‐tailed Student's *t*‐test. Statistical differences between multiple groups were examined using one‐way or two‐way ANOVA followed by Bonferroni posttest. GraphPad Prism 8 (GraphPad Software Inc., San Diego, CA) was used for all statistical analyses. The specific types of tests are indicated in the figure legends. The rhythm characteristics such as M, A, and ψ were obtained by performing cosinor analysis (https://cosinor.online). A *p* value of less than 0.05 was considered statistically significant and displayed as follows: ^*^ *p*  <  0.05, ^**^ *p*  <  0.01, ^***^ *p* < 0.001 and ^****^ *p* < 0.0001.

## Conflict of Interest

The authors declare no conflict of interest.

## Author Contributions

M.C. and F.Z. contributed equally to this work. M.C., Z.F.Q., J.Y., and B.J.W. participated in research design. Experiments were conducted by M.C., F.G.Z., Y.F.X., X.J.J., Z.Q.Y., J.Y.W., X.T.L., J.L.C., L.N.H., H.C., and Y.K.D. Data analysis was performed by M.C., F.G.Z., X.J.J., Y.F.X., Z.Q.Y., J.Y.W., X.T.L., J.L.C., L.N.H., and B.J.W. The manuscript was written or contributed to by M.C. and B.J.W. All authors have read and approved the final manuscript.

## Supporting information



Supporting Information

## Data Availability

The data that support the findings of this study are available in the supplementary material of this article.
