# Supplementary material for: Intestinal Clock Promotes Cognitive Memory Through Adenosine Signaling
Source: Adv Sci (Weinh). 2025 Jun 20;12(34):e04526. doi: 10.1002/advs.202504526 (PMC12442615; doi:10.1002/advs.202504526)
Supplement: Supplementary file 1 — Supporting Information [file ADVS-12-e04526-s001.pdf]

ADVANCED SCIENCE
Open Access

Supporting Information

for *Adv. Sci.*, DOI 10.1002/advs.202504526

Intestinal Clock Promotes Cognitive Memory Through Adenosine Signaling

Min Chen, Fugui Zhang, Yifei Xiao, Xuejun Jiang, Zhiqian Yan, Jinyi Wang, Xiting Lv, Jialu Cui, Linna Ha, Hui Chen, Yongkang Dang, Zifei Qin, Jing Yang* and Baojian Wu**

Supplemental information

Intestinal *Bmal1* Promotes Cognitive Memory through Adenosine Signaling

Min Chen#, Fugui Zhang#, Yifei Xiao, Xuejun Jiang, Zhiqian Yan, Jinyi Wang, Xiting Lv, Jialu Cui, Linna Ha, Hui Chen, Yongkang Dang, Zifei Qin*, Jing Yang* and Baojian Wu*

*Corresponding author: Baojian Wu, Ph.D., E-mail: bj.wu@hotmail.com; Jing Yang, Ph.D., E-mail: jingyang_0101@163.com; Zifei Qin, Ph.D., E-mail: qzf1989@163.com

This PDF file includes:

Materials and methods
References (1 to 12)
Figures S1 to S12
Tables S1 to S3

MATERIALS AND METHODS

MATERIALS

REAGENT or RESOURCE	SOURCE	IDENTIFIER
Antibodies		
Mouse Monoclonal Anti-ADK	Santa	Cat# SC-365470
Rabbit Polyclonal Anti-PKC-Zeta	Proteintech	Cat# 26899-1-AP
Rabbit Polyclonal Anti-GSK3β	Proteintech	Cat# 22104-1-AP
Phospho-NF-κB p65 (Ser468) Recombinant antibody	Proteintech	Cat# 82335-1-RR
Phospho-GSK3B (Ser9) Monoclonal antibody	Proteintech	Cat# 67558-1-IG
Rabbit Polyclonal Anti-BDNF	Proteintech	Cat# 28205-1-AP
Rabbit Polyclonal Anti-GAPDH	Proteintech	Cat# 10494-1-AP
Rabbit Polyclonal Anti-REV-ERBα	Proteintech	Cat# 14506-1-AP
Rabbit Polyclonal Anti-Prkaca	Proteintech	Cat# 27398-1-AP
Rabbit Polyclonal Anti-Phospho-ERK1/2	Proteintech	Cat# 28733-1-AP
Rabbit Polyclonal Anti-Phospho-Creb1 (Ser133)	Proteintech	Cat# 28792-1-AP
Rabbit Monoclonal Anti-ADA	Proteintech	Cat# 67870-1-IG
Rabbit Polyclonal Anti-Entpd1	Proteintech	Cat# 14211-1-AP
Rabbit Polyclonal Anti-Slc28A3	Proteintech	Cat# 18182-1-AP
Rabbit Polyclonal Anti-Nt5e	Proteintech	Cat# 12231-1-AP
Rabbit Polyclonal Anti-ARNTL	Proteintech	Cat# 14268-1-AP
Rabbit Monoclonal Anti-Iba1	Abcam	Cat# ab178847
Rabbit Monoclonal Anti-eGFP	Servicebio	Cat# GB15603
Rabbit polyclonal Anti-DGKI	Affinity	Cat# DF3051
Rabbit polyclonal Anti-CRTC1	Novus	Cat# NBP1-89865
Rabbit polyclonal Anti-NETO1	Novus	Cat# NBP2-41134
Rabbit polyclonal Anti-SIPA1L1	Fine	Cat# FNab07873
FAM107A Rabbit pAb Antibody	Biorbyt	Cat# orb2308539
LGMN Rabbit Antibody	QYAOBIO	N/A
Rabbit UN13C polyclonal Antibody	BIOHUB	Cat# MBS9517720
Goat anti-Rabbit IgG(H+L)Cross Adsorbed Secondary Antibody, HRP	HuaBio	Cat# HA1001
Goat anti-Mouse IgG(H+L)Cross Adsorbed Secondary Antibody, HRP	HuaBio	Cat# HA1006
Chemicals, Peptides, and Recombinant Proteins		
Glucose	Aladdin	Cat# G116302
Adenosine	Shyuanye	Cat# B21356
ATP	Beyotime	Cat# S0027
CCPA	Aladdin	Cat# H614070
Corticosterone	APExBIO	Cat# B7469
DPCPX	MCE	Cat# HY-100937
Fumarate	Shyuanye	Cat# B20404
NECA	MCE	Cat# HY-103173
Nicotinate	Sigma	Cat# 72309
SR9009	MCE	Cat# HY-16989
SR8278	MCE	Cat# HY-14415

Calcium chloride	Aladdin	Cat# C108898
Magnesium chloride	Aladdin	Cat# M140955
Sodium phosphate monobasic dihydrogen	Sigma	Cat# 71505
Sodium bicarbonate	Sigma	Cat# S6297
Sodium chloride	Aladdin	Cat# C111533
Potassium chloride	Sigma	Cat# P9333
Sodium ascorbate	Aladdin	Cat# S105024
Trizol reagent	Invitrogen	Cat# 15596026
Critical Commercial Assays		
Enhanced chemiluminescence reagent	New Cell & Molecular	Cat# P10300
Protease Inhibitor Cocktail	MCE	Cat# HY-K0010
10% ExpressCast PAGE kit	New Cell & Molecular	Cat# P2012
PrimeScript RT Master Mix kit	Takara	Cat# RR036A
ChamQ SYBR qPCR master mix	Vazyme	Cat# Q311-02
SimpleChip plus Enzymatic Chromatin IP kit	Cell Signaling	Cat# 38191S
Dual Luciferase Reporter Gene Assay kit	Promega	Cat# E1910
Virus strains		
AAV_9-Villin-*Adk*-eGFP	This paper	N/A
AAV_9-Villin-eGFP	This paper	N/A
AAV_9-Villin-*Adk* shRNA-eGFP	This paper	N/A
AAV_9-Villin-shRNA-eGFP	This paper	N/A
AAV_1-hSyn-*Bdnf*-eGFP-FLAG	This paper	N/A
AAV_1-hSyn-eGFP-FLAG	This paper	N/A
$AAV_{2/9}$-hSyn-*Adora1*-eGFP-FLAG	This paper	N/A
$AAV_{2/9}$-hSyn-eGFP-FLAG	This paper	N/A
$AAV_{2/9}$-hSyn-*Adora1* siRNA-eGFP	This paper	N/A
$AAV_{2/9}$-hSyn-siRNA-eGFP	This paper	N/A
Experimental Models: Cell Lines		
CT26	Procell	Cat# CL-0071
HT22	Procell	Cat# CL-0697
Experimental models: Organisms/strains		
Villin-Cre mouse	Yu et al., 2021	N/A
Alb-Cre mouse	Cyagen Biosciences	N/A
Bmal1-flox mouse	Yu et al., 2021	N/A
Rev-erbα-flox mouse	Yu et al., 2021	N/A
Germ-free *Bmal1*-flox mouse	Chen et al., 2023	N/A
Germ-free *Bmal1*-iKO mouse	Chen et al., 2023	N/A
C57BL/6J mouse	HFK Biotechnology	N/A
Oligonucleotides		
Primers for qPCR See Table S2	This paper	N/A
Primer for ChIP See Table S3	This paper	N/A
Software and algorithms		
Image J software	NIH, open source	http://imagej-nih-gov.sire.ub.edu/ij/
Prism 8	GraphPad Softwate	http://www.graphpad.com/scientific-software/prism/
SMART v3.0, video tracking software	Panlab	https://www.panlab.com/en/products/smart-video-tracking-software-panlab

KEGG pathway database	N/A	https://www.kegg.jp/kegg/pathway.html
Other		
[^{3}H]-adenosine	Morawek	Lot #: 373-169-0204-A-20180712-NTO
AIN-76A diet added with 0.1% adenosine	Moldiets	Cat# M23070301
AIN-76A diet	Moldiets	Cat# M10001

Animal study

Bmal1-iKO*, Bmal1*-flox*, Rev-erbα*-iKO*,* and *Rev-erbα*-flox mice on a C57BL/6 background have been described.[1] *Vil1*-Cre line (B6.Cg-Tg(Vil1-cre)997Gum/J) were obtained from the Jackson Laboratory. *Bmal1*-flox or *Rev-erbα*-flox mice were crossed with *Vil1*-Cre mice to generate animals selectively lacking *Bmal1* or *Rev-erbα* in the intestine. *Albumin*-Cre (*Alb*-Cre) mice were obtained from Cyagen Biosciences (Guangzhou, China). *Bmal1*-flox mice were crossed with *Alb*-Cre mice to generate animals selectively lacking *Bmal1* in the liver. Mice were maintained on a 12 h light/dark cycle lights on at ZT0/7:00 AM, lights off at ZT12/7:00 PM with free access to water and food (if not specified). Light onset defines ZT0, thus ZT0-12 represents the light and ZT12-24 the dark period. *Bmal1*-iKO and *Bmal1*-flox mice were date mated and a cesarean section was performed on day 20 of fertility. The uterus was disinfected and transferred to germ-free (GF) isolator. After opening the uterus in the isolator, newborn (GF) mice were taken out, washed and put to a lactating GF foster mother of BALB/c strain.[2] 6- to 8-week-old male GF mice were used in the experiments. Animal experiments were performed using protocols approved by the Institutional Animal Care and Use Committee of Guangzhou University of Chinese Medicine.

Novel object recognition (NOR) and spatial object location (SOL) tests

NOR and SOL tests consisted of three phases: habituation, training and testing phase. For NOR test, during habituation (days 1-3), mice were allowed to explore the arena (40 cm × 40 cm × 40 cm) for 10 min. In the training phase (day 4), mice were allowed to explore two identical objects (equidistantly spaced in the arena) for 10 min and returned to their home cages immediately after training. The testing phase was conducted 2 h (for short-term memory) or 24 h (for long-term memory) after the training phase. In the testing phase, one of the objects was replaced by a new one (novel object) and the mouse was allowed to explore them for 10 min. For SOL test, after habituation, mice were exposed to two identical objects for 10 min during the familiarization phase. Testing occurred 2 h later in the same arena. Mice were allowed to explore for 10 min the same arena but one of the familiar objects was moved to a novel location. Mice were excluded from the experiment if they explored the objects for < 20 s during the training or testing phase.[3,4] The time spent to explore the objects were analyzed using a computerized image analyzing system (Panlab Harvard Apparatus, Barcelona, Spain). Discrimination indices were calculated as: (Time for novel object exploring – time for familiar object exploring)/(Time for novel object exploring + time for familiar object exploring). In SOL test, memory was operationally defined by the

percentage of alteration index, which was calculated as: (Time spent on exploring novel located object – time spent on exploring familiar located object)/(Time spent on exploring novel located object + time spent on exploring familiar located object) × 100%.

For NOR diurnal experiments, mice of each genotype were divided into four groups: group ZT0, group ZT6, group ZT12, and group ZT18. Training of group ZT0 was started at ZT22 and 2 h later testing was initiated. Training of group ZT6 was started at ZT4 and 2 h later testing was initiated. Training of group ZT12 was started at ZT10 and 2 h later testing was initiated. Training of group ZT18 was started at ZT16 and 2 h later testing was initiated. For NOR circadian experiments, mice were kept under constant darkness for 3 days. On day 4, mice were subjected to behavioral testings at different circadian time (CT) intervals.

Y maze test

The Y maze test was performed as previously described to assess spontaneous alternation, which was defined as successive entries into the three arms in overlapping triplet sets.[5] The maze was made up of three arms and converged to an equal angle (Ruiwode Lift Technology, Shenzhen, China). Mouse was placed in the center of the apparatus and was allowed to explore it for 10 min. The total numbers of arm entries and alternations were recorded. The alternation percentage was calculated as the ratio of actual to possible alternations (defined as (the total number of arm entries -2) × 100%).

Other behaviour tests

Forced swim test (FST), tail suspension test (TST), novelty-suppressed feeding test (NSFT), open field test (OFT), elevated plus maze (EPM), and three-chamber social tests were performed with mice as previously described.[6-8] Animal behaviors were recorded by using a SMART video tracking system and analyzed with a SMART 3.0 software (Panlab Harvard Apparatus, Barcelona, Spain).

FST：Mice were forced to swim for 6 min in an acrylic circular cylinder (35 cm in diameter, 30 cm in depth) filled with room temperature water, and the total time spent immobile was recorded.

TST: Mice were suspended for 6 min by their tails using adhesive tape. The total time spent immobile during the trial were recorded and immobility was defined as hanging passively without any movement of the head or paws.

NSFT: Mice were fasted for 24 h and then subjected to NSFT. Mouse was placed at the corner of a white acrylic box (40 cm × 40 cm) containing food and allowed to eat the food. The time starting to eat the food was recorded.

OFT: Mice were placed in the center of a square (40 cm × 40 cm × 40 cm) open field box and allowed to freely explore it for 10 min. The distance and time spent in the center as well as total distance moved were recorded using SMART 3.0 software (Panlab Harvard Apparatus, Barcelona, Spain).

EPM: EPM consisted of 2 open arms and 2 closed (wall sheltered) arms (25 cm × 5 cm). This structure was elevated 60 cm above the floor. Mice were placed in the center of EPM facing towards a closed arm, then allowed to explore freely for 5 min. Time spent in the closed arms and open arms was recorded.

Three-chamber social tests: The three-chamber was a 60 cm × 40 cm plexiglass box divided into three equally sized and interconnected chambers (i.e., left, center and right). Sociability and social novelty preference were performed as previously described.[6] Briefly, a mouse was first habituated to the empty arena for 5 min. For measuring sociability, this mouse was allowed to interact with an empty wire cup (empty) or an age- and sex-matched stranger conspecific in the other wire cup (mouse 1) for 5 min. Time spent interacting (e.g., sniffing and crawling upon) with either the empty cup or mouse 1 was recorded. For assessing preference for social novelty, a second stranger mouse (mouse 2) was introduced into previously empty cup. The test mouse was allowed to interact with mouse 1 or mouse 2 for 5 min. Time spent interacting with either mouse 1 or mouse 2 was recorded.

Diurnal expression and adenosine profiling

Entire small intestine (intestinal epithelial cells only) and hippocampus were collected every 6 h (ZT0, ZT6, ZT12, and ZT18) throughout a light/dark cycle from *Bmal1*-iKO, *Rev-erbα*-iKO and control mice, followed by qPCR and immunoblotting.

Electrophysiology

Hippocampal slices (400 μm thick) were prepared from mouse brain, incubated at 32°C for 30 min and maintained at 26°C for 1 h as described previously (Chen et al., 2022). After recovery, slices were placed in a recording chamber at 25°C and perfused with oxygenated artificial cerebrospinal fluid (ACSF) containing 125 mM NaCl, 5 mM KCl, 1.2 mM NaH_2PO_4, 26 mM $NaHCO_3$, 10 mM glucose, 2 mM $CaCl_2$, and 1.3 mM $MgCl_2$ at a rate of 1 ml/min. Extracellular field EPSPs (fEPSPs) were recorded from the CA1 area using a glass electrode filled with ACSF (2-3 MΩ). Schaffer collateral pathway was stimulated every 30 s using concentric bipolar electrodes. Stimulation intensity was adjusted to evoke fEPSP amplitudes that were 30-50% of maximal size. A theta-burst stimulation (TBS) protocol (four pulses of 100 Hz repeated three times at 5 Hz with a 20 s inter-train interval) was used to induce LTP.[5] Recording signals were obtained with a patch-clamp amplifier (EPC10 USB, HEKA), and data were analyzed using the Clampex software (Axon Instruments, Foster City, CA). Paired-pulse facilitation (PPF) was calculated as the ratio of the second fEPSP slope to the first fEPSP slope (an inter-stimulus interval of 50 ms) across 5 trials. PPF ratios were averaged and normalized to baseline values.

Chemical treatment

Bmal1-iKO, *Rev-erbα*-iKO and control mice were gavaged with adenosine at a dose of 5 mg/kg once daily for 21 days. On day 22, mice were subjected to cognitive tests, and then sacrificed to collect blood, small intestine and hippocampus. In another experiment, mice

were fed a chow diet (AIN-76A) containing 0.1% adenosine for 14 days. On day 15, mice were subjected to cognitive tests, and then sacrificed to collect small intestine and hippocampus.

Wild-type mice were gavaged with SR9009 (50 and 100 mg/kg) or vehicle once daily for 7 days. On day 8, mice were subjected to cognitive tests, and then sacrificed to collect small intestine and hippocampus. *Bmal1*-iKO, *Rev-erbα*-iKO and control mice were intraperitoneally injected with CCPA (0.05 mg/kg) or vehicle once daily for 4 days. On day 5, mice were subjected to cognitive tests. *Bmal1*-iKO and control mice were intraperitoneally injected with DPCPX (1 mg/kg) or vehicle once daily for 7 days. On day 8, mice were subjected to cognitive tests. *Bmal1*-iKO, *Rev-erbα*-iKO and control mice were intraperitoneally injected with NECA (0.08 mg/kg) or vehicle once daily for 14 days. On day 15, mice were subjected to cognitive tests. *Bmal1*-iKO and control mice were intraperitoneally injected with SCH58261 (0.1 mg/kg) or vehicle once daily for 21 days. On day 22, mice were subjected to cognitive tests. *Bmal1*-iKO and control mice were intraperitoneally injected with CGS21680 (0.1 mg/kg) or vehicle once daily for 21 days. On day 22, mice were subjected to cognitive tests.

We designed our studies to account for and minimize stress-related confounding factors: 1) behavioral tests were initiated after a 24 h recovery interval to allow mice to fully recover from any acute stress associated with the gavage procedure; and 2) all experimenters were rigorously trained to ensure gentle and consistent handling, reducing procedural variability and stress.

Determination of [^{3}H]-adenosine in vivo

[2,8-^{3}H]-adenosine (specific activity: 20.4 Ci/mmol; Lot #: 373-169-0204-A-20180712-NTO) was obtained from Morawek Inc (Brea, CA). 5 mg/kg adenosine containing 50 μCi [^{3}H]-adenosine was administered to wild-type mice via oral gavage. Blood samples were collected at 15, 30, 45, 60, 75 and 90 min using microdialysis technique as described in "microdialysis sampling" section. Hippocampal microdialysis samples were collected at 90 min. Total radioactivities in blood were measured by using a liquid scintillation counter (LS 6500, Beckman Coulter, Brea, CA). Quantitative determination of [^{3}H]-adenosine in blood and hippocampus was performed using a validated LC-MS/MS method. A 90 μl aliquot of microdialysis sample was injected into an ultra-high performance liquid chromatography (UHPLC)-fraction collector system (Thermo). The UHPLC eluent was collected in Deepwell LumaPlate 96 plates (Perkin Elmer, Waltham, MA) for first 15 min with every 8 s eluent collected in one well. After evaporation, the radioactivity was determined using an online radioactivity detector (β-RAM). Data were reconstructed into a radiochromatogram by using Laura software (Lablogic, Broomhill, Sheffield, UK). Another 10 μl aliquot of microdialysis sample was injected for mass spectrometry (MS) analysis.

Antibiotic cocktail (ABX) treatment

Gut microbiota were depleted in adult *Bmal1*-iKO and *Bmal1*-flox (male) mice by treatment

with a cocktail of antibiotic for 3-4 weeks. The recipe included drinking water with ampicillin (1 g/l), neomycin (1 g/l), vancomycin (0.5 g/l), and metronidazole (0.5 g/l). Faecal samples were freshly collected before behavioral tests and plated onto Brucella agar plates with 5% sheep's blood and cultured aerobically and anaerobically to test whether gut bacteria were successfully depleted following ABX treatment.

Intrahippocampal injection

Mini-osmotic pumps (1001W, Ruiwode Life Science, China) were implanted subcutaneously between the shoulder blades. Pumps were connected to bilateral chronic indwelling cannulae (Bic-3) which aimed at the hippocampus (anteroposterior: −2.0 mm from the bregma, ventral: −3.0 mm below the dura, and lateral: ±2.5 mm from the midline). All mini-osmotic pumps were equilibrated in saline for 24 h at 37°C before implantation. Implanted mini-pumps were filled with CCPA (0.4 ng/μl) or vehicle, and administered at a rate of 0.25 l/h. After 4 days' infusion, mice were subjected to behavioral testing. In another experiment, implanted mini-pumps were filled with DPCPX (0.8 ng/μl) or vehicle, and administered at a rate of 0.25 l/h. After 7 days' infusion, mice were subjected to behavioral testing.

Golgi staining

Brain samples were incubated in dying solution (made of 5% chloral hydrate, 5% potassium dichromate and 4% formaldehyde) for 3 days and transferred to a solution containing 1% silver nitrate for another 3 days in dark. The brain samples were cut into 60 μm coronal sections. Six randomly selected hippocampal sections from each of 3 mice were matched for plane of cut. The images at CA1 pyramidal neurons were taken by using bright-field microscopy (Axioplan 2; Zeiss, Brighton, MI). Morphology analysis was performed using ImageJ software. Using the center of soma as a reference point, dendritic length and branch points were measured as a function of radial distance from the soma by adding up all values in each successive concentric segment (Sholl's analysis).[9] Total dendritic length, the number of branch points, and the number of primary dendrites were analyzed for every neuron. Spine density was determined for dendrites at a distance of 90-150 μm from the soma.

Adeno-associated virus (AAV) infection

AAV_9 expressing *Adk-S* under the control of *Villin* promoter (AAV_9-*Villin-Adk*-eGFP, referred to as AAV_9-ADK) was administered to wild-type mice via tail vein injection at 3.0×10^{11} vg/ml (200 μl per mouse). Three weeks later, mice were sacrificed, and small intestine and hippocampus were collected for gene expression. In another set of experiments, mice were treated with AAV_9-ADK or control (AAV_9-*Villin*-empty-eGFP, referred to as AAV_9-empty). Three weeks later, NOR and Y maze tests were performed to assess cognitive performance.

Adk-S knockdown recombinant AAV_9-*Villin* (AAV_9-*Villin-Adk* shRNA-eGFP, referred to as AAV_9-shADK) was administered to *Bmal1*-iKO and control mice via tail vein injection at 1.0×10^{12} vg/ml (200 μl per mouse). Three weeks later, mice were sacrificed, and small intestine and hippocampus were collected for gene expression. In another set of experiments, *Bmal1*-

iKO and control mice were treated with AAV_9-shADK or control (AAV_9-scrambled shRNA). Three weeks later, NOR and Y maze tests were performed to assess cognitive performance.

AAV_1 expressing *Bdnf* or empty AAV_1 was administered to *Bmal1*-iKO and control mice via intra-hippocampal microinjection at 1.0 × 10^{12} vg/ml. Briefly, a total volume of 2.0 μl AAV vectors (1.0 μl per side) were delivered bilaterally into the hippocampus at 0.2 μl/min. Behavioral testing commenced 3 weeks following viral injection, and then mice were perfused transcardially and brain tissues were processed for Golgi staining. $AAV_{2/9}$ expressing *Adora1* or empty $AAV_{2/9}$ was administered to *Bmal1*-iKO and control mice via intra-hippocampal microinjection at 2.2 × 10^{12} vg/ml. *Adora1* knockdown recombinant $AAV_{2/9}$ ($AAV_{2/9}$-*Adora1* siRNA-eGFP, referred to as $AAV_{2/9}$-siA_1R) or AAV-scrambled siRNA was administered to wild-type mice via intra-hippocampal microinjection at 1.3 × 10^{12} vg/ml. A total volume of 2.0 μl AAV vectors (1.0 μl per side) were delivered bilaterally into the hippocampus at 0.2 μl/min. Behavioral testing commenced 3 weeks following viral injection, and then mice were sacrificed and hippocampal samples were collected for gene expression analysis. Information of AAV is provided in KEY RESOURCES TABLE. All AAV injections were performed with mice aged 6-8 weeks.

RNA-sequencing

Three *Bmal1*-iKO mice and three control mice were sacrificed and the hippocampus were collected at each of two diurnal time points (i.e., ZT6 and ZT18). RNA was isolated using Trizol (Invitrogen, Carlsbad, CA) and its quality was checked using an Agilent 2100 Bioanalyzer (Santa Clara, CA). The samples were considered qualified when RIN > 7.7.[10] RNA-sequencing was performed as described in our previous report.[10] Sequencing libraries were prepared using NEBNext Ultra RNA library Prep Kit for Illumina (Ipswich, MA). Sequencing was performed on an Illumina HiSeq X Ten for 150 bp length reads. Clean reads were obtained by removing those containing an adapter and ploy-N using FASTQ program, and aligned to mouse GRCm38/mm10 genome using HISAT2 v2.0.4 with default parameters.[10] Read counts were normalized to FPKM values using DESeq2 (v.1.16.1). Genes were defined as differentially expressed when adjusted $p < 0.05$. GO analysis was conducted using the clusterProfiler R package.

Cell culture and treatment

CT26 cells were cultured in RIPA1640 medium supplemented with 10% FBS and 1% penicillin/streptomycin. Cells were transfected with overexpression plasmid (*Rev-erbα* or *E4bp4* or *Dec2*) or siRev-erbα or control using JetPRIME (Polyplus Transfection, Ill kirch, France). After 48 h, cells were collected for qPCR and immunoblotting.

In vitro metabolic assays

CT26 cells were cultured in 6-well plates. After attachment, cells were transfected with siBmal1 or control using JetPRIME (Polyplus Transfection, Ill kirch, France). 48 h later, cells were incubated in Krebs buffer containing 100 nM adenosine and 100 nM ATP for 2 h.

Adenosine in the incubation solution was quantified using a LC-MS/MS system.

Small intestine and livers were collected from *Bmal1*-iKO and control mice. Mouse S9 fraction were prepared by centrifugation at 9000 g for 10 min to determine ADK activities.[11] In brief, S9 fraction (4 mg/ml) were incubated with adenosine (100 nM), ATP (100 nM), and $MgCl_2$ (5 mM) in 50 mM potassium phosphate (pH = 7.4) for 2 h at 37°C. The reaction was terminated by adding ice-cold methanol. The resulting mixture was centrifuged at 13,000 g for 15 min, and the supernatant was subjected to LC-MSMS analysis.

qPCR

Total RNA was extracted with TRIzol reagent (Invitrogen, Carlsbad, CA). cDNA synthesis was performed with PrimeScript RT Master Mix kit (Takara, Shiga, Japan). qPCR reactions were performed with SYBR green PCR master mix (Applied Biosystems) using a Biometra Toptical Thermocycler (Analytik Jena, Goettingen, Germany). Relative mRNA level for each gene was calculated using the $2^{-\Delta\Delta Ct}$ method and normalized to *Ppib* or *β-actin*. Primers are listed in SUPPLEMENTAL TABLE 2.

Immunoblotting

Protein samples were separated by sodiumdodecyl sulfate-polyacrylamide gel electrophoresis (Millipore, Bedford, MA), and then transferred onto a polyvinylidene difluoride membrane. The membrane was sequentially incubated with primary antibody and secondary antibody. Bands were visualized with enhanced chemiluminescence using an Omega LumG Imaging System (Aplegen, Pleasanton, CA) and band densities were quantified with FluorChem 5500 (Alpha Innotech, San Leandro, CA). GAPDH was used as an internal control. Information of primary antibodies is provided in KEY RESOURCES TABLE.

Luciferase reporter assay

CT26 cells were co-transfected with a *Adk* P1 or *Adk* P2 luciferase reporter (-2.0 kb), pRL-TK, and overexpression plasmid (*Bmal1 or Rev-erbα* plasmid) or blank pcDNA3.1 using JetPRIME (Polyplus Transfection, Ill kirch, France). 24 h later, cells were collected and luciferase activities were measured using Dual-Luciferase Reporter Assay System (Promega, Madison, WI). Firefly luciferase activity was normalized to renilla luciferase activity, and expressed as a relative luciferase unit (RLU).

Chromatin immunoprecipitation (ChIP) assays

ChIP assays were performed using a SimpleChip plus Enzymatic Chromatin IP kit (Cell Signaling Technology, Beverly, MA). Briefly, mouse small intestine was fixed in 37% formaldehyde and lysed in lysis buffer, and then digested by micrococcal nuclease. Sheared chromatin (~150 bp) was immunoprecipitated with anti-REV-ERBα antibody or normal IgG (a negative control) overnight at 4°C. The immune complex was decross-linked at 65°C for 6 h.

The obtained DNAs were purified and analyzed by qPCR with specific primers (provided in SUPPLEMENTAL TABLE 3).

Metabolomics

Intestinal metabolites were extracted with 80% methanol, followed by centrifugation at 20,000 g for 15 min. The supernatant was subjected to analysis by Orbitrap Exploris 120 mass spectrometer coupled with Vanquish Flex UHPLC system (Thermo Fisher Scientific, Cleveland, OH). Chromatographical separation was performed with an ACQUITY UPLC HSS T3 column (100 × 2.1 mm, 1.8 μm, Waters, Milford, MA). Flow rate was set at 0.25 ml/min. Mass spectrometer was operated in both positive and negative ion modes. The mobile phases consisted of (A) water (containing 5 mM ammonium acetate and 5 mM acetic acid) and (B) acetonitrile. Gradient elution program was 0-0.5 min, 2% B; 0.5-4.0 min, 2-98% B; 4.0-9.9 min, 98% B; 9.9-10.0 min, 98-2% B; 10.0-14.0 min, 2% B. Raw data were converted to mzXML format by MSConvert with ProteoWizard software package (v3.0.8789) and processed using XCMS for feature detection, retention time correction and alignment. HMDB and KEGG (Kyoto Encyclopedia of Genes and Genomes) database were used to annotate metabolites. Differential metabolite were defined when p value was < 0.05 and VIP (variable importance projection) was > 1.

Microdialysis sampling

Mice were anesthetized and mounted in a stereotaxic apparatus. A guide cannula (AG-X, Eicom, Japan) was stereotaxically implanted into the hippocampus at the following coordinates: −2.9 mm from the bregma, −2.5 mm below the dura, and +3.0 mm from the midline. Mice were allowed to recover from surgery for 3 days. On day 4, the guide cannula dummy stylet was removed and replaced with a brain microdialysis probe with a 2 mm membrane (FZ-2.5-2, Eicom, Japan). Oxygenated artificial cerebrospinal fluid was perfused through the probe at 1 μl/min for the dialysis via a syringe pump (Eicom/ESP-64). For small intestine and liver implantation, mice were anesthetized with sodium pentobarbital (50 mg/kg). After laparotomy of the abdomen, the small intestine and liver were exposed. A flexible concentric proben (TP-80-2, Eicom, Japan) was implanted into the jejunum, and a linear microdialysis probe (OP-50-3, Eicom, Japan) was implanted into the liver. Ringer's solution (NaCl 137 mM, KCl 1.0 mM, $CaCl_2$ 0.9 mM, and $NaHCO_3$ 1.2 mM) was perfused through the probe with a flow rate of 1 μl/min. For blood sampling, mice were anesthetized and jugular vein or hepatic portal vein was exposed via a thoracotomy or laparotomy. A flexible concentric probe (TP-80-2, Eicom, Japan) was implanted into the vein via the cannula. Ringer's solution was perfused through the probe with a flow rate of 1 μl/min. The dialysate fractions were automatically collected and maintained at 4°C. Microdialysis samples (30 μl) were injected into the LC-MS/MS system for adenosine analysis.

LC-MS/MS

Blood, hippocampus, and small intestine samples were collected. Hippocampus and small intestine samples were homogenized at a ratio of 1/10 (*w/v*) in saline solution. Microdialysis samples, blood samples and tissue homogenates were processed and then subjected to analysis by using a Nexera XP UHPLC system (Shimadzu, Kyoto, Japan) equipped with a Shim-pack GIST C18 column (100 mm × 2.1 mm, 2.1 μm). The column temperature was maintained at 40°C. The mobile phases consisted of methanol (A) and water acidified with 0.1% formic acid (B). Total flow rate was set at 0.2 ml/min. For adenosine analysis, gradient elution program was 0-3 min, 90% B; 3-3.5 min, 90-3% B; 3.5-5 min, 3% B; 5-5.5 min, 3-90% B; and 5.5-7 min, 90% B. Mass spectrometer was operated in the positive ion multiple reaction monitoring mode. The mass transition ion pair for adenosine was *m/z* 268.28→136.15 Da. For fumarate analysis, gradient elution program was 0-2.5 min, 60% B; 2.5-3.5 min, 60-15% B; 3.5-4.5 min, 15% B and 4.5-5.5 min, 60% B. Mass spectrometer was operated in the negative ion multiple reaction monitoring mode. The mass transition ion pair for fumarate was *m/z* 115.15→71.1 Da. For nicotinate analysis, the mobile phases consisted of acetonitrile (A) and water acidified with 0.1% formic acid (B). Gradient elution program was 0-1.5 min, 55% B; 1.5-3.5 min, 55-90% B; 3.5-4.0 min, 90% B; and 4.0-5.0 min, 55% B. Mass spectrometer was operated in the positive ion multiple reaction monitoring mode. The mass transition ion pair for nicotinate was *m/z* 124.2→78.15 Da.

Analysis of fatty acids by GC-MS

Fatty acids were converted to methyl esters for analysis as previously described.[12] Briefly, fatty acids in hippocampus were extracted with methanol, and incubated for 1 h at 70°C in 2 ml of 2% sulfuric acid in methanol. The reaction was stopped by adding 1 ml of water and then mixed thoroughly with hexane (200 μl). The hexane layer was collected and injected into a gas chromatography time-of-flight mass spectrometry (GC-TOFMS; LECO's Pegasus BT, MI, US). Helium (99.99%) at a constant flow rate of 1 ml/min was used as carrier gas. Initial oven temperature was set at 80°C (held for 2 min) and increased to 170°C with a 10°C/min rate. 170°C was held for 2 min, increased to 205°C with a rate of 2.0°C/min, and continually increased to 240°C with a 10°C/min rate. The final temperature was kept for 10 min. MS conditions: ionization energy, 70 eV; ion source temperature, 220°C; and the mass-to-charge (*m/z*) range, 50-550.

Immunofluorescence

Brain samples were fixed, paraffin-embedded, and cut into 20 μm coronal sections. Sections were blocked with 5% bovine serum albumin in phosphate-buffered saline (PBS) containing 0.1% Triton X-100, and then incubated with anti-Iba1 antibody (KEY RESOURCES TABLE). After washing with PBS, sections were incubated with a fluorescent secondary antibody and DAPI (4',6-diamidino-2-phenylindole). Images were captured using a Nikon Optiphot fluorescent microscope (Tokyo, Japan).

Immunohistochemistry

Brain, small intestine, liver and stomach samples were fixed, paraffin-embedded, and cut into 10 μm sections. Sections were blocked with 3% bovine serum albumin in PBS, and then incubated with anti-ADK or anti-eGFP antibody (KEY RESOURCES TABLE). After washing with PBS, sections were incubated with a secondary antibody. Nuclear counterstaining was performed using hematoxylin. Images were captured using a Nikon Optiphot fluorescent microscope (Tokyo, Japan).

Mouse model of delirium

Bmal1-iKO mice and controls were treated with LPS (200 μg/kg) plus midazolam (10 mg/kg, i.p., named LM treatment) to induce delirium-like syndrome as previously described.[5] 24 h later, mice were subjected to behavioral testing. In another set of experiments, wild-type mice were gavaged with SR8278 (100 mg/kg) or vehicle once daily for 7 days. On day 7, LM treatment was initiated. 24 h later, mice were subjected to cognitive tests, and then sacrificed to collect small intestine. We assessed the cognitive performance because it is a core feature of delirium, although other pathologic manifestations such as attentional deficit are also noted.[5]

EEG (electroencephalogram) and EMG (electromyography) recordings

Mice were anesthetized and mounted in a stereotaxic apparatus. Screw electrodes were inserted into the skull of mice to measure cortical EEG using the following coordinates: +2 mm Bregma, +1 mm midline for first recording electrode; +2 mm Bregma, −1 mm midline for second recording electrode; −2 mm Bregma, −1 mm midline for a reference electrode; and −2 mm Bregma, +1 mm midline for a ground electrode. Stainless steel electrodes were implanted in dorsal neck muscle to measure EMG. After recovery for 7 days, *Bmal1*-iKO and control mice were subjected to EEG and EMG recordings. Data were acquired and EEG signals were filtered as previously described.[5] In brief, data were acquired using a tethered data acquisition system with a resolution of 500 Hz (Medusa, Biosignal technologies, Nangjing, China), and waveforms visualized using Sirenia Sleep Pro software (Pinnacle technologies, Lawrence, KS). EEG signals were high-pass filtered (> 0.5 Hz) using a digital filter and EMG was band-pass filtered between 5 and 45 Hz. Power in the δ (0.5-4 Hz), θ (5-8 Hz), α (9-14 Hz) bands, and θ to δ band ratio were calculated, and EMG signal was scored in 4 s epochs. All data were used to define the vigilance states of wake, NREM (non-rapid eye movement), and REM (rapid eye movement) sleep by using an automatic script (Lunion Stage software, LunionData, Nangjing, China).

REFERENCES

1 Yu F, Wang Z, Zhang T, Chen X, Xu H, Wang F, et al. Deficiency of intestinal Bmal1 prevents obesity induced by high-fat feeding. Nat Commun. 2021;12(1):5323.

2 Stepankova R, Tonar Z, Bartova J, et al. Absence of microbiota (germ-free conditions) accelerates the atherosclerosis in ApoE-deficient mice fed standard low cholesterol diet[J]. Journal of atherosclerosis and thrombosis, 2010;17(8): 796-804.

3 Leger M, Quiedeville A, Bouet V, Haelewyn B, Boulouard M, Schumann-Bard P, Freret T. Object recognition test in mice. Nat Protoc. 2013;8(12):2531-7.

4 Cohen SJ, Stackman RW Jr. Assessing rodent hippocampal involvement in the novel object recognition task. A review. Behav Brain Res. 2015;285:105-17.

5 Chen M, Zhang L, Shao M, Du J, Xiao Y, Zhang F, Zhang T, Li Y, Zhou Q, Liu K, Wang Z, Wu B. E4BP4 Coordinates Circadian Control of Cognition in Delirium. Adv Sci (Weinh). 2022;9(23):e2200559.

6 Liu X, Li X, Xia B, Jin X, Zou Q, Zeng Z, Zhao W, Yan S, Li L, Yuan S, Zhao S, Dai X, Yin F, Cadenas E, Liu RH, Zhao B, Hou M, Liu Z, Liu X. High-fiber diet mitigates maternal obesity-induced cognitive and social dysfunction in the offspring via gut-brain axis. Cell Metab. 2021;33(5):923-938.e6.

7 Ramírez S, Haddad-Tóvolli R, Radosevic M, Toledo M, Pané A, Alcolea D, Ribas V, Milà-Guasch M, Pozo M, Obri A, Eyre E, Gómez-Valadés AG, Chivite I, Van Eeckhout T, Zalachoras I, Altirriba J, Bauder C, Imbernón M, Garrabou G, Garcia-Ruiz C, Nogueiras R, Soto D, Gasull X, Sandi C, Brüning JC, Fortea J, Jiménez A, Fernández-Checa JC, Claret M. Hypothalamic pregnenolone mediates recognition memory in the context of metabolic disorders. Cell Metab. 2022;34(2):269-284.e9.

8 Lu SY, Fu CL, Liang L, Yang B, Shen W, Wang QW, Chen Y, Chen YF, Liu YN, Zhu L, Zhao J, Shi W, Mi S, Yao J. miR-218-2 regulates cognitive functions in the hippocampus through complement component 3-dependent modulation of synaptic vesicle release. Proc Natl Acad Sci U S A. 2021;118(14):e2021770118.

9 Jiang X, Chai GS, Wang ZH, Hu Y, Li XG, Ma ZW, Wang Q, Wang JZ, Liu GP. Spatial training preserves associative memory capacity with augmentation of dendrite ramification and spine generation in Tg2576 mice. Sci Rep. 2015;5:9488.

10 Zhang T, Yu F, Xu H, Chen M, Chen X, Guo L, Zhou C, Xu Y, Wang F, Yu J, Wu B. Dysregulation of REV-ERBα impairs GABAergic function and promotes epileptic seizures in preclinical models. Nat Commun. 2021;12(1):1216.

11 Richardson SJ, Bai A, Kulkarni AA, Moghaddam MF. Efficiency in Drug Discovery: Liver S9 Fraction Assay As a Screen for Metabolic Stability. Drug Metab Lett. 2016;10(2):83-90.

12 Shi X, Li J, Zou X, Greggain J, Rodkær SV, Færgeman NJ, Liang B, Watts JL. Regulation of lipid droplet size and phospholipid composition by stearoyl-CoA desaturase. J Lipid Res. 2013;54(9):2504-14.

SUPPLEMENTAL FIGURES

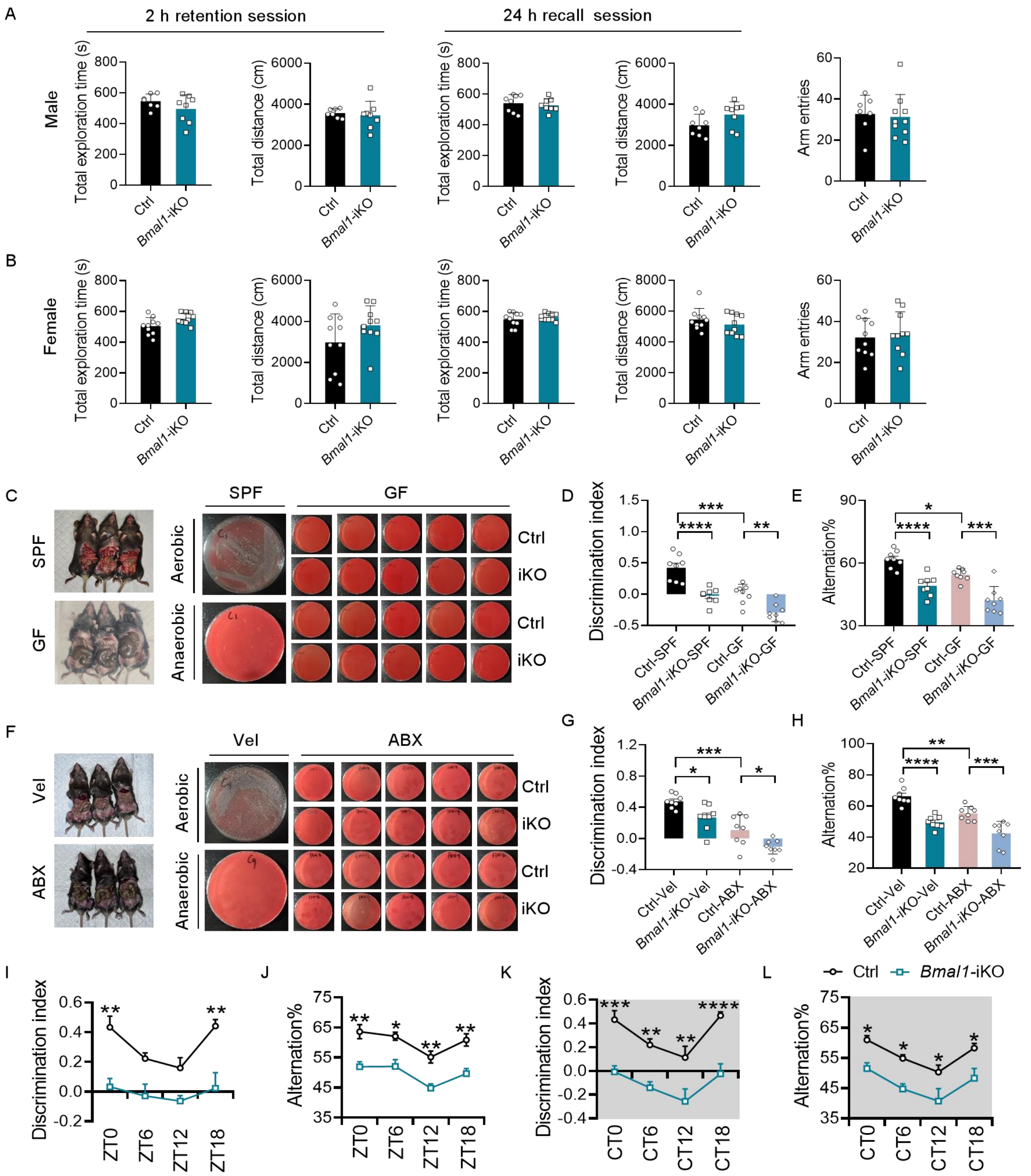

Figure S1 A, Total exploration time and total distance in NOR test during 2 h retention session (left) and during 24 h recall session (middle) in male *Bmal1*-iKO and control mice (*n* = 7~8). Arm entries in Y maze test (right) in male *Bmal1*-iKO (*n* = 7) and control (*n* = 10) mice. B, Total exploration time and total distance in NOR test during 2 h retention session (left) and during 24 h recall session (middle) in female *Bmal1*-iKO and control mice (*n* = 10). Arm entries in Y maze test (right) in female *Bmal1*-iKO and control mice (*n* = 10). C, Representative images of caecum enlargement and brucella blood agar plating of intestinal contents from male germ-free (GF) and

SPF mice. D, Discrimination index in NOR test during 2 h retention session in GF *Bmal1*-iKO and control mice (n = 8). E, Spontaneous alternations in Y maze test in GF *Bmal1*-iKO and control mice (n = 8). F, Representative images of caecum enlargement and brucella blood agar plating of intestinal contents from male ABX- and vehicle-treated mice. G, Discrimination index in NOR test during 2 h retention session in ABX- and vehicle-treated mice (n = 8). H, Spontaneous alternations in Y maze test in in ABX- and vehicle-treated mice (n = 8). I, Diurnal changes in object preference for *Bmal1*-iKO and control mice (n = 8). J, Diurnal changes in spontaneous alternations for *Bmal1*-iKO and control mice (n = 8). K, Circadian changes in object preference for *Bmal1*-iKO and control mice (n = 8). L, Circadian changes in spontaneous alternations for *Bmal1*-iKO and control mice (n = 8). Discrimination indices were calculated as: (Time for novel object exploring – time for familiar object exploring)/(Time for novel object exploring + time for familiar object exploring). All behavioral tests except tests in panels I-L were conducted at ZT6. Data are mean ± SEM, and analyzed by two-way ANOVA with Bonferroni posttest (D, E, G, H and I-L). *$p < 0.05$, **$p < 0.01$, ***$p < 0.001$, ****$p < 0.0001$. GF, germ-free; ABX, antibiotic cocktail.

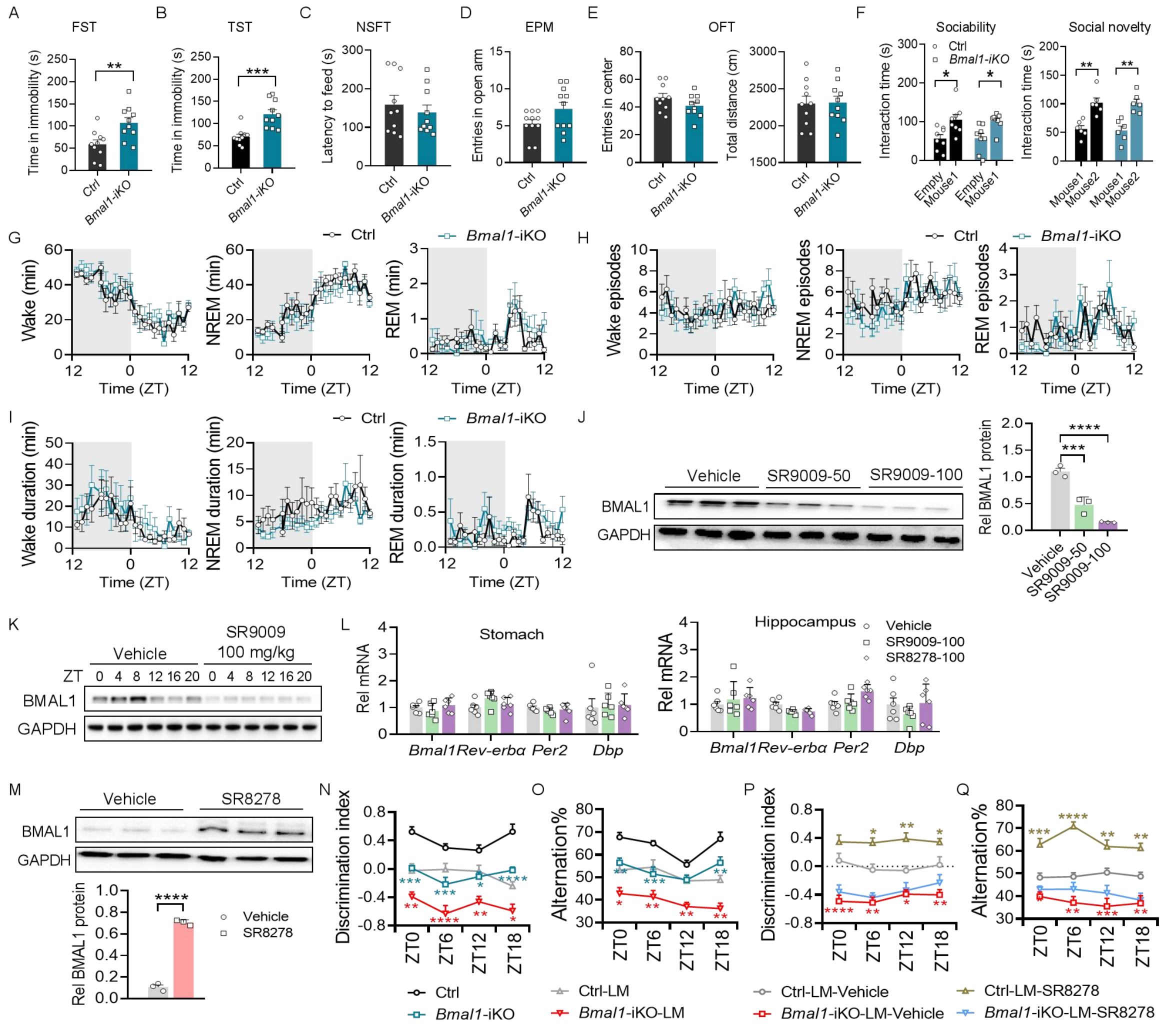

Figure S2 A, B, Immobility duration measured in (A) FST or (B) TST test in *Bmal1*-iKO and control mice (*n* = 10). C, Latency duration measured in NSFT test in *Bmal1*-iKO and control mice (*n* = 10). D, Open arm entries and duration measured in PMZ test in *Bmal1*-iKO and control mice (*n* = 10). E, Centre zone duration and locomotor activity measured in OFT test in *Bmal1*-iKO and control mice (*n* = 10). F, Social activity in *Bmal1*-iKO and control mice (*n* = 6~8). G, Daily patterns of wake, NREM, and REM sleep time for *Bmal1*-iKO and control mice (*n* = 8). H, Daily patterns of wake, NREM, and REM episodes for *Bmal1*-iKO and control mice (*n* = 8). I, Daily patterns of wake, NREM, and REM episode duration for *Bmal1*-iKO and control mice (*n* = 8). J, Effects of SR9009 on intestinal BMAL1 expression. Three of nine samples from nine mice were pooled to generate three biological replicates. K, Effects of SR9009 (100 mg/kg) on rhythmic BMAL1 expression in the intestine. For western blotting analysis of diurnal protein expression, three of nine samples from nine mice at each time point were pooled to generate three biological replicates. L, Effects of SR9009 or SR8278 on clock genes expression in the stomach and hippocampus (*n* = 6). M, Effects of SR8278 on intestinal BMAL1 expression. Three of nine samples from nine mice were pooled to generate three biological replicates. N, Diurnal changes in object performance for *Bmal1*-iKO and control mice (*n* = 7~8) after delirium induction. Significance differences between Ctrl-LM and *Bmal1*-iKO-LM, and between Ctrl and *Bmal1*-iKO at specific time points are indicated by stars. O, Diurnal changes in spontaneous alternations for *Bmal1*-iKO and control mice (*n* = 7~8) after delirium induction. Significance differences between

Ctrl-LM and *Bmal1*-iKO-LM, and between Ctrl and *Bmal1*-iKO at specific time points are indicated by stars. P, Diurnal changes in object performance for SR8278-treated *Bmal1*-iKO and control mice (n = 6) after delirium induction. Significance differences between Ctrl-LM-Vehicle and Ctrl-LM-SR8278, and between Ctrl-LM-Vehicle and *Bmal1*-iKO-LM-Vehicle at specific time points are indicated by stars. Q, Diurnal changes in spontaneous alternations for SR8278-treated *Bmal1*-iKO and control mice (n = 6) after delirium induction. Significance differences between Ctrl-LM-Vehicle and Ctrl-LM-SR8278, and between Ctrl-LM-Vehicle and *Bmal1*-iKO-LM-Vehicle at specific time points are indicated by stars. Discrimination indices were calculated as: (Time for novel object exploring – time for familiar object exploring)/(Time for novel object exploring + time for familiar object exploring). Behavioral tests (A-F) were conducted at ZT6. Data are mean ± SEM, and analyzed by two-tailed Student's t-test (A, B, F and M), one-way ANOVA with Bonferroni posttest (J) and two-way ANOVA with Bonferroni posttest (N-Q). *$p < 0.05$, **$p < 0.01$ and ***$p < 0.001$. FST, forced swimming test; NSFT, novelty-suppressed feeding test; OFT, open filed test; PMZ, elevated plus maze test; TST, tail suspension test.

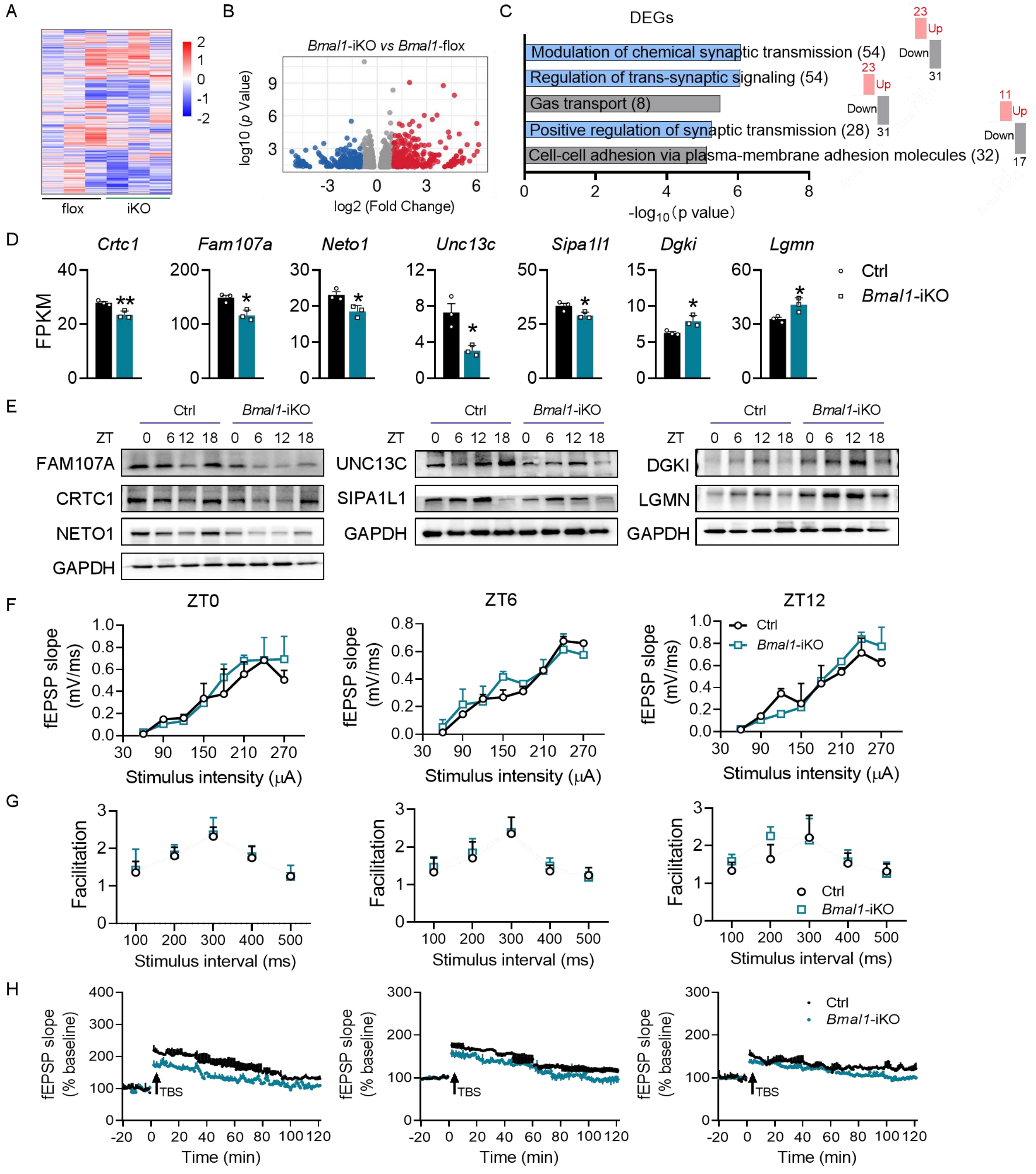

Figure S3 A, Heatmap for differentially expressed genes (DEGs) in the hippocampus caused by *Bmal1*-iKO. B, Volcano plot showing differential gene expression. C, GO enrichment analysis of hippocampal DEGs associated with *Bmal1*-iKO. A-C, three *Bmal1*-iKO mice and three control mice were sacrificed and hippocampal samples were collected at ZT6. D, Expression of genes related to synaptic plasticity in the hippocampus from *Bmal1*-iKO and control mice according to RNA-seq (n = 3). E, Immunoblotting of hippocampal proteins related to synaptic plasticity in *Bmal1*-iKO and control mice. For western blotting analysis of diurnal protein expression, three of nine samples from nine mice at each time point were pooled to generate three biological replicates. One representative blot is shown from three biological replicates. F, Input/output curves in hippocampal slices from *Bmal1*-iKO and control mice at ZT0, ZT6 and ZT12 (n = 3). G, Paired-pulse facilitation in hippocampal slices from *Bmal1*-iKO and control mice ZT0, ZT6 and

ZT12 (n = 3). H, Time course of fEPSP recordings in hippocampal slices from *Bmal1*-iKO and control mice after TBS at ZT0-2, ZT6-8 and ZT12-14 (n = 3). Data are mean ± SEM, and analyzed by two-tailed Student's t-test (D). *p < 0.05 and **p < 0.01.

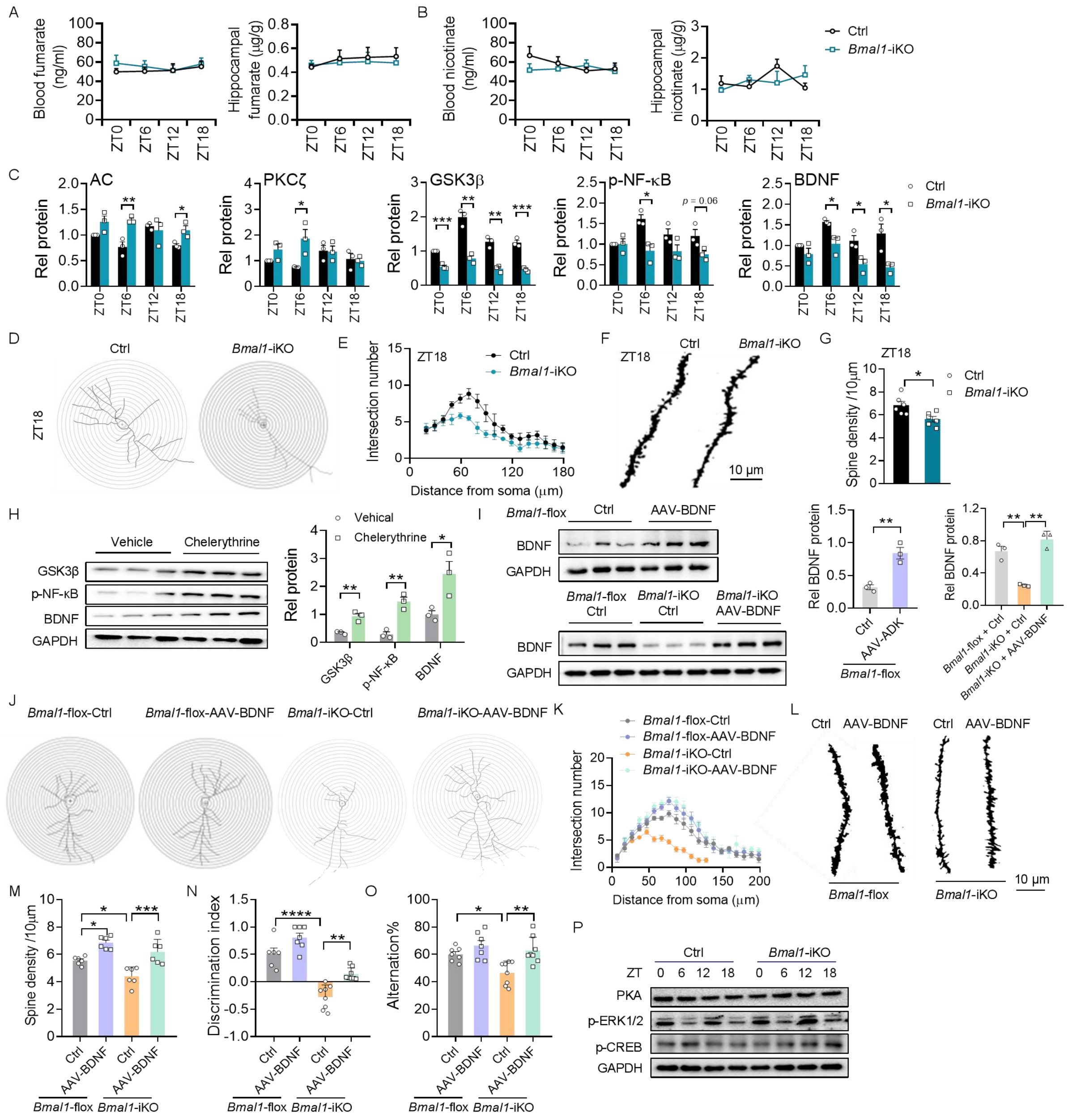

Figure S4 A, B, Fumarate (A) and nicotinate (B) concentrations in the blood and hippocampus at different time points in *Bmal1*-iKO and control mice (n = 6). C, Quantification of hippocampal enzymes involved in adenosine-A_1R signaling in Figure 3F (n = 3). D, Representative Golgi-stained CA1 pyramidal neurons at ZT18, showing decreased total dendrite length in *Bmal1*-iKO mice. E, Neurite arborization of CA1 pyramidal neurons in *Bmal1*-iKO and control mice at ZT18 (n = 6). F, Representative images of dendritic branches from Golgi-stained CA1 pyramidal neurons at ZT18 (scale bar, 10 μm). G, Spine density of the dendrites of CA1 pyramidal neurons in *Bmal1*-iKO and control mice at ZT18 (n = 6). H, Relative protein expression of GSK3β, p-NF-κB and BDNF in chelerythrine-treated HT22 cells (n = 3). I, Immunoblotting of hippocampal BDNF in *Bmal1*-iKO and control mice after virus injection. Three of nine samples from nine mice were pooled to generate three biological replicates. J, Representative Golgi-stained CA1 pyramidal neurons, showing increased total dendrite length in *Bmal1*-iKO mice and control after AAV-BDNF injection (n = 6). K, Neurite arborization of CA1 pyramidal neurons in *Bmal1*-iKO and

control mice after virus injection (*n* = 6). L, Representative images of dendritic branches from Golgi-stained CA1 pyramidal neurons (scale bar, 10 μm). M, Spine density of the dendrites of CA1 pyramidal neurons in *Bmal1*-iKO and control mice after virus injection (*n* = 6). N, Performance on NOR test during 2 h retention session in *Bmal1*-iKO and control mice after virus injection (*n* = 6~8). O, Spontaneous alternations in Y maze test in *Bmal1*-iKO and control mice after virus injection (*n* = 7~8). P, Relative protein expression of PKA, p-ERK1/2 and p-CREB in the hippocampus from *Bmal1*-iKO and control mice. For western blotting analysis of diurnal protein expression, three of nine samples from nine mice at each time point were pooled to generate three biological replicates. One representative blot is shown from three biological replicates. Discrimination indices were calculated as: (Time for novel object exploring – time for familiar object exploring)/(Time for novel object exploring + time for familiar object exploring). All behavioral tests were conducted at ZT6. In panels I-O, Ctrl is AAV-empty. Data are mean ± SEM, and analyzed by two-tailed Student's t-test (C and G-I), and two-way ANOVA with Bonferroni posttest (M-O). *$p < 0.05$, **$p < 0.01$, ***$p < 0.001$ and ****$p < 0.001$.

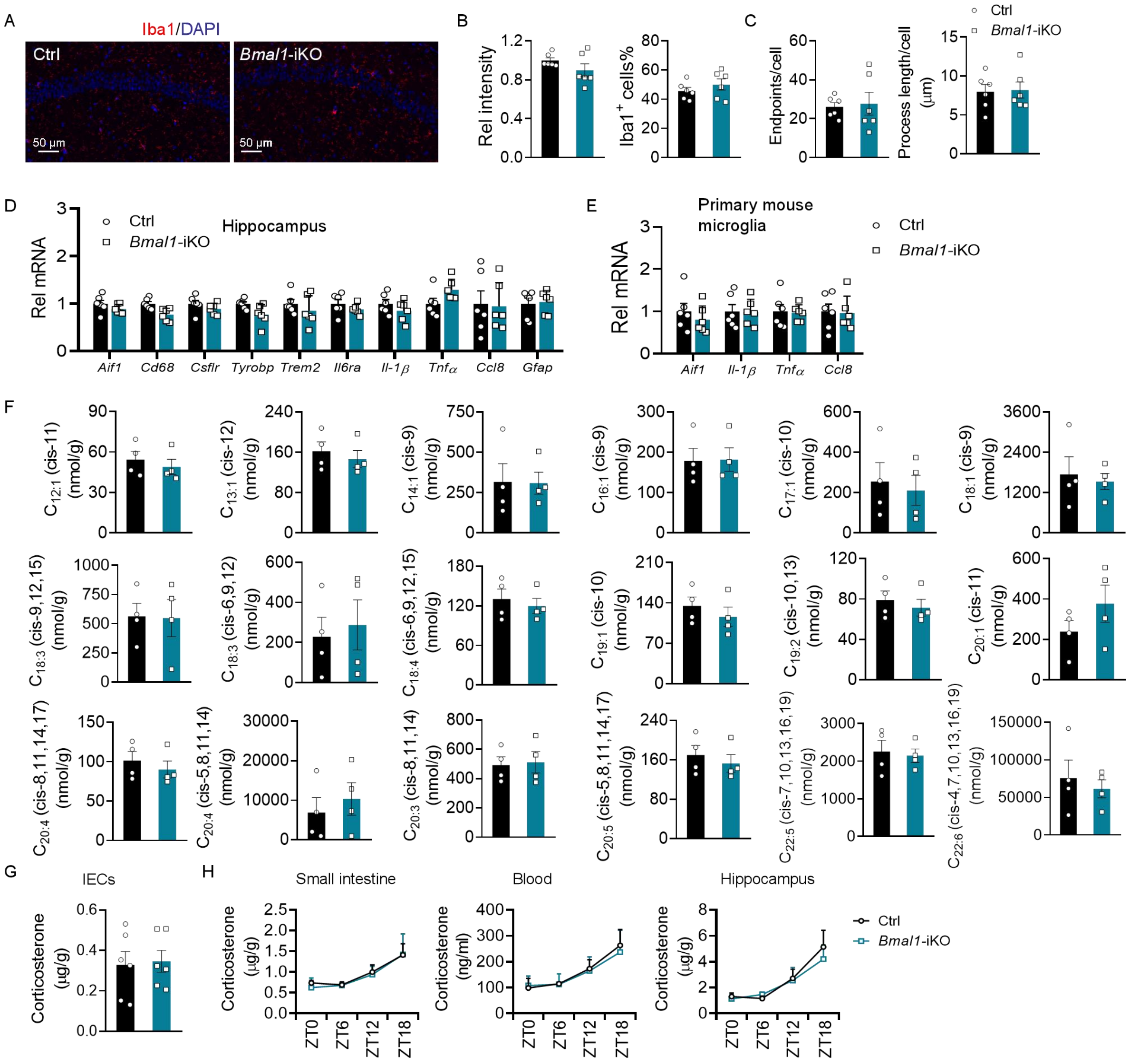

Figure S5 A, Immunofluorescent staining of hippocampus from *Bmal1*-iKO and control mice using the microglial marker Iba1 (red) and DAPI (blue). Scale bar, 50 μm. B, Quantification of Iba1$^+$ cells in the hippocampus of *Bmal1*-iKO and control mice (*n* = 6). C, Morphology analysis of Iba1$^+$ cells in the hippocampus of *Bmal1*-iKO and control mice (*n* = 6). D, Relative mRNA expression of genes involved in microglial activation from *Bmal1*-iKO and control mice (*n* = 6). E, Relative mRNA expression of genes involved in microglial activation in the primary microglial cells from *Bmal1*-iKO and control mice (*n* = 6). F, Amounts of various PUFAs in the hippocampus from *Bmal1*-iKO and control mice (*n* = 4). G, Corticosterone concentration of corticosterone intestinal epithelial cells (IECs) from *Bmal1*-iKO and control mice (*n* = 6) base on LC-MS/MS with multiple reaction monitoring. H, Diurnal corticosterone concentrations in small intestine, blood and hippocampus from *Bmal1*-iKO and control mice (*n* = 6). Data are mean ± SEM. PUFAs, polyunsaturated fatty acids.

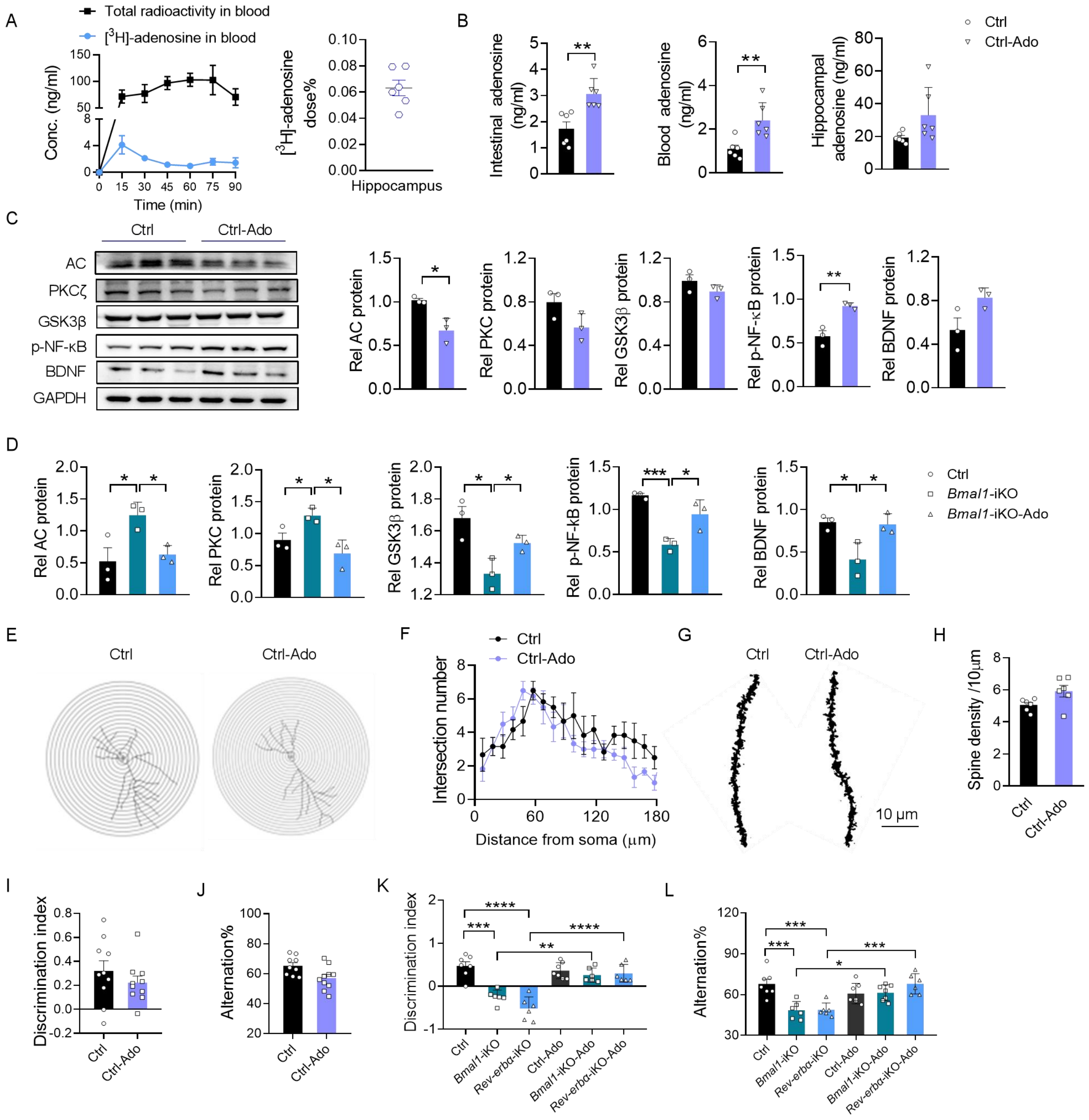

Figure S6 A, Blood concentrations of [^{3}H]-adenosine in wild-type mice after oral gavage (left, *n* = 6). [^{3}H]-adenosine amount in the hippocampus of wild-type mice after [^{3}H]-adenosine gavage (right, *n* = 6). B, Adenosine levels in the small intestine, blood and hippocampus from control (*Bmal1*-flox) mice gavaged with adenosine or vehicle (*n* = 6). C, Immunoblotting of hippocampal enzymes involved in adenosine-A$_1$R signaling in control mice gavaged with adenosine or vehicle. Three of nine samples from nine mice were pooled to generate three biological replicates. D, Quantification of hippocampal enzymes involved in adenosine-A$_1$R signaling in Figure 4B (*n* = 3). E, Representative Golgi-stained CA1 pyramidal neurons, showing unaltered total dendrite length in control mice gavaged with adenosine. F, Neurite arborization of CA1 pyramidal neurons in control mice gavaged with adenosine or vehicle (*n* = 6). G, Representative images of dendritic branches from Golgi-stained CA1 pyramidal neurons (scale bar, 10 μm). H, Spine density of the dendrites of CA1 pyramidal neurons in control mice gavaged with adenosine or vehicle (*n* = 6). I, Discrimination index in NOR test during 2 h retention session in control mice gavaged with adenosine or vehicle (*n* = 8). J, Spontaneous alternations in Y maze test in control mice gavaged

with adenosine or vehicle (*n* = 9~10). K, Discrimination index in NOR test during 2 h retention session in *Bmal1*-iKO, *Rev-erbα*-iKO and control mice supplemented with adenosine in the diet (*n* = 8~9). L, Spontaneous alternations in Y maze test in *Bmal1*-iKO, *Rev-erbα*-iKO and control mice supplemented with adenosine in the diet (*n* = 6~7). Discrimination indices were calculated as: (Time for novel object exploring – time for familiar object exploring)/(Time for novel object exploring + time for familiar object exploring). All behavioral tests were conducted at ZT6. Data are mean ± SEM, and analyzed by two-tailed Student's t-test (B-D), and two-way ANOVA with Bonferroni posttest (K and L). *$p < 0.05$, **$p < 0.01$, ***$p < 0.001$ and ****$p < 0.001$.

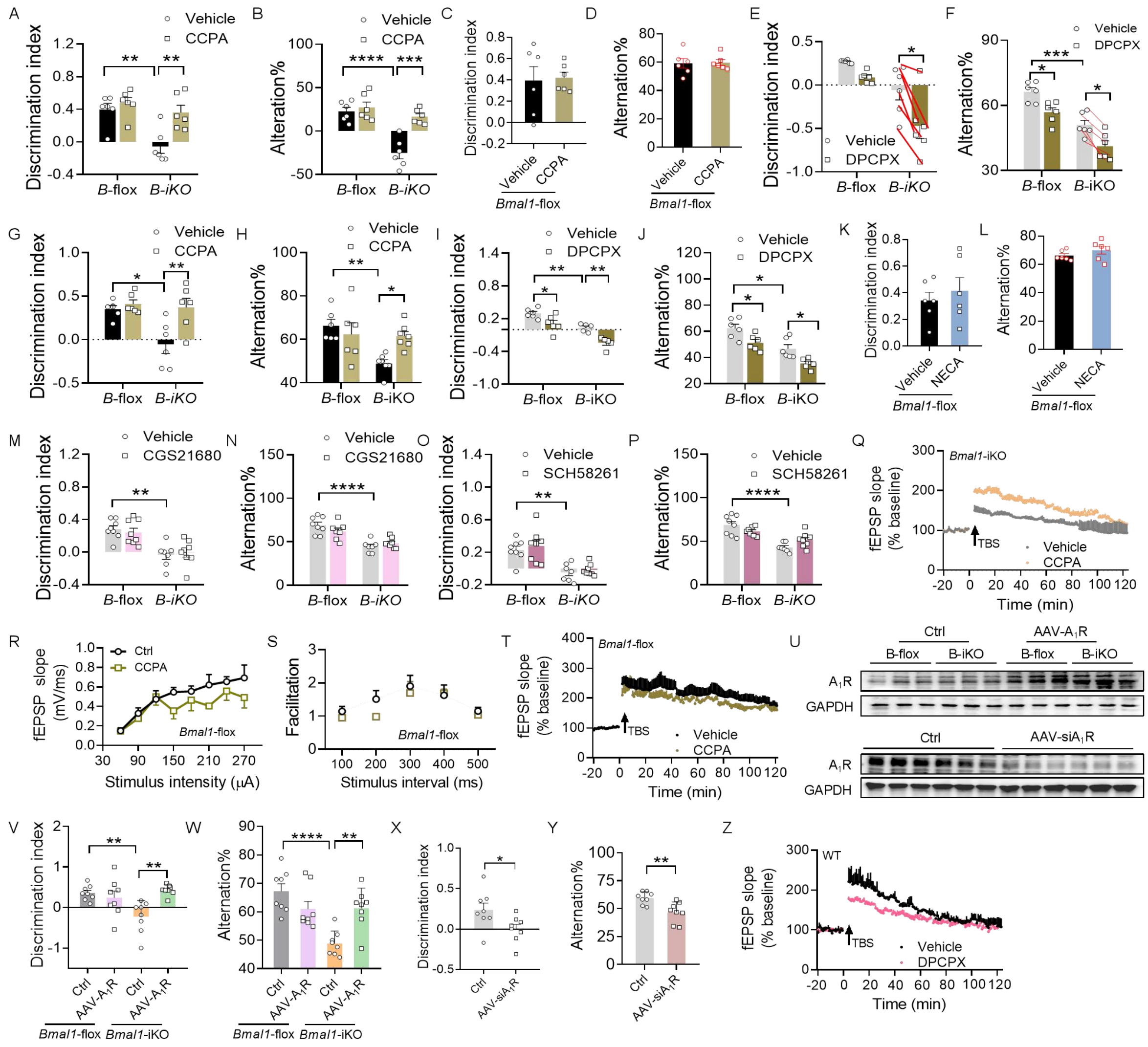

Figure S7 Involvement of adenosine-A₁R signaling in intestinal clock regulation of cognition. A, Discrimination index in NOR test during 24 h recall session in *Bmal1*-iKO and control mice treated with CCPA or vehicle (*n* = 6). B, Performance on SOL test in *Bmal1*-iKO and control mice treated with CCPA or vehicle (*n* = 6). C, Discrimination index in NOR test in control mice treated with CCPA or vehicle (*n* = 6). D, Spontaneous alternations in Y maze test in control mice treated with CCPA or vehicle (*n* = 6). E, Discrimination index in NOR test during 2 h retention session in *Bmal1*-iKO and control mice treated with DPCPX or vehicle (*n* = 6). F, Spontaneous alternations in Y maze test in *Bmal1*-iKO and control mice treated with DPCPX or vehicle (*n* = 6). G, Discrimination index in NOR test during 2 h retention session in *Bmal1*-iKO and control mice treated with CCPA or vehicle (intra-hippocampal injection, *n* = 6~8). H, Spontaneous alternations in Y maze test in *Bmal1*-iKO and control mice treated with CCPA or vehicle (intra-hippocampal injection, *n* = 6~7). I, Discrimination index in NOR test during 2 h retention session in *Bmal1*-iKO and control mice treated with DPCPX or vehicle (intra-hippocampal injection, *n* = 5~6). J, Spontaneous alternations in Y maze test in *Bmal1*-iKO and control mice treated with DPCPX or vehicle (intra-hippocampal injection, *n* = 6). K, Discrimination index in NOR test during 2 h retention session in control mice treated with NECA or vehicle (*n* = 6). L, Spontaneous alternations in Y maze test in control mice treated with NECA or vehicle (*n* = 6). M, Discrimination index in NOR test during 2 h retention session in *Bmal1*-iKO and control

mice treated with CGS21680 or vehicle (*n* = 7~8). N, Spontaneous alternations in Y maze test in *Bmal1*-iKO and control mice treated with CGS21680 or vehicle (*n* = 7~8). O, Discrimination index in NOR test during 2 h retention session in *Bmal1*-iKO and control mice treated with SCH58261 or vehicle (*n* = 7~8). P, Spontaneous alternations in Y maze test in *Bmal1*-iKO and control mice treated with SCH58261 or vehicle (*n* = 7~8). Q, Effect of CCPA (1 μM) on LTP induction in hippocampal slices from *Bmal1*-iKO mice (*n* = 3). R, Input/output curves in hippocampal slices from control mice treated with CCPA or vehicle (*n* = 3). S, Paired-pulse facilitation in hippocampal slices from control mice treated with CCPA or vehicle (*n* = 3). T, Time course of fEPSP recordings in hippocampal slices from control mice treated with CCPA or vehicle (*n* = 3). U, Relative A_1R proteins expression in the hippocampus of mice after injection of AAV-A_1R or AAV-shA_1R or control (*n* = 3~6). V, Discrimination index in NOR test in *Bmal1*-iKO and control mice after injection of AAV-A_1R or control (*n* = 8). W, Spontaneous alternations in Y maze test in *Bmal1*-iKO and control mice after injection of AAV-A_1R or control (*n* = 8). X, Discrimination index in NOR test in wild-type mice after injection of AAV-siA_1R or control (*n* = 8). Y, Spontaneous alternations in Y maze test in wild-type mice after injection of AAV-siA_1R or control (*n* = 8). Z, Effect of DPCPX (50 nM) on LTP induction in hippocampal slices from wild-type mice (*n* = 3). For panels E and F, the pairing of datapoints represent the same mice tested first vehicle and then DPXCP. Discrimination indices were calculated as: (Time for novel object exploring – time for familiar object exploring)/(Time for novel object exploring + time for familiar object exploring). All behavioral tests were conducted at ZT6. Data are mean ± SEM, and analyzed by two-way ANOVA with Bonferroni posttest (A, B, E-J, M-P, V and W), and two-tailed Student's t-test (X and Y). $*p < 0.05$, $**p < 0.01$, $***p < 0.001$ and $****p < 0.001$.

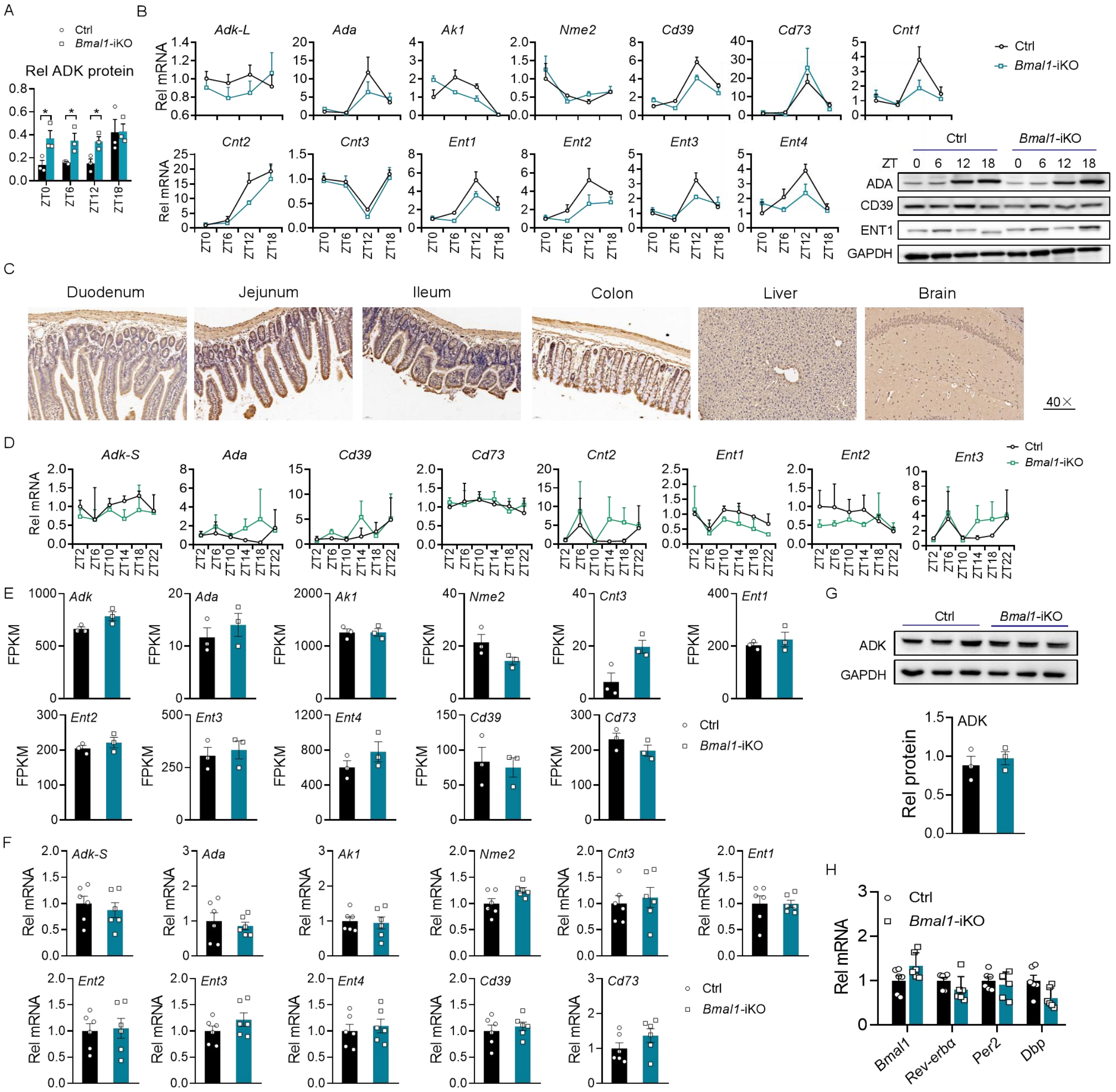

Figure S8 A, Quantification of intestinal ADK protein expression in Figure 5B (n = 3). B, Relative mRNA expression of adenosine-processing genes in the small intestine of *Bmal1*-iKO and control mice (n = 6, left). Relative expression of ADA, CD39 and ENT1 proteins in the small intestine of *Bmal1*-iKO and control mice (right). For western blotting analysis of diurnal protein expression, three of nine samples from nine mice at each time point were pooled to generate three biological replicates. One representative blot is shown from three biological replicates. C, Immunohistochemistry staining of ADK in representative sections of duodenum, jejunum, ileum, colon, liver and brain from wild-type mice. D, Relative mRNA expression of adenosine-processing genes in the liver of *Bmal1*-iKO and control mice (n = 3). E, Relative mRNA expression of adenosine-processing genes in the hippocampus of *Bmal1*-iKO and control mice according to RNA-seq (n = 3). F, Relative mRNA expression of adenosine-processing genes in the hippocampus of *Bmal1*-iKO and control mice according to qPCR (n = 6). G, Relative ADK protein expression in the hippocampus of *Bmal1*-iKO and control mice (n = 3). H, Relative mRNA expression of clock genes in the hippocampus of *Bmal1*-iKO and control mice (n = 6). Data are mean ± SEM, and analyzed by two-tailed Student's t-test (A). *$p < 0.05$.

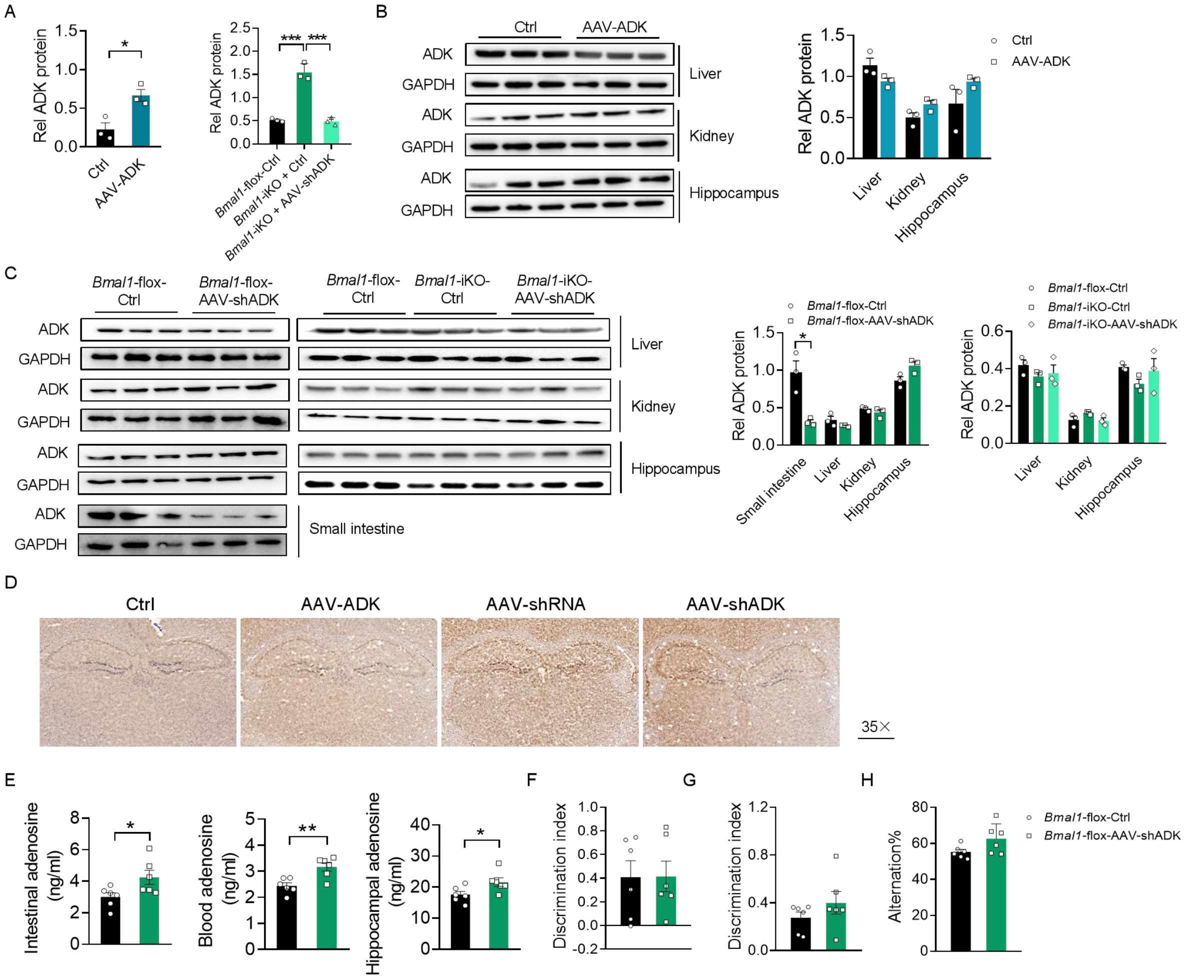

Figure S9 A, Quantification of intestinal ADK protein expression in Figure 5F (n = 3). B, Immunoblotting of ADK in the liver, kidney and hippocampus of wild-type mice after injection of AAV-ADK and control virus (n = 3). C, Immunoblotting of ADK in the liver, kidney, small intestine and hippocampus of *Bmal1*-iKO and control mice after injection of AAV-shADK and control virus. D, Immunohistochemistry staining of ADK in representative sections of brain from mice after injection of AAV-ADK or AAV-shADK or control virus. E, Adenosine levels in the small intestine, blood and hippocampus from *Bmal1*-flox mice after injection of AAV-shADK or control virus (n = 6). F, Discrimination index in NOR test during 2 h retention session in *Bmal1*-flox mice after injection of AAV-shADK or control virus (n = 6). G, Discrimination index in NOR test during 24 h recall session in *Bmal1*-flox mice after injection of AAV-shADK or control virus (n = 6). H, Spontaneous alternations in Y maze test in *Bmal1*-flox mice after injection of AAV-shADK and control virus (n = 6). Discrimination indices were calculated as: (Time for novel object exploring – time for familiar object exploring)/(Time for novel object exploring + time for familiar object exploring). All behavioral tests were conducted at ZT6. Data are mean ± SEM, and analyzed by two-tailed Student's t-test (A, C and E). *p < 0.05, **p < 0.01, ***p < 0.001 and ****p < 0.001.

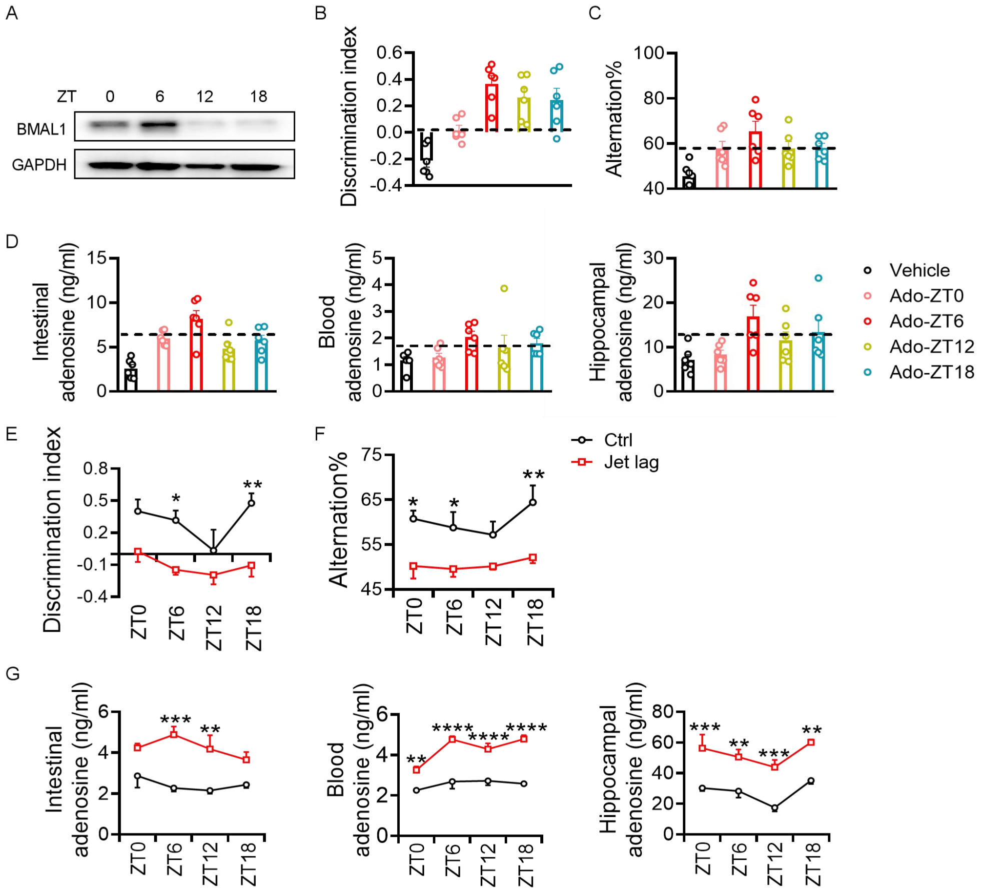

Figure S10 A, Diurnal expression of BMAL1 in the small intestine of 5×FAD mice. B, Time-of-day effects on object preference of adenosine supplementation in 5×FAD mice (n = 6). C, Time-of-day effects on spontaneous alternations of adenosine supplementation in 5×FAD mice (n = 6). D, Adenosine levels in 5×FAD mice after adenosine supplementation (n = 6). E, Discrimination index for jet-lagged and control mice (n = 6~8). F, Spontaneous alternations for jet-lagged and control mice (n = 6~8). G, Adenosine levels in jet-lagged and control mice (n = 6). Data are mean ± SEM, and analyzed by two-way ANOVA with Bonferroni posttest (E-G). *p < 0.05, **p < 0.01, ***p < 0.001 and ****p < 0.001.

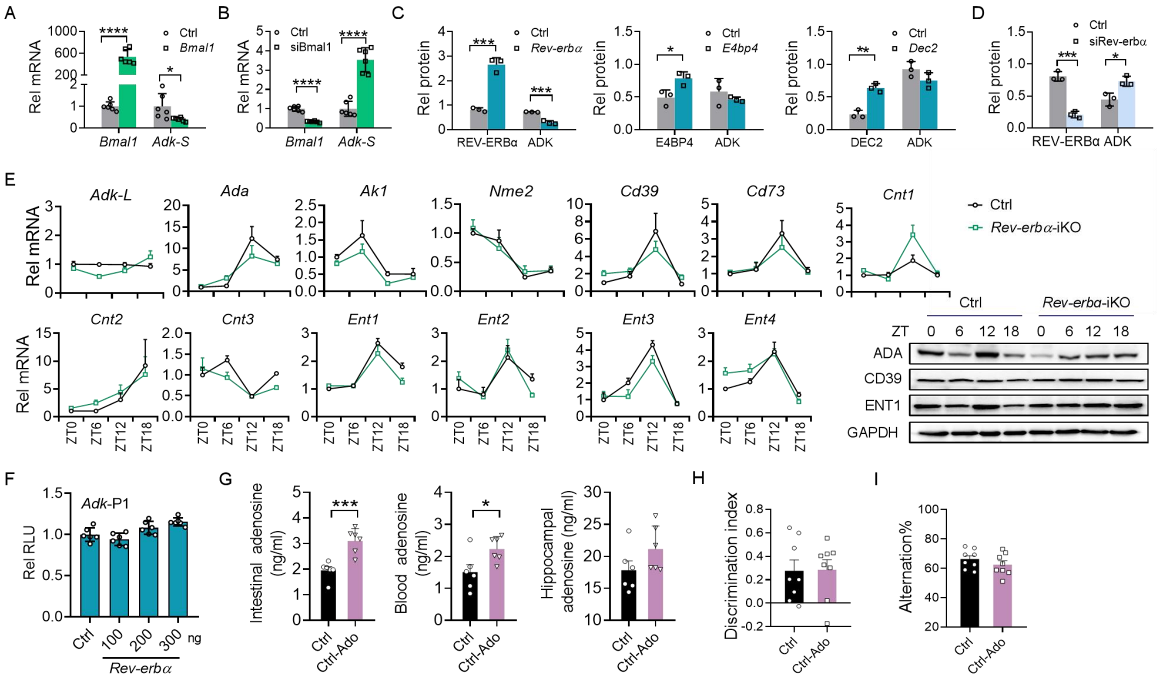

Figure S11 A, Effects of *Bmal1* overexpression on *Adk-S* expression (n = 6). B, Effects of *Bmal1* knockdown on *Adk-S* expression (n = 6). C, Effects of *Rev-erbα, E4bp4* or *Dec2* overexpression on ADK expression (n = 3). D, Effects of *Rev-erbα* knockdown on ADK expression (n = 3). E, Relative mRNA expression of adenosine-processing genes in the small intestine of *Rev-erbα*-iKO and control (*Rev-erbα*-flox) mice (n = 6, left). Relative expression of ADA, CD39 and ENT1 proteins in the small intestine of *Rev-erbα*-iKO and control mice (right). For western blotting analysis of diurnal protein expression, three of nine samples from nine mice at each time point were pooled to generate three biological replicates. One representative blot is shown from three biological replicates. F, Effects of *Rev-erbα* on *Adk*-P1 transcription in luciferase reporter assays (n = 6). G, Adenosine levels in the small intestine, blood and hippocampus from control mice gavaged with adenosine or vehicle (n = 6). H, Discrimination index in NOR test during 2 h retention session in control mice gavaged with adenosine or vehicle (n = 8). I, Spontaneous alternations in Y maze test in control mice gavaged with adenosine or vehicle (n = 8). Discrimination indices were calculated as: (Time for novel object exploring – time for familiar object exploring)/(Time for novel object exploring + time for familiar object exploring). All behavioral tests were conducted at ZT6. Data are mean ± SEM, and analyzed by two-tailed Student's t-test (A-D and G). *p < 0.05, **p < 0.01 and ***p < 0.001.

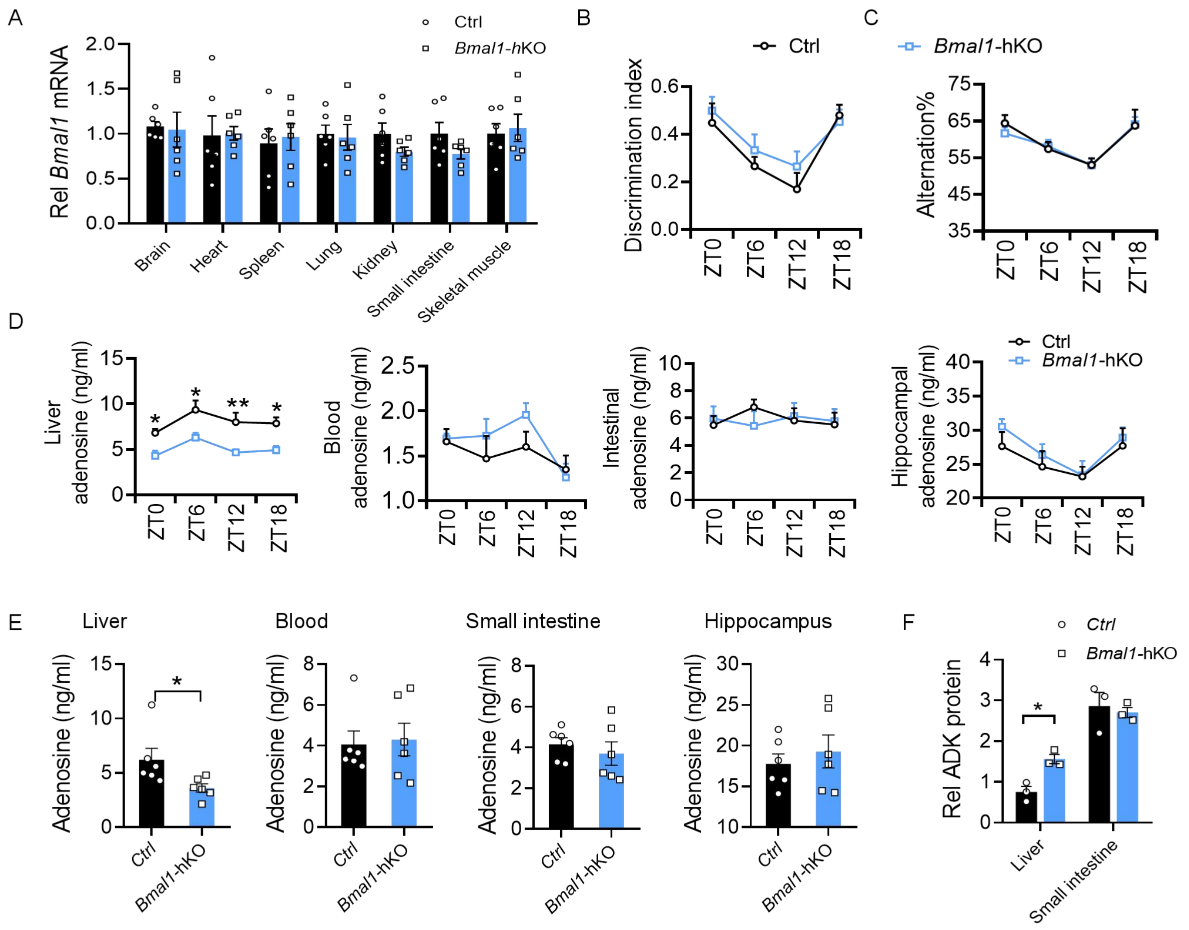

Figure 12 Liver clock does not affect cognitive function. A, Relative *Bmal1* mRNA expression in the brain, heart, spleen, lung, kidney, small intestine and skeletal muscle of *Bmal1*-hKO and control mice (n = 6). B, Diurnal changes in object preference for male *Bmal1*-hKO and control mice (n = 8). C, Diurnal changes in spontaneous alternations for male *Bmal1*-hKO and control mice (n = 8). D, Diurnal changes in adenosine levels in the liver, blood, small intestine and hippocampus from male *Bmal1*-hKO and control mice (n = 6). E, Adenosine levels in the liver, blood, small intestine and hippocampus from female *Bmal1*-hKO and control mice (n = 6). F, Relative ADK protein expression in the liver and small intestine from male *Bmal1*-hKO and control mice (n = 3). Discrimination indices were calculated as: (Time for novel object exploring – time for familiar object exploring)/(Time for novel object exploring + time for familiar object exploring). Data are mean ± SEM, and analyzed by two-way ANOVA with Bonferroni posttest (D), and two-tailed Student's t-test (E and F). *$p < 0.05$ and **$p < 0.01$.

SUPPLEMENTAL TABLES

Table 1. **Cosinor analyses of rhythmic genes, metabolites and behaviors in mice.** *$p < 0.05$, **$p < 0.01$, ***$p < 0.001$ and ****$p < 0.0001$; n.s., not significant.

	Mesor	Amplitude	Ψ (°)	*p*	Mesor	Amplitude	Ψ (°)	*p*
**Fig.2D**	***Bmal1*-flox**				***Bmal1*-iKO**			
Crtc1	*0.79*	*0.27*	*-340*	***	*0.42*	*0.17*	*-352*	***
Fam107a	*0.77*	0.28	-354	***	*0.40*	*0.20*	*-347*	***
Neto1	*20.9*	*0.20*	*-351*	**	*0.40*	*0.15*	*-320*	****
Unc13c	*1.11*	*0.08*	*-101*	n.s.	*0.64*	*0.11*	*-168*	n.s.
Sipa1l1	*1.70*	*0.11*	*-345*	n.s.	*0.85*	*0.21*	*-82.3*	n.s.
Dgki	*1.14*	*0.46*	*-124*	**	*2.54*	*1.05*	*-143*	****
Lgmn	*1.23*	*0.47*	*-134*	**	*3.13*	*0.97*	*-161*	****
**Fig.2I**	***Bmal1*-flox**				***Bmal1*-iKO**			
fEPSP slope (% baseline)	*153*	*25.6*	*-320*	***	*129.0*	*13.4*	*-313*	**
**Fig.2K**	***Bmal1*-flox**				***Bmal1*-iKO**			
fEPSP slope (% baseline)	*75.0*	*6.39*	*-149*	*	*76.1*	*5.41*	*-147*	**
**Fig.3C**	***Bmal1*-flox**				***Bmal1*-iKO**			
Adenosine	*10.7*	*0.69*	*-271*	n.s.	*2.52*	*0.78*	*-161*	n.s.
Fumarate	*4.22*	*0.12*	*-111*	n.s.	*3.29*	*0.22*	*-186*	n.s.
Nicotinate	*0.48*	*0.04*	*-287*	n.s.	*0.71*	*0.12*	*-96.5*	****
**Fig.3D**	***Bmal1*-flox**				***Bmal1*-iKO**			
Blood adenosine	*2.28*	*0.23*	*-12.0*	n.s	*1.21*	*0.04*	*-199*	n.s.
Hippocampal adenosine	*21.3*	*5.68*	*-281*	****	*9.12*	*4.50*	*-278*	****
**Fig.5B**	***Bmal1*-flox**				***Bmal1*-iKO**			
Adk-S	1.24	0.83	*-202*	*	4.13	2.86	*-200*	*
**Fig.6F**	***Rev-erbα*-flox**				***Rev-erbα*-iKO**			
Adk-S	1.79	1.61	*-189*	*	5.65	3.65	*-186*	**
**Fig.S1I**	***Bmal1*-flox**				***Bmal1*-iKO**			
Discrimination index	0.31	0.18	*-321*	****	-0.01	0.05	*-331*	****
**Fig.S1J**	***Bmal1*-flox**				***Bmal1*-iKO**			
Alternation%	60.4	4.26	*-8.50*	**	49.6	3.75	*-18.3*	*
**Fig.S1K**	***Bmal1*-flox**				***Bmal1*-iKO**			
Discrimination index	0.31	0.20	-322	***	-0.11	0.14	-335	***
**Fig.S1L**	***Bmal1*-flox**				***Bmal1*-iKO**			
Alternation%	56.1	5.57	*-342*	****	46.3	5.67	*-342*	****
**Fig.S2N**	***Bmal1*-flox**				***Bmal1*-iKO**			
Discrimination index	0.40	0.17	*-319*	****	-0.08	0.12	*-300*	**
**Fig.S2O**	***Bmal1*-flox**				***Bmal1*-iKO**			
Alternation%	63.8	6.16	*-351*	*	53.3	4.53	*-327*	***
**Fig.S5H**	***Bmal1*-flox (corticosterone)**				***Bmal1*-iKO (corticosterone)**			

Small intestine	0.96	0.38	*-250*	**	0.92	0.40	*-247*	*
Blood	162	82.8	*-243*	*	155	68.2	*-246*	***
Hippocampus	2.58	2.10	*-251*	**	2.34	1.53	*-242*	*
**Fig.S8B**	***Bmal1*-flox (hippocampus)**				***Bmal1*-iKO (hippocampus)**			
Adk-L	*0.98*	*0.03*	*-139*	n.s.	0.90	0.14	*-282*	***
Ada	4.26	5.55	*-195*	*	3.32	3.06	*-221*	**
Ak1	1.17	1.06	*-105*	****	1.02	0.82	*-48.7*	*
Nme2	1.39	0.50	*-114*	***	0.73	0.24	*-115*	*
Cd39	2.92	2.56	*-199*	**	2.23	1.45	*-214*	*
Cd73	6.52	8.76	*-193*	*	*7.72*	*12.4*	*-187*	n.s.
Cnt1	1.75	1.46	*-195*	*	*1.27*	*1.32*	*-216*	n.s.
Cnt2	9.64	11.1	*-229*	***	7.06	8.32	*-244*	**
Cnt3	*0.85*	*0.32*	*-346*	n.s.	*0.77*	*0.37*	*-348*	n.s.
Ent1	2.62	2.16	*-193*	**	1.87	1.42	*-208*	**
Ent2	2.97	2.31	*-204*	****	1.83	1.27	*-232*	****
Ent3	1.56	1.21	*-201*	*	*1.41*	0.65	*-223*	*
Ent4	2.09	1.50	*-166*	**	*1.61*	0.34	*-176*	n.s.
**Fig.S8D**	***Bmal1*-flox (liver)**				***Bmal1*-iKO (liver)**			
Adk-S	*1.00*	*0.23*	*-247*	**	0.79	0.06	*-264*	n.s.
Ada	*0.92*	*0.49*	*-29.9*	*	1.64	0.48	*-262*	n.s.
Cd39	*2.02*	*1.56*	*-309*	*	2.77	1.24	*-261*	n.s.
Cd73	*1.05*	*0.15*	*-144*	****	1.09	0.10	*-134*	n.s.
Cnt2	*2.08*	*1.41*	*-39.3*	n.s.	4.59	0.72	*-239*	n.s.
Ent1	0.88	0.17	*-205*	n.s.	*0.64*	*0.11*	*-92.6*	n.s.
Ent2	0.78	0.26	*-119*	*	*0.55*	*0.09*	*-206*	n.s.
Ent3	1.96	0.82	*1.42*	n.s.	*2.81*	*0.69*	*-286*	n.s.
**Fig.S10E**	**Ctrl**				**Jetlag**			
Discrimination index	0.31	0.20	*-336*	*	-0.11	*0.11*	-349	**
**Fig.S10F**	**Ctrl**				**Jetlag**			
Alternation%	*60.3*	3.35	-302	*	*50.5*	*1.29*	*-271*	**
**Fig.S10G**	**Ctrl**				**Jetlag**			
Hippocampal adenosine	*27.8*	*7.25*	-332	*	*52.7*	*7.85*	*-322*	*
**Fig.S11E**	***Rev-erbα*-flox**				***Rev-erbα*-iKO**			
Adk-L	0.98	0.03	*-76.3*	n.s.	0.86	0.34	*-277*	***
Ada	5.53	6.45	*-208*	***	4.75	3.92	*-206*	****
Ak1	0.91	0.61	*-66.4*	**	0.65	0.47	*-52.4*	**
Nme2	0.62	0.46	*-34.6*	****	0.63	0.42	*-27.0*	**
Cd39	2.62	2.98	*-171*	*	2.68	1.43	*-166*	n.s.
Cd73	1.69	1.16	*-178*	*	1.51	0.72	*-171*	*
Cnt1	1.23	0.44	*-179*	*	*1.65*	*1.08*	*-189*	n.s.
Cnt2	3.56	4.22	*-256*	*	3.98	2.96	*-241*	*
Cnt3	0.97	0.30	*-31.8*	n.s.	0.82	0.35	*-20.2*	****
Ent1	1.64	0.89	*-202*	**	1.43	0.59	*-187*	*
Ent2	1.32	0.63	*-206*	*	*1.32*	*0.52*	*-184*	n.s.
Ent3	2.03	1.78	*-159*	*	*1.63*	*1.08*	*-168*	n.s.
Ent4	1.34	0.71	*-161*	*	*1.50*	*0.66*	*-121*	n.s.

Fig.S12B	***Bmal1*-flox**				***Bmal1*-hKO**			
Discrimination index	0.34	0.18	-322	***	*0.39*	*0.13*	*-333*	****
**Fig.S12C**	***Bmal1*-flox**				***Bmal1*-hKO**			
Alternation%	59.6	6.50	*-331*	***	*59.2*	*5.24*	*-327*	*
**Fig.S12D**	***Bmal1*-flox**				***Bmal1*-hKO**			
Liver adenosine	*7.80*	*0.96*	*-129*	n.s.	*5.03*	*0.72*	*-105*	n.s.
Blood adenosine	*1.52*	*0.07*	*-64.2*	n.s.	*1.66*	*0.27*	*-119*	n.s.
Intestine adenosine	*5.91*	*0.66*	*-105*	n.s.	*5.85*	*0.20*	*-244*	n.s.
Hippocampal adenosine	25.8	2.71	-325	***	*27.3*	*3.79*	*-340*	****

Table 2. List of Primers

Genes	Forward (5'-3' Sequence)	Reverse (5'-3' Sequence)
Ada	AGCGCACTTCCACATAGACC	CCAGACACCCGCATTCAA
Adk-L	CCAAGCCCAAAAAGCTCAAGG	GTCATCTCGCCCTTGTGTGA
Adk-S	TCATGGGGATGAGACCTGGAG	ACAAGCACACTCTTGGGAGAC
Adora1	TGTGCCCGGAAATGTACTGG	TCTGTGGCCCAATGTTGATAAG
Adora2a	GCCATCCCATTCGCCATCA	GCAATAGCCAAGAGGCTGAAGA
Adora2b	AGCTAGAGACGCAAGACGC	GTGGGGGTCTGTAATGCACT
Adora3	AAGGTGAAATCAGGTGTTGAGC	AGGCAATAATGTTGCACGAGT
Ak1	CCGCCCACCACAAAGATG	GAGAAGCTGAAGAAGGCCAAGA
Aif1	CTTGAAGCGAATGCTGGAGAA	GGCAGCTCGGAGATAGCTTT
Ccl8	TCTACGCAGTGCTTCTTTGCC	AAGGGGGATCTTCAGCTTTAGTA
Cd39	AAGGTGAAGAGATTTTGCTCCAA	TTTGTTCTGGGTCAGTCCCAC
Cd73	ACGTGCTGTTTTTGGATGCC	TCACCGCCAACAGAGAGAAC
Cnt1	GGGATGCTACAAGTCCTGCC	TAGCCTCAGTTTCCCCGGTT
Cnt2	ACAAGATATGGAGAAGTCAAAGGG	TGGAGCAGGCAAAGAGGATG
Cnt3	GACCTTGAACGGCAGAACACT	CTTTGTTTCCTAGAGGCTCCTG
Crtc1	GCATCGTGAGCTTTGGTTCC	GATGGGCGTCGAGACTTCTG
Csflr	TGTCATCGAGCCTAGTGGC	CGGGAGATTCAGGGTCCAAG
Dec2	CCCTCATTTGCAAGAGAGACAG	AGGTATCCTTGGTATCGTCTCG
Dgki	GGATGGATGCTGCGGGAAG	GGACTTGGCAGTTCTCCTCC
E4bp4	TATTGGGAGAAACGGCGGAAA	AGCCTTGGATGTCTGGTAGTC
Ent1	CAGCCTCAGGACAGGTATAAGG	GTTTGTGAAATACTTGGTTGCGG
Ent3	GAGCCCCGAGTAACCATCAAG	CATTGAAGCGATCCTCTGGC
Ent4	CACAGACTCTGCGGTAGAGGA	CCTGGGTACTTGTGGTGAAGATA
Fam107a	GTTAGGCTGACTGTGTTCCAA	CAGCAAAGAGTCTCAGGGAGG
Gfap	CCCTGGCTCGTGTGGATTT	GACCGATACCACTCCTCTGTC
Il-1β	GGCTGTATTCCCCTCCATCG	AAGGTGCTGATCTGGGTTGG
Il6ra	TAGTCCTTCCTACCCCAATTTCC	TTGGTCCTTAGCCACTCCTTC
Lgmn	TATTCTTTACTCTGGGTCTGCAA	AGACCCTTTGCCCTTCACAG
Neto1	CTCTCACTTGGTAACTCTGCCA	GGGGAAAGCGACAGAGGAAA
Nme2	CTGCCTGTGAGAACAAGAGTAA	AAGCCTGGGACCATACGA
Rev-erbα	TTTTTCGCCGGAGCATCCAA	ATCTCGGCAAGCATCCGTTG
Sipa1l1	AGTAAGGACCCCTCTCCCAC	GAGAGCATTTACCTTCTTCAAATCT
Tnfα	TTTTTCGCCGGAGCATCCAA	ATCTCGGCAAGCATCCGTTG
Trem2	CTGGAACCGTCACCATCACTC	CGAAACTCGATGACTCCTCGG
Tyrobp	CCCAAGATGCGACTGTTCTTC	GTCCCTTGACCTCGGGAGA
Unc13c	AAAGCGAACTGCAAAGTGATGA	TGCTCTCTGAAGAGTAGATCCAA
Ppib	TCCACACCCTTTTCCGGTCC	CAAAAGGAAGACGACGGAGC
β-actin	GGCTGTATTCCCCTCCATCG	CCAGTTGGTAACAATGCCATGT

Table 3. List of Sequences

**Name**	**Forward (5'-3' Sequence)**	**Reverse (5'-3' Sequence)**
siRNA		
si*Bmal1*	GCUCUUUCUUCUGUAGAAUTT	AAAGAUAUCUUCCCUCGGTT
si*Rev-erbα*	CUUCGUUGUUCAACGUGAATT	UUCACGUUGAACAACGAAGTT
si*Adora1*	CCUCAUCUACAUUGCCAUCUUTT	AAGAUGGCAAUGUAGAUGAGGTT
Control	UUCUCCGAACGUGUCACGUTT	ACGUGACACGUUCGGAGAATT
shRNA	**Sequence**	
Adk shRNA	CCGGGCACATGTGGATGCTCATTACCTCGAGGTAATGAGCATCCACATGTG CTTTTTT	
ChIP		
Adk (REV-ERBα)	TAGCAGCCGCAGAGGCACCA	TCCAAGCTCCGCCCAATCC
